# Position-Selective Synthesis and Biological Evaluation of Four Isomeric A-Ring Amino Derivatives of the Alkaloid Luotonin A

**DOI:** 10.3390/molecules24040716

**Published:** 2019-02-16

**Authors:** Amra Ibric, Stefan Eckerstorfer, Martin Eder, Ivan Louko, Leopold Tunjic, Petra Heffeter, Hemma Henrike Schueffl, Brigitte Marian, Norbert Haider

**Affiliations:** 1 Department of Pharmaceutical Chemistry, University of Vienna, Althanstraße 14, A-1090 Vienna, Austria; amra.ibric@univie.ac.at (A.I.); stefan.eckerstorfer@me.com (S.E.); semrad@gmx.at (M.E.); ivan_louko@yahoo.com (I.L.); leopold.tunjic@gmail.com (L.T.); 2 Institute of Cancer Research and Comprehensive Cancer Center, Medical University of Vienna, Borschkegasse 8a, A-1090 Vienna, Austria; petra.heffeter@meduniwien.ac.at (P.H.); hemma.schueffl@meduniwien.ac.at (H.H.S.); brigitte.marian@meduniwien.ac.at (B.M.)

**Keywords:** Luotonin A, quinazoline, quinoline, cycloaddition, catalytic transfer hydrogenation, Topoisomerase I, cytotoxic activity

## Abstract

Following two orthogonal synthetic routes, a series of all four possible A-ring amino derivatives of the natural product Luotonin A (a known Topoisomerase I inhibitor) was synthesized. In both strategies, intramolecular cycloaddition reactions are the key step. The target compounds were obtained in good yields by mild catalytic transfer hydrogenation of the corresponding nitro precursors. In-vitro evaluation of the antiproliferative activity towards human tumor cell lines revealed the 4-amino compound (**5b**) to be the most effective agent, showing an interesting profile of cytotoxic activity. Among other effects, a significant G2/M cell cycle arrest was observed for this compound, suggesting that either Topoisomerase I is not the only biological target, or that some atypical mechanism is responsible for inhibition of this enzyme.

## 1. Introduction

**Figure 1:**
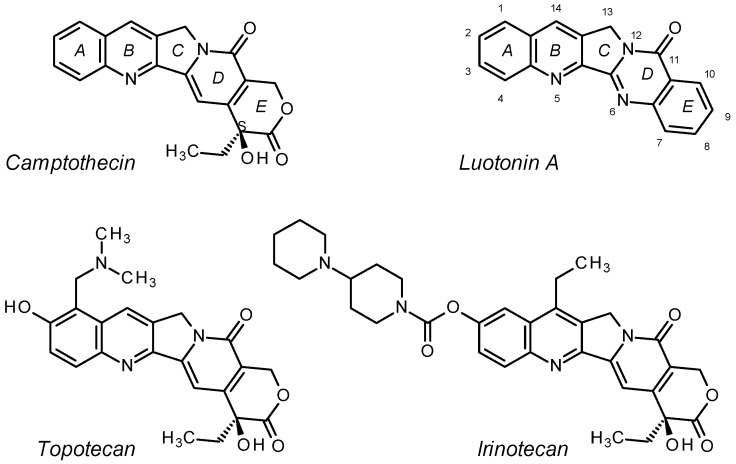
Structures of Camptothecin (CPT), Luotonin A (with IUPAC-conformant numbering scheme) and the CPT-derived drugs, Topotecan and Irinotecan.

DNA Topoisomerase I (Top1) represents an attractive target for cancer chemotherapy [1], as this enzyme plays a crucial role in the DNA replication process by relaxing the nucleic acid’s supercoiled structure. Based on the natural product, Camptothecin (CPT) as lead structure (Figure 1), highly effective Top1 inhibitors have been developed [2] that are clinically used for the treatment of various malignancies: Topotecan [3] has been approved as a drug against ovarian cancers and small-cell lung cancers, and Irinotecan [4] is therapeutically used against colorectal tumors [2]. With regard to their biochemical mode of action, drugs of this type are known to form a stable ternary complex with the covalent adduct of DNA and Top1 [5,6], thus blocking DNA replication efficiently by causing irreversible DNA damage upon collision of the ternary complex with the progressing replication fork [7]. 

Among various efforts to circumvent severe side-effects of these drugs, especially their bladder toxicity due to labile lactone motif, through chemical stabilization of the E-ring [1], the discovery of the alkaloid Luotonin A and its Top1-inhibitory activity [8] has opened a new perspective for the development of potentially useful new members of this drug family. As shown in Figure 1, the structures of CPT and Luotonin A are quite similar, differing mainly in the nature of ring E, which is a simple benzene ring in Luotonin A. Despite lacking the lactone functionality of CPT, Luotonin A is able to stabilize the DNA/Top1 covalent complex in an analogous manner as CPT, albeit at a significantly lower level of activity [9]. Thus, the search for new Luotonin A derivatives with a higher Top1-poisoning activity (comparable to that of CPT) has become a challenging topic for various research groups and it has stimulated the development of different synthetic strategies to access compounds of the quino[2′,3′:3,4] pyrrolo[2,1-*b*]quinazolin-11(13*H*)-one type. 

In this context, it should be noted that there are indications that Topoisomerase I may not be the only biological target that is involved in the cytotoxic activity of Luotonin-A-derived compounds [10,11]. Also for Evodiamine [12], a pentacyclic alkaloid with high structural similarity to Luotonin A, there have been reports describing Top1 inhibition as well as other cytotoxic effects that are associ-ated with a G2/M cell cycle arrest rather than with the CPT-typical effect on the S-phase [13,14,15].

A systematic overview of the main synthetic routes to Luotonin A is given in a review article by Liang et al. [8]. Among the more recent approaches, two orthogonal pathways have been shown by our group to be suitable for the specific placement of substituents at all possible positions of ring A: the “southern route” gives access to either 2- or 4-substituted Luotonin A derivatives [16,17,18] whereas the “northern route” leads to either 1- or 3-susbtituted congeners [19]. In both reaction sequences, intramolecular [4 + 2] cycloadditions represent the key step. 

As observed previously [11,20], many derivatives of the large and planar quino[2′,3′:3,4] pyrrolo[2,1-*b*]quinazolin-11(13*H*)-one system are characterized by low solubility in common solvents (especially in water) and thus we frequently encountered solubility problems on attempted biological evaluation of such compounds. Therefore, we decided to focus on Luotonin A derivatives with substituents capable of improving water solubility. In this context, the NH_2_ group appears particularly interesting, not only for its hydrophilic nature, but also for its potential to enhance drug/target binding affinity by enabling additional contacts (e.g., via hydrogen bonding). So far, only two Luotonin A derivatives with a primary amino group at ring E have been described in literature (7-amino-Luotonin A [11], 8-amino-Luotonin A [20]). In the present article, we report the synthesis and biological evaluation of four novel isomeric congeners bearing an amino group at ring A, namely at positions 1, 2, 3, and 4. 

## 2. Results and Discussion

### 2.1. Chemistry

**Scheme 1:**
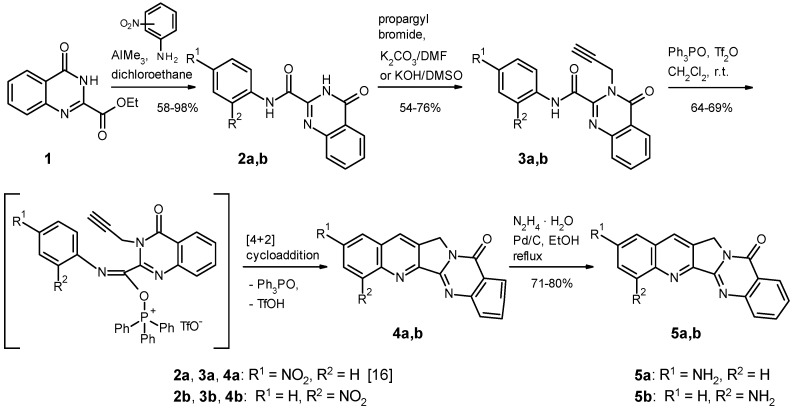
Synthesis of 2-nitro-Luotonin A (**4a**) [16] and 4-nitro-Luotonin A (**4b**) and their reduction into 2-amino-Luotonin A (**5a**) and 4-amino-Luotonin A (**5b**).

For the synthesis of the target Luotonin A derivatives featuring a primary amino function at ring A, the corresponding nitro compounds were envisaged as suitable precursors. Out of the four possible A-ring nitro isomers, only the 2-nitro compound [16] has been known to date: our group had prepared this Luotonin A derivative via a pathway that was based on the cycloaddition strategy previously reported by Zhou et al. [21] for the synthesis of the parent natural product. As outlined in Scheme 1, this pathway (referred to as the “southern route”) should be principally suitable to give access also to the hitherto unknown 4-nitro derivative as a pure isomer, starting from a 2-nitroaniline building block instead of a 4-nitroaniline synthon. Indeed, this proved to be the case: although acylation of a 2-nitroaniline NH_2_ group is somewhat challenging for both electronic and steric reasons, the AlMe_3_-mediated coupling reaction known as Weinreb amidation [22] permitted the smooth transformation of the quinazolinone ester **1** [23] (representing the D and E ring fragment of the target structure) into the ortho-substituted anilide **2b**. This intermediate, however, turned out to be almost insoluble in common solvents (in contrast to its known isomer **2a** [16]) and thus required some optimization in the subsequent alkylation reaction with propargyl bromide, which is necessary to introduce the requisite three-carbon fragment containing the dienophile for the following step. We succeeded in this alkylation to obtain compound **3b** by applying a protocol that we had recently used for similarly insoluble substrates [17] (DMSO as solvent, finely powdered potassium hydroxide, tetrabutylammonium bromide as phase-transfer catalyst, ultrasound irradiation at room temperature). Assembly of the pentacyclic skeleton (**4b**) then was easily accomplished by intramolecular [4 + 2] cycloaddition, mediated by bis(triphenyl)oxodiphosphonium triflate (Hendrickson’s reagent) [24] in dry dichloromethane at room temperature, conditions that had been previously applied for analogous transformations [16,17,18,21]. 

With the 2-nitro compound **4a** [16] and the new 4-nitro isomer **4b** in hands, preparation of the corresponding two amino-substituted Luotonin A derivatives should be feasible in a straightforward manner by reduction of the NO_2_ group. While Cagir et al. [20] had obtained 8-amino-Luotonin A from the 8-nitro precursor by reduction with SnCl_2_ in moderate yield (40%) and Dallavalle et al. [25] had reported an unsuccessful attempt to selectively reduce the nitro group in 9-nitro-Luotinin A by catalytic hydrogenation in acetic acid, we decided to apply a protocol that had been successfully used for the reduction of nitrocarbazoles into aminocarbazoles [26,27]. This method makes use of hydrazine hydrate as hydrogen source in a catalytic transfer hydrogenation reaction in alcoholic solution or suspension at elevated temperature. Indeed, when **4a** or **4b**, respectively, were subjected to these conditions, the target amino compounds **5a** or **5b**, respectively, were formed very cleanly in good yields (70–80%). It should be noted that this transformation works very well despite the low solubility of the substrate (**4a**,**b**) in boiling ethanol. As there are no non-volatile reagents involved and no non-volatile by-products formed, work-up requires only removal of the catalyst by filtration after an ethanol/DMF solvent exchange, followed by precipitation upon dilution with water (see Experimental Section).

**Scheme 2:**
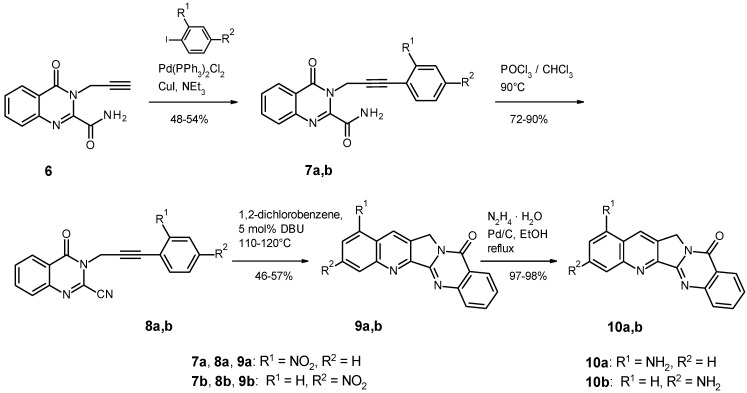
Synthesis of 1-nitro-Luotonin A (**9a**) and 3-nitro-Luotonin A (**9b**) and their reduction into 1-amino-Luotonin A (**10a**) and 3-amino-Luotonin A (**10b**).

For the synthesis of the other two amino-substituted target compounds (**10a,b**), the “northern route” seemed ideally suited, again making use of an intramolecular cycloaddition reaction to assemble the polycyclic skeleton. As demonstrated previously [19], the requisite arylpropargyl-substituted quinazolinonecarbonitriles as cycloaddition substrates are accessible in two steps from the carboxamide **6** (which can be prepared by ammonolysis of the ester **1** [28], followed by selective quinazolinone N-3 propargylation [29]) via Sonogashira coupling of the terminal alkyne with an appropriate aryl iodide and subsequent dehydration of the amide function into a nitrile. The latter step turned out to be unexpectedly challenging for the nitro compounds **7a**,**b** that were obtained from the propargyl compound **6**. In contrast to our previous experiences with structurally related compounds [19], employment of a dehydrating agent in combination with a base (trifluoroacetic anhydride/triethylamine [30] or ethyl dichlorophosphate/DBU [31]) resulted in rapid resinification. However, heating of the primary amides **7a**,**b** with phosphorus oxychloride in chloroform solution under carefully controlled conditions finally afforded the desired carbonitriles **8a**,**b** in good yields (Scheme 2). For both isomers, ring closure into the target quino[2′,3′:3,4]pyrrolo[2,1-*b*]quinazoline system was smoothly accomplished by a thermally induced intramolecular [4 + 2] cycloaddition reaction in the presence of catalytic amounts of DBU in analogy to lit. [19,32]. 

The resulting nitro-substituted Luotonin A derivatives **9a**,**b** precipitated from the reaction solvent (1,2-dichlorobenzene) and were isolated simply by filtration. Reduction of the nitro group, applying the same method as described above for **5a**,**b**, afforded the target 1-amino or 3-amino-substituted Luotonin A derivatives **10a**,**b** in nearly quantitative yields.

All new compounds are fully characterized with ^1^H-NMR, ^13^C-NMR, EI-MS and elemental analyses or high-resolution MS (ESI-TOF). In all final compounds (**5a**,**b** and **10a**,**b**), the presence of an amino group at ring A instead of a nitro group is reflected in their ^1^H-NMR spectra by a marked upfield shift that is observed for the proton signals belonging to rings A and B, as compared to the spectra of the nitro compounds (**4a**,**b** and **9a**,**b**). Assignment of all proton signals rests on COSY and 1D-NOE or NOESY spectra. The carbon signals of compounds **5a**,**b** and **10a**,**b** (which are much better soluble in DMSO-*d*_6_ than their precursors) were assigned by means of HSQC and HMBC experiments; for a complete signal listing, see Table 1. 

### 2.2. Biological Evaluation

While the nitro compounds of type **4** and **9** were not considered for any biological evaluation because of their very low water solubility, the new amino-substituted Luotonin A derivatives **5a**,**b** and **10a**,**b** were examined with respect to their cytotoxic activity towards human tumor and normal cell lines. For any compound with significant activity, cell cycle specificity was to be clarified as well as the question if DNA Top1 is indeed the biological target. 

#### 2.2.1. Evaluation of Cell Viability and Toxicity

**Figure 2:**
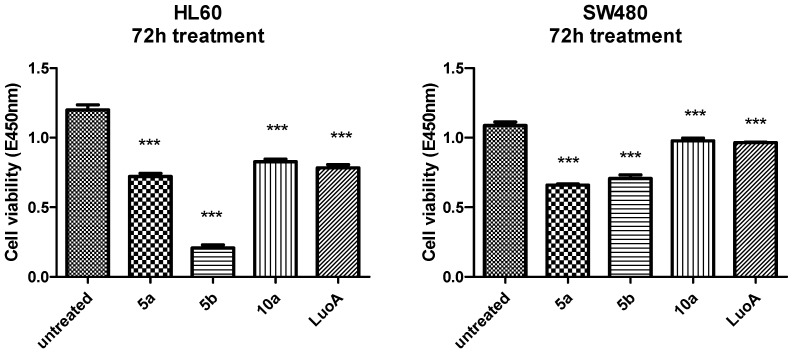
Cell viability screening; cells were treated with the indicated compounds at 40 µM, after 72 h of incubation the MTT assay was conducted, showing compound **5b** as significantly most potent in HL60 cells. Significance was designated as *** for *P* < 0.001. Error bars depict ± SD.

All four A-ring amino derivatives of Luotonin A were subjected to the MTT viability assay. Two standard cell lines were used: a human colon adenocarcinoma (SW480) and a human leukemia cell line (HL60). Because of insufficient medium solubility, compound **10b** had to be excluded. Out of the three remaining candidates, two compounds showed stronger activity than Luotonin A in solid tumor cells (SW480), whereas in leukemia cells (HL60), compound **5b** was found to be significantly more potent than its isomers (**5a** and **10a**) as well as the reference, Luotonin A (Figure 2). Thus, **5b** was selected for more in-depth investigations regarding its biological effects.

A dose-response relationship was examined for both Luotonin A and **5b** for the two examined cell lines. An IC_50_ of 7.17 ± 1.07 µM was calculated for **5b** in HL60 cells, whereas an IC_50_ for SW480 could not be determined due to solubility limitations, with 100 µM being the upper solubility limit. For both cell lines (HL60 and SW480), it was estimated that an IC_50_ of Luotonin A is not reached at 100 µM and 60 µM, respectively, which were the highest usable concentrations under the experiment conditions. 

These results of the MTT assay should be interpreted with some caution due to the observed enlarged morphology of treated cells, which might shift the photometric readout of the assay towards higher values, mistakenly indicating too high cell viability. Furthermore, the results of the FACS analysis (see below) show that most of the treated cancer cells—while still alive—are trapped in an irrecoverable state, which is triggered by relatively low concentrations of **5b**, compared to the concentrations indicated by the MTT assay (for further discussion, see Section 2.2.3).

**Figure 3:**
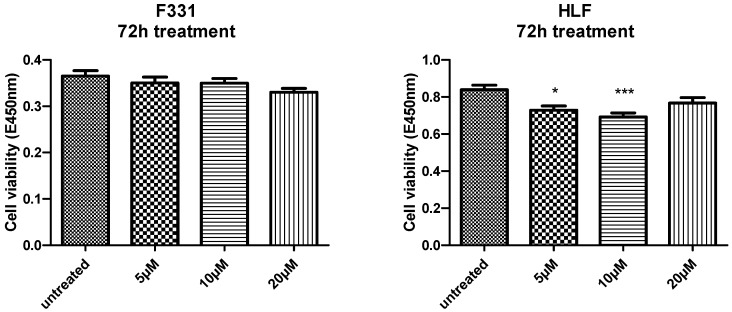
Toxicological profile of compound **5b** was examined on two fibroblast cell lines, as relative rapidly proliferating non-tumor human cells. Result of the MTT assay after exposure to **5b** for 72 h, no significant difference in cell growth in F331, some inhibitory activity could be statistically speculated for 10 µM at HLF, but not for 20 µM. Significance was designated as * for *P* < 0.05 and *** for *P* < 0.001. Error bars depict ± SD.

Toxicological viability assays, using two different fibroblast cell lines, including colon fibroblasts F331 and lung fibroblasts HLF, show low toxicity of the examined compound (Figure 3).

#### 2.2.2. Top1 Inhibition

**Figure 4:**
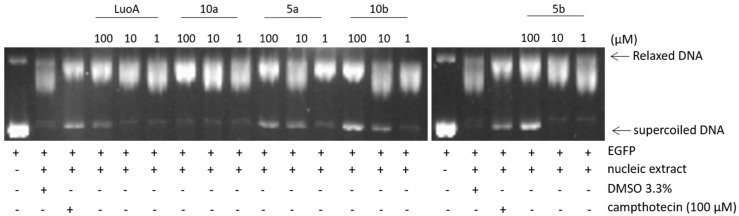
Effects of examined compounds on relaxation of supercoiled EGFP plasmid DNA by Top1. Three independent experiments were accomplished, showing concordant result. Top1-inhibitory activity was observed under exposure to **5a**, **5b** and **10b** (“+” and “−“ signs indicate the exact composition of the individual gel traces).

In order to evaluate the Top1 inhibition potential of our compounds, we performed DNA relaxation assays using Luotonin A and a known Topoisomerase I inhibitor, Camptothecin, as references. Supercoiled plasmid DNA EGFP was incubated with human Topoisomerase I and with the test compounds at a concentration range of 1 to 100 µM. Agarose gel electrophoresis was used to monitor the plasmid unwinding (Figure 4). These experiments revealed that the intensities of the lowest migrating band (corresponding to the supercoiled DNA) visibly increase with **5a**, **5b** and **10b** when compared to the original Luotonin A in a dose-dependent manner, partially indicating stronger activity than CPT. In contrast, **10a** showed no Topoisomerase I inhibition.

#### 2.2.3. Morphological Alterations and Cell Cycle Analysis: Induction of G2/M Arrest

**Figure 5:**
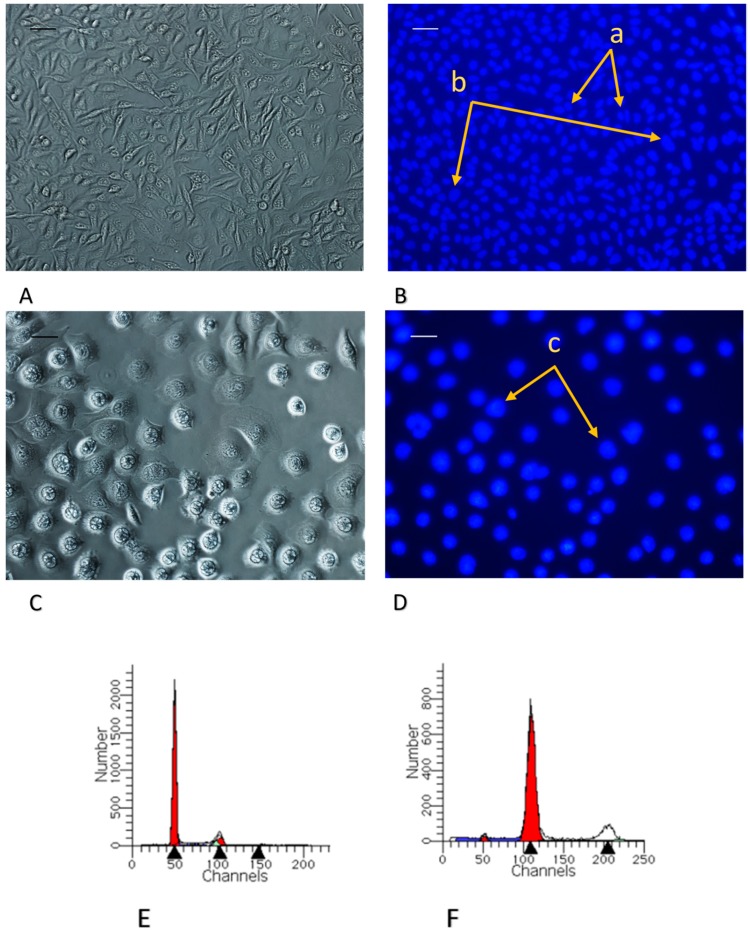
Results of nuclear staining with Hoechst 33285: SW480, untreated, 72 h incubation, phase contrast (**A**), UV excitation with blue/cyan filter (**B**): normal nucleus G1 (a), normal nucleus G2 (b); SW480, treated with **5b** (10 µM), 72 h incubation, phase contrast (**C**), UV excitation with blue/cyan filter (**D**): giant nucleus (c). The bars in the upper left corner of each panel represent a length of 25 µm. The subfigures E and F show representative histograms of control (**E**) and 10 µM **5b** exposed (**F**) SW480 cells. The 8n peak appears at an intensity of approx. 200.

In the course of our viability assay studies, we noticed some unexpected morphological alterations in both cell lines when treated with the investigated compounds, particularly in the case of compound **5b**. After incubation, cells treated with this compound show an enlarged and flattened morphology, involving uncommon nuclear changes that could be characterized as multi-nucleation and even macro-nucleation. This observation was analyzed in more detail by nuclear staining with the Hoechst 33285 dye [33], using adherent SW480 cells, as more convenient for this assay (see Figure 5). Neither clear apoptotic nor necrotic elements could be ascertained.

**Figure 6:**
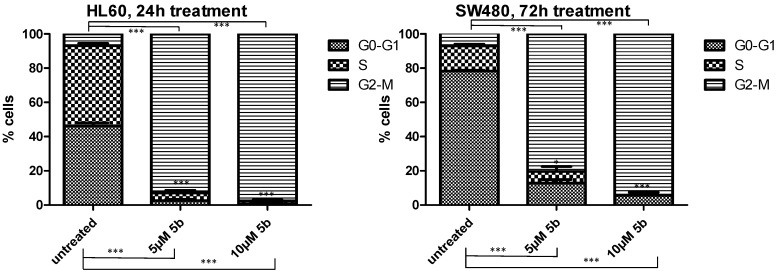
Cell cycle analysis of HL60 and SW480 cells treated with compound **5b**. A massive G2/M arrest was detected, showing at 10 µM almost no cell dividing. Significance was designated as * for *P* < 0.05 and *** for *P* < 0.001. Error bars depict ± SD.

**Figure 7:**
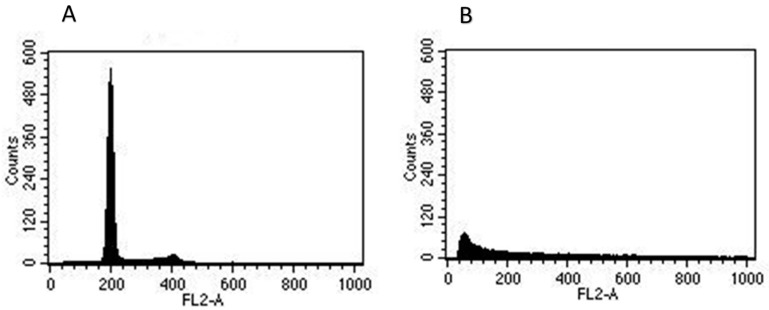
Results of the wash-out experiment: (**A**) SW480, non-treated, the scan shows a regular growing cell culture; (**B**) SW480, wash-out experiment, 48 h after removal of the examined compound; no recovery of cells, main peak in sub-G1 domain indicates cell death.

As the morphological changes indicated a G2 cell cycle arrest, we investigated the cell cycle state of treated cells using flow cytometry. Both cell lines (HL60 and SW480) demonstrated a strong cell cycle arrest in the G2 phase, when treated with **5b**, compared to untreated cells (see Figure 6). HL60 cells seem to respond faster, and the described effect could already be measured after 24 h of treatment. For SW480, 72 h were needed to obtain a comparable readout. Based on the results of the viability assays, the extent of the cell cycle block was surprising. A wash-out experiment that was conducted by substituting the treatment medium with a growth medium showed no recovery and massive loss of cells after incubation for further 48 h (Figure 7). This result demonstrates that the cells fragment even after removal of **5b**, indicating that the examined compound was indeed much more potent than the result of the MTT assay implies.

The G2 phase is a subphase of interphase, immediately followed by mitosis. Due to the particular sensitivity of the cell cycle during interphase, the G2 phase can be regarded as a very interesting target for diverse cytotoxic or cytostatic agents. If reparatory mechanisms fail to accomplish their work, a G2/M-arrest will be initiated and cell death pathways are induced. In their research, Bible et al., Tyagi et al., Ling et al. and Potter et al. demonstrated synergy of such cell-cycle checkpoint arrest with multiple chemotherapeutic agents, including irradiation [34,35,36,37]. G2/M-arrest-causing agents are therefore of interest as an alternative or subsidiary therapy option, in order to reduce the dosage and to mitigate side effects [35,38,39]. In view of the results obtained in the FACS analysis with **5b**, a potential use of this compound in combination with another cytostatic drug should be more closely investigated.

Morphological anomalies, induced by treatment with compound **5b**, combined with a massive G2/M arrest permit the assumption that some ‘mitotic catastrophe’ elements could also be considered as responsible for the observed cytotoxicity. A similar model was discussed by Kainz et al. in 2013, referring to the natural compound, 2-Deprenyl-Rheediaxanthone B [40]. Furthermore, mitotic catastrophe following a G2/M arrest was described recently by Gu and coworkers: cells that initiate a G2/M arrest often miscarry mitosis and execute mitotic catastrophe, which results in the formation of enlarged cells usually bearing tetraploid DNA content [41]. However, this assumption requires further investigation.

#### 2.2.4. Stimulation of Caspase Activity by Compound **5b**

**Figure 8:**
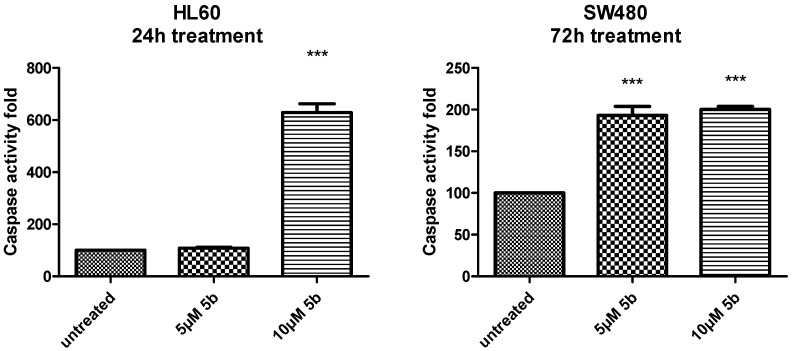
Caspase assay of HL60 and SW480 cells after treatment. Particularly strong activation of caspases was observed in HL60 cells treated with 10 µM of compound **5b**. Significance was designated as *** for *P* < 0.001. Error bars depict ± SD.

To determine if caspase activation is also involved in the observed cell death pathway, an assessment of caspase activity was conducted, following the protocol of Werner et al. [42]. In both cell lines, an increase of caspase activity was detected. For SW480 cells, the increase of caspase activity under treatment with **5b** was not as strong as for HL60 cells. However, it was already observed with the 5 µM concentration. In the HL60 cultures, 5 µM **5b** did not affect caspase activity, but a 6-fold increase at the 10 µM concentration was observed (Figure 8). 

We assume that some metabolic and genetic differences between the investigated cell lines could be the cause for such a difference in caspase activation. Furthermore, confirmation of caspase activity, based on this result, implies that cells treated with **5b** could terminate in apoptotic cell death. 

Caspases are also crucial for termination of mitotic death, which implicates that involvement of the above-mentioned mitotic catastrophe elements may result in an activation of apoptotic elements. Ho et al. suggest that apoptotic and mitotic death pathways may occur simultaneously, following a G2/M arrest [43,44,45]. Based on these results, we believe that in our experiment, mitotic catastrophe might be associated with apoptosis, or at least end up in an apoptosis-related cell execution. For this assumption, some confirmations would be needed.

## 3. Conclusions

In this work, four novel A-ring amino derivatives of the alkaloid Luotonin A were synthesized via two orthogonal pathways involving intramolecular cycloaddition reactions as the key step. The target compounds were obtained in good yields by mild catalytic transfer hydrogenation of the corresponding nitro precursors. In the biological evaluation, we detected at least one compound that showed convincingly stronger antiproliferative activity than the lead compound, Luotonin A. Similar to CPT, three of four examined compounds were clearly superior to Luotonin A, inhibiting the Top1 enzyme. Surprisingly, the most active compound in the viability assay, **5b**, showed no activity of Top1 inhibition in a DNA relaxation assay at lower antiproliferative concentrations. Furthermore, under exposure to compound **5b** in such concentrations, we observed a massive G2/M arrest, as well as elements of both mitotic catastrophe and apoptosis. This behavior is, although under certain conditions observed in some other cases [46,47,48], not typical for CPT-related Topoisomerase I inhibitors, which usually exhibit an S phase arrest rather than a G2/M arrest. Therefore, some other biological targets for our compound might be relevant, or another effect on the enzyme could be speculated. Considering the detected G2/M arrest of tumor cells under exposure to **5b**, we suggest the observed G2 checkpoint inhibition to be a mitotic disruptor that leads either to apoptotic or (also possible) mitotic cell death. 

Hence, we hope that our work might contribute to complementing the efforts of developing new therapy approaches in which using one G2/M-arrest-inducing agent and/or mitotic cell death initiator could eventually lead to some alternative therapy options and milden the side effects of conventional therapy. 

## 4. Experimental Section

### 4.1. Chemistry

#### 4.1.1. General

Melting points (uncorrected) were determined on a Kofler hot-stage microscope (Leica GmbH, Wetzlar, Germany). ^1^H-NMR and ^13^C-NMR spectra (see Supplementary Materials) were recorded on a Bruker Avance III 400 spectrometer (Bruker BioSpin GmbH, Rheinstetten, Germany) at 400 MHz and 100 MHz, respectively; chemical shifts (ppm) were referenced to residual amounts of undeuterated solvents. Mass spectra (EI) were obtained on a Shimadzu QP5050A DI 50 instrument (Shimadzu Corp., Kyoto, Japan); high-resolution mass spectra (ESI-TOF) were recorded on a Bruker maXis HD spectrometer (Bruker Daltonics GmbH, Bremen, Germany). Column chromatography was carried out on Merck Kieselgel 60, 0.063–0.200 mm. Thin layer chromatography was done on Merck aluminium sheets pre-coated with Kieselgel 60 F_254_ (Merck, Darmstadt, Germany). Microanalyses were performed at the Microanalytical Laboratory, Faculty of Chemistry, University of Vienna. Ethyl 4-oxo-3,4-dihydroquinazoline-2-carboxylate (**1**) was prepared according to a literature procedure [23]. 

#### 4.1.2. Syntheses

*N-(2-Nitrophenyl)-4-oxo-3,4-dihydroquinazoline-2-carboxamide* (**2b**). To a solution of 2-nitroaniline (1105 mg, 8 mmol) in dry 1,2-dichloroethane (20 mL) under argon was added dropwise a 2 M solution of AlMe_3_ (4.0 mL, 8 mmol) in heptane. The mixture was stirred for 30 min at room temperature, then the ester **1** (1091 mg, 5 mmol) was added in one portion, and the mixture was refluxed for 2 h. After cooling to 0 °C, it was then quenched by slow addition of 2 N HCl (20 mL), followed by water (80 mL). The resulting liquid/liquid/solid system was filtered and the filter cake was washed with 70% EtOH and dried to give compound **2b** (903 mg, 58%) in sufficient purity for the next reaction step. Analytically pure material can be obtained by recrystallization from EtOH as pale yellow crystals, m.p. > 315 °C. MS (EI, 70 eV): *m*/*z* = 310 (M^+^, 25%), 264 (84), 146 (78), 145 (22), 119 (100), 118 (21), 91 (18), 90 (49); ^1^H-NMR (DMSO-*d*_6_) δ: 12.76 (br s, 1H, 3-H), 12.15 (br s, 1H, amide NH), 8.54 (d, *J* = 8.4 Hz, 1H, phenyl 6′-H), 8.26 (dd, *J* = 8.4 Hz, 1.5 Hz, 1H, phenyl 3′-H), 8.22 (d, *J* = 7.8 Hz, 1H, 5-H), 7.98–7.93 (m, 1H, 7-H), 7.89–7.85 (m, 2H, 8-H, phenyl 5′-H), 7.68 (t, *J* = 7.8 Hz, 1H, 6-H), 7.46–7.42 (m, 1H, phenyl 4′-H); ^13^C-NMR (DMSO-*d*_6_) δ: 161.0, 158.0, 146.4, 145.1, 138.7, 135.7, 135.1, 132.3, 128.7, 127.9, 126.3, 125.9, 125.1, 122.9, 122.7. Anal. calcd. for C_15_H_10_N_4_O_4_ · 0.1 H_2_O: C, 57.73; H, 3.29; N, 17.95. Found: 57.87; H, 2.96; N, 17.56. HRMS (ESI-TOF) calcd. for C_15_H_11_N_4_O_4_ ([M + H]^+^): 311.0775. Found: 311.0775.

*N-(2-Nitrophenyl)-4-oxo-3-(prop-2-yn-1-yl)-3,4-dihydroquinazoline-2-carboxamide* (**3b**). In a round- bottomed flask, finely ground anilide **2b** (155 mg, 0.5 mmol) was added to DMSO (20 mL) and the resulting suspension was stirred at room temperature for 30 min. Then, finely powdered KOH (34 mg, 0.5 mmol) and tetrabutylammonium bromide (10 mg) were added and the mixture was sonicated in an ultrasound cleaning bath for 10 min. While the mixture was vigorously stirred, a solution of propargyl bromide (80% in toluene; 82 mg, 0.5 mmol) in DMSO (10 mL) was dropwise added over a period of 2 h. Stirring was continued at room temperature for 24 h (TLC monitoring: CH_2_Cl_2_/ethyl acetate, 19:1), then the solution was poured into water (100 mL) and the precipitate was collected by filtration, washed with water, and dried. This material was subjected to column chromatography (eluent: CH_2_Cl_2_/ethyl acetate, 19:1). Evaporation of the main fraction, followed by recrystallization from ethyl acetate/light petroleum afforded compound **3b** (133 mg, 76%) as yellow crystals, m.p. 207–208 °C. MS (EI, 70 eV): *m*/*z* = 348 (M^+^, 16%), 347 (39), 331 (30), 330 (25), 303 (49), 302 (100), 300 (42), 273 (26), 156 (37), 155 (49), 145 (75), 130 (39), 129 (61), 119 (58), 102 (37), 90 (78); ); ^1^H-NMR (CDCl_3_) δ: 12.72 (br s, 1H, NH), 8.92 (dd, *J* = 8.5 Hz, 1.2 Hz, 1H, phenyl 6′-H), 8.41–8.35 (m, 1H, 5-H), 8.32 (dd, *J* = 8.4 Hz, 1.5 Hz, 1H, phenyl 3′-H), 7.95–7.90 (m, 1H, 8-H), 7.87 (ddd, *J* = 8.2 Hz, 7.0 Hz, 1.5 Hz, 1H, 7-H), 7.79–7.70 (m, 1H, phenyl 5′-H), 7.64 (ddd, *J* = 8.2 Hz, 7.0 Hz, 1.4 Hz, 1H, 6-H), 7.31 (ddd, *J* = 8.5 Hz, 7.3 Hz, 1.3 Hz, 1H, phenyl 4′-H), 5.59 (d, *J* = 2.5 Hz, 2H, CH_2_), 2.27 (t, *J* = 2.5 Hz, 1H, acetylene H); ^13^C-NMR (CDCl_3_) δ: 161.4, 159.4, 144.9, 144.5, 137.3, 136.1, 135.3, 133.8, 129.6, 128.6, 127.6, 126.2, 124.4, 122.1, 121.9, 78.9, 72.2, 33.7. Anal. calcd. for C_18_H_12_N_4_O_4_: C, 62.07; H, 3.47; N, 16.08. Found: C, 61.81; H, 3.13; N, 15.71.

*4-Nitroquinolino[2′,3′:3,4]pyrrolo[2,1-b]quinazolin-11(13H)-one* (**4b**). To a solution of triphenyl-phosphine oxide (835 mg, 3 mmol) in dry CH_2_Cl_2_ (22 mL) was dropwise added trifluoro-methanesulfonic anhydride (0.25 mL, 1.5 mmol) at 0 °C under argon, and the mixture was stirred at the same temperature for 15 min. Then, the educt **3b** (348 mg, 1 mmol) was added in one portion at 0 °C, and the mixture was stirred for 24 h while slowly warming to room temperature. The mixture was evaporated under reduced pressure and the solid residue was re-suspended in 10% aq. NaHCO_3_ solution and stirred for 1 h. The product was collected by filtration and washed consecutively with water, EtOH and cold CHCl_3_. Recrystallization from CHCl_3_ afforded **4b** (211 mg, 64%) as pale yellow crystals, m.p. 263–264 °C. MS (EI, 70 eV): *m*/*z* = 330 (M^+^, 12%), 300 (10), 284 (6), 81 (8), 69 (100), 65 (39), 64 (15), 57 (9); ^1^H-NMR (DMSO-*d*_6_) δ: 8.99 (s, 1H, 14-H),8.49 (d, *J* = 8.7 Hz, 1H, 1-H),8.43 (d, *J* = 7.7 Hz, 1H, 3-H), 8.32 (d, *J* = 7.6 Hz, 1H, 10-H), 8.03 (d, *J* = 8.3 Hz, 1H, 7-H), 8.00–7.85 (m, 2H, 2-H, 8-H), 7.67 (t, *J* = 7.4 Hz, 1H, 9-H), 5.36 (s, 2H, 13-CH_2_); ^13^C-NMR (DMSO-*d*_6_) δ: 159.6, 153.6, 152.3, 148.8, 148.4, 139.0, 134.6, 133.1, 132.5, 132.3, 129.0, 128.2, 127.6, 127.4, 125.9, 124.0, 121.2, 47.6. HRMS (ESI-TOF) calcd. for C_18_H_11_N_4_O_3_ ([M + H]^+^): 331.0826. Found: 331.0824.

*2-Aminoquinolino[2′,3′:3,4]pyrrolo[2,1-b]quinazolin-11(13H)-one* (**5a**). To a suspension of the nitro compound **4a** [16] (165 mg, 0.503 mmol) in EtOH (50 mL) were added hydrazine hydrate (350 mg, 7 mmol) and 10% palladium on carbon (30 mg) and the mixture was refluxed under an argon atmosphere for 2 h (TLC monitoring: CH_2_Cl_2_/EtOH, 30:1). The solvent was evaporated under reduced pressure (max. bath temperature: 25 °C) and the residue was taken up in DMF (15 mL). The catalyst was removed by filtration and the filtrate was concentrated to approx. 5 mL under reduced pressure, then it was diluted with water (5 mL). The precipitate was collected by filtration and washed with 70% EtOH to give the amino compound **5a** (120 mg, 80%) as yellow crystals, m.p. >350 °C (decomp.). MS (EI, 70 eV): *m*/*z* = 300 (M^+^, 13%), 231 (100), 71 (57), 70 (79), 69 (44), 57 (93), 55 (42), 44 (39), 43 (88); for ^1^H-NMR and ^13^C-NMR data, see Table 1. HRMS (ESI-TOF) calcd. for C_18_H_13_N_4_O ([M + H]^+^): 301.1084. Found: 301.1083.

*4-Aminoquinolino[2′,3′:3,4]pyrrolo[2,1-b]quinazolin-11(13H)-one* (**5b**). To a suspension of the nitro compound **4b** (165 mg, 0.503 mmol) in EtOH (50 mL) were added hydrazine hydrate (350 mg, 7 mmol) and 10% palladium on carbon (30 mg) and the mixture was refluxed under an argon atmosphere for 2 h (TLC monitoring: CH_2_Cl_2_/EtOH, 30:1). The solution was filtered while hot and then cooled to room temperature. The red crystals were collected by filtration and washed with cold EtOH. The filtrate was evaporated to dryness under reduced pressure and the residue was dissolved in DMF (15 mL). The solution was filtered twice to remove traces of colloidal Pd/C, then it was diluted with water (20 mL). The red precipitate was collected by filtration and washed with cold EtOH. The combined crops were recrystallized from CHCl_3_ to afford **5b** (107 mg, 71%) as red crystals, m.p. >315 °C (decomp.). MS (EI, 70 eV): *m*/*z* = 300 (M^+^, 44%), 215 (18), 204 (18), 203 (40), 178 (81), 145 (24), 57 (100), 41 (25); for ^1^H-NMR and ^13^C-NMR data, see Table 1. HRMS (ESI-TOF) calcd. for C_18_H_13_N_4_O ([M + H]^+^): 301.1084. Found: 301.1081.

*3-[3-(2-Nitrophenyl)prop-2-yn-1-yl]-4-oxo-3,4-dihydroquinazoline-2-carboxamide* (**7a**). To a stirred suspension of the amide **6** [29] (454 mg, 2 mmol) in dry CH_2_Cl_2_ (30 mL) was added 2-iodonitrobenzene (746 mg, 3 mmol), 2,6-di-tert-butyl-4-methylphenol (110 mg, 0.5 mmol), CuI (76 mg, 0.4 mmol), Pd(PPh_3_)Cl_2_ (140 mg, 0.2 mmol) and triethylamine (486 mg, 4.8 mmol). The mixture was flushed with argon and stirred in a closed vessel at room temperature for 2 h (TLC monitoring: CH_2_Cl_2_/ethyl acetate, 2:1). It was then diluted with CH_2_Cl_2_ (80 mL) and filtered. The filtrate was concentrated under reduced pressure and the residue was subjected to column chromatography (eluent: CH_2_Cl_2_/ethyl acetate, 2:1). Evaporation of the main fraction, followed by recrystallization from toluene/ethyl acetate and subsequently from EtOH gave the product **7a** (328 mg, 48%) as light-brown crystals, m.p. 193–194 °C. MS (EI, 70 eV): *m*/*z* = 349 ([M + 1]^+^, 2%), 331 (36), 288 (88), 146 (100), 145 (86), 130 (96), 119 (37), 102 (66), 90 (43), 76 (64), 63 (33); ^1^H-NMR (DMSO-*d*_6_) δ: 8.52 (br s, 1H, NH), 8.23 (dd, *J* = 8.2 Hz, 1.4 Hz, 1H, 5-H), 8.22 (br s, 1H, NH), 8.09 (d, *J* = 8.2 Hz, 1H, phenyl 3′-H), 7.93 (td, *J* = 7.5 Hz, 1.5 Hz, 1H, 7-H, shows positive NOE on irradiation at 7.78 ppm), 7.78 (d, *J* = 7.8 Hz, 1H, 8-H, shows positive NOE on irradiation at 7.93 ppm), 7.73–7.69 (m, 2H, phenyl 5′-H, 6′-H), 7.68–7.60 (m, 2H, 6-H, phenyl 4′-H, shows positive NOE on irradiation at 8.09 ppm or at 7.93 ppm), 5.37 (s, 2H, CH_2_); ^13^C-NMR (DMSO-*d*_6_) δ: 163.2, 160.0, 149.3, 148.6, 145.8, 135.2, 134.8, 133.6, 130.0, 128.4, 127.6, 126.6, 124.6, 120.9, 116.3, 92.0, 78.3, 33.9. Anal. calcd. for C_18_H_12_N_4_O_4_ · 0.3 H_2_O: C, 61.12; H, 3.59; N, 15.84. Found: C, 61.11; H, 3.24; N, 15.73.

*3-[3-(4-Nitrophenyl)prop-2-yn-1-yl]-4-oxo-3,4-dihydroquinazoline-2-carboxamide* (**7b**). A solution/ suspension of the amide **6** [29] (227 mg, 1 mmol) in dry DMF (1 mL) was diluted with dry CH_2_Cl_2_ (15 mL). Then, 4-iodonitrobenzene (373 mg, 1.5 mmol), 2,6-di-tert-butyl-4-methylphenol (55 mg, 0.25 mmol), CuI (38 mg, 0.2 mmol), Pd(PPh_3_)Cl_2_ (70 mg, 0.1 mmol) and triethylamine (243 mg, 2.4 mmol) were added consecutively. The mixture was flushed with argon and stirred in a closed vessel at room temperature for 2 h (TLC monitoring: CH_2_Cl_2_/ethyl acetate, 2:1). It was then diluted with CH_2_Cl_2_ (40 mL) and filtered. The filtrate was concentrated under reduced pressure and the residue was subjected to column chromatography (eluent: CH_2_Cl_2_/ethyl acetate, 2:1). Evaporation of the main fraction, followed by recrystallization from toluene/ethyl acetate and subsequently from EtOH gave the product **7b** (188 mg, 54%) as colorless needles, m.p. 224–225 °C. MS (EI, 70 eV): *m*/*z* = 348 (M^+^, 100%), 305 (16), 258 (27), 229 (22), 198 (14), 175 (32), 160 (14), 146 (38), 130 (40), 129 (39), 114 (35), 102 (50), 76 (38), 63 (51); ^1^H-NMR (DMSO-*d*_6_) δ: 8.58 (br s, 1H, NH), 8.26–8.18 (m, 4H, NH, 5-H, phenyl 3′-H, 5′-H), 7.95–7.90 (m, 1H, 7-H), 7.77 (d, *J* = 9.0 Hz, 1H, 8-H), 7.70–7.63 (m, 3H, 6-H, phenyl 2′-H, 6′-H), 5.34 (s, 2H,CH_2_); ^13^C-NMR (DMSO-*d*_6_) δ: 163.2, 160.0, 148.7, 147.0, 145.8, 135.2, 132.7, 128.4, 128.3, 127.6, 126.5, 123.7, 120.9, 89.9, 81.4, 33.8. Anal. calcd. for C_18_H_12_N_4_O_4_: C, 62.07; H, 3.47; N, 16.08. Found: C, 61.74; H, 3.25; N, 15.73.

*3-[3-(2-Nitrophenyl)prop-2-yn-1-yl]-4-oxo-3,4-dihydroquinazoline-2-carbonitrile* (**8a**). In an oven- dried 50 mL round-bottomed flask, the amide **7a** (100 mg, 0.287 mmol) was dissolved in dry CHCl_3_ (10 mL). To this solution was added freshly distilled POCl_3_ (1 mL) and the mixture was heated to 90 °C under exclusion of moisture for 24 h (TLC monitoring: CH_2_Cl_2_/EtOH, 30:1). The dark suspension was dropwise added to ice-water (50 mL) with stirring. After 30 min, the phases were separated and the aqueous layer was extracted with CHCl_3_ (3 × 15 mL). The combined organic layers were washed with water and brine, dried over Na_2_SO_4_ and concentrated under reduced pressure. The residue was purified by column chromatography (eluent: CH_2_Cl_2_), followed by recrystallization from EtOH to afford the nitrile **8a** (68 mg, 72%) as colorless crystals, m.p. 170–172 °C. MS (EI, 70 eV): *m*/*z* = 330 (M^+^, 5%), 313 (65), 160 (48), 154 (70), 146 (61), 130 (46), 104 (100), 102 (69), 89 (59), 76 (89), 63 (50); ^1^H-NMR (CDCl_3_) δ: 8.37 (dd, *J* = 8.0, 1.1 Hz, 1H, 5-H), 8.07 (dd, *J* = 8.3, 1.1 Hz, 1H, phenyl 3′-H, shows positive NOE on irradiation at 7.49 ppm), 7.90–7.82 (m, 2H, 7-H, 8-H), 7.69–7.64 (m, 2H, 6-H, phenyl 6′-H, shows positive NOE on irradiation at 8.37 ppm), 7.58 (td, *J* = 7.7, 1.3 Hz, 1H, phenyl 4′-H), 7.49 (td, *J* = 7.7, 1.5 Hz, 1H, phenyl 5’-H, shows positive NOE on irradiation at 8.07 ppm), 5.37 (s, 2H, CH_2_); ^13^C-NMR (CDCl_3_) δ: 159.3, 146.4, 135.6, 135.2, 133.1, 130.8, 130.5, 129.6, 128.9, 127.6, 124.9, 122.8, 117.3, 111.4, 89.1, 81.3, 36.0. Anal. calcd. for C_18_H_10_N_4_O_3_: C, 65.45M H, 3.05; N, 16.96. Found: C, 65.05; H, 2.92; N, 16.81.

*3-[3-(4-Nitrophenyl)prop-2-yn-1-yl]-4-oxo-3,4-dihydroquinazoline-2-carbonitrile* (**8b**). This compound was prepared and isolated as described for compound **8a**, starting from the amide **7b** (100 mg, 0.287 mmol). Recrystallization from EtOH gave the nitrile **8b** (88 mg, 90%) as colorless needles, m.p. 188–190 °C. MS (EI, 70 eV): *m*/*z* = 330 (M^+^, 100%), 284 (45), 256 (21), 230 (7), 208 (12), 160 (79), 130 (27), 114 (73), 113 (75), 102 (80), 88 (38), 76 (53), 63 (92), 50 (36); ^1^H-NMR (CDCl_3_) δ: 8.39–8.36 (m, 1H, 5-H), 8.20–8.16 (m, 2H, phenyl 3′-H, 5′-H), 7.93–7.87 (m, 1H, 7-H), 7.86–7.82 (m, 1H, 8-H), 7.72–7.67 (m, 1H, 6-H), 7.64–7.69 (m, 2H, phenyl 2′-H, 6′-H), 5.34 (s, 2H, CH_2_); ^13^C-NMR (CDCl_3_) δ: 159.3, 147.9, 146.4, 135.7, 133.1, 130.7, 130.6, 129.0, 128.5, 127.6, 123.7, 122.8, 111.4, 86.4, 83.9, 35.8. Anal. calcd. for C_18_H_10_N_4_O_3_ · 0.5 H_2_O: C, 63.72; H, 3.27; N, 16.51. Found: C, 63.96; H, 3.01; N, 16.20.

*1-Nitroquinolino[2’,3’:3,4]pyrrolo[2,1-b]quinazolin-11(13H)-one* (**9a**). To a solution of the nitrile **8a** (100 mg, 0.303 mmol) in 1,2-dichlorobenzene (8 mL) was added a 0.1 M solution of DBU in 1,2-dichlorobenzene (0.15 mL, 5 mol%). After flushing with argon, the vessel was closed and the mixture was heated to 110–120 °C for 24 h. If TLC (CH_2_Cl_2_/EtOH, 30:1) indicated incomplete conversion, another portion of the base (0.15 mL of 0.1 M DBU solution) was added and heating was continued for another 24 h. The resulting suspension was chilled and diluted with Et_2_O (55 mL). The precipitate was collected by filtration, washed with Et_2_O and recrystallized from CHCl_3_ to afford compound **9a** (46 mg, 46%) as almost colorless crystals, m.p. 325–327 °C. MS (EI, 70 eV): *m*/*z* = 330 (M^+^, 100%), 284 (47), 262 (31), 183 (33), 108 (28), 77 (36), 69 (31), 57 (51), 55 (36), 43 (47); ^1^H-NMR (DMSO-*d*_6_) δ: 9.24 (s, 1H, 14-H), 8.66 (d, *J* = 8.5 Hz, 1H, 4-H), 8.58 (d, *J* = 7.4 Hz, 1H, 2-H), 8.27 (d, *J* = 8.0 Hz, 1H, 10-H), 8.08 (t, *J* = 8.1 Hz, 1H, 3-H), 7.98–7.91 (m, 2H, 7-H, 8-H), 7.66–7.61 (m, 1H, 9-H), 5.36 (s, 2H, 13-CH_2_); ^13^C-NMR (DMSO-*d*_6_) δ: 159.6, 153.2, 152.5, 148.9, 145.9, 145.4, 139.7, 136.6, 134.7, 134.0, 129.1, 128.2, 127.6, 126.2, 126.0, 121.3, 120.7, 48.1. HRMS (ESI-TOF) calcd. for C_18_H_11_N_4_O_3_ ([M + H]^+^): 331.0826. Found: 331.0818.

*3-Nitroquinolino[2’,3’:3,4]pyrrolo[2,1-b]quinazolin-11(13H)-one* (**9b**). This compound was pre-pared as described for compound **9a**, starting from the nitrile **8b** (100 mg, 0.303 mmol). Recrystallization from DMF/CHCl_3_ afforded **9b** (57 mg, 57%) as yellow crystals, m.p. > 310 °C (decomp.). MS (EI, 70 eV): *m*/*z* = 330 (M^+^, 100%), 300 (14), 284 (59), 254 (14), 228 (10), 142 (17), 114 (13), 77 (15), 76 (20), 63 (25), 50 (18); ^1^H-NMR (DMSO-*d*_6_) δ: 9.06 (s, 1H, 4-H), 8.96 (s, 1H, 14-H, shows positive NOE on irradiation at 5.37 ppm ), 8.47–8.45 (m, 2H, 1-H, 2-H), 8.30 (d, *J* = 8.0 Hz, 1H, 10-H), 7.98–7.95 (m, 2H, 7-H, 8-H), 7.68–7.64 (m, 1H, 9-H), 5.37 (s, 2H, 13-CH_2_); ^13^C-NMR (DMSO-*d*_6_) δ: 160.2, 159.6, 154.3, 152.4, 151.5, 148.9, 148.3, 147.0, 134.7, 134.2, 132.2, 131.6, 128.2, 126.0, 125.4, 121.3, 121.2, 47.8. HRMS (ESI-TOF) calcd. for C_18_H_11_N_4_O_3_ ([M + H]^+^): 331.0826. Found: 331.0827.

*1-Aminoquinolino[2’,3’:3,4]pyrrolo[2,1-b]quinazolin-11(13H)-one* (**10a**). To a suspension of the nitro compound **9a** (100 mg, 0.303 mmol) in EtOH (20 mL) was added hydrazine hydrate (240 mg, 4.8 mmol) and 10% palladium on carbon (10 mg) and the mixture was refluxed under an argon atmosphere for 1 h (TLC monitoring: CH_2_Cl_2_/EtOH, 30:1). The mixture was diluted with EtOH (300 mL), refluxed for a few minutes and filtered hot to remove the catalyst. The filter cake was re-suspended in EtOH (300 mL), refluxed for a few minutes and again filtered while still hot. This procedure was repeated until the last filtrate did not show any fluorescence under UV_366_ light. The combined filtrates were evaporated under reduced pressure to afford the amino compound **10a** (89 mg, 98%) as reddish crystals, m.p. 315–317 °C (decomp.). MS (EI, 70 eV): *m*/*z* = 300 (M^+^, 100%), 272 (14), 150 (13), 111 (16), 97 (22), 83 (21), 77 (19), 71 (27), 57 (41), 43 (26); for ^1^H-NMR and ^13^C-NMR data, see Table 1. HRMS (ESI-TOF) calcd. for C_18_H_13_N_4_O ([M + H]^+^): 301.1084. Found: 301.1075.

*3-Aminoquinolino[2’,3’:3,4]pyrrolo[2,1-b]quinazolin-11(13H)-one* (**10b**). This compound was prepared and isolated as described for compound **10a**, starting from the nitro compound **9b** (100 mg, 0.303 mmol). Evaporation of the combined ethanolic filtrates afforded **10b** (88 mg, 97%) as yellow crystals, m.p. > 310 °C (decomp.). MS (EI, 70 eV): *m*/*z* = 300 (M^+^, 100%), 272 (9), 244 (5), 223 (9), 150 (11), 122 (7), 102 (7), 77 (16), 64 (10), 63 (11), 51 (9); for ^1^H-NMR and ^13^C-NMR data, see Table 1. HRMS (ESI-TOF) calcd. for C_18_H_13_N_4_O ([M + H]^+^): 301.1084. Found: 301.1084.

### 4.2. Biology

#### 4.2.1. Cell Culture

SW480 (ATCC® CCl-228^TM^) as representative for solid tumors, and HL60 (ATCC® CCl-240^TM^) for non-solid tumors as well as human primary lung fibroblasts, normal, HLF (ATCC® PCS-201-013™), were obtained from the American Type Culture Collection (ATCC®). Colon-associated fibroblasts F331 have been isolated from human fetal colon [49].

All cell lines were kept under standard tissue culture conditions and passaged when dense. Media used were Minimal Essential Medium (MEM) for SW480; RPMI-1640 for HL60 and Dulbecco’s medium for fibroblasts. The media were supplemented with 10% fetal calf serum. 

#### 4.2.2. Cell Viability Assay

Cells were seeded at a density of 5 × 10^4^ cells per mL (SW480) and 10^5^ cells per mL (HL60) in a 96-well plate. Compounds were diluted from DMSO stock solutions into MEM-BSA or serum-free RPMI-1640 medium to defined concentrations and added to the cells. Concentration of DMSO was limited with 0.6%, because higher DMSO concentration had influence on cell growth. Treated cells were incubated for 72 h at 37 °C and 5% CO_2_. After incubation, MTT viability assay was performed using EZ4U® Cell proliferation & Cytotoxicity Assay (Biomedica). For photometric evaluation, a plate reader TECAN Infinite® 200 PRO was used. IC_50_ values were determined as follows: HL60 cells were exposed to the test compound **5b** at a concentration range of 1 µM to 100 µM for 72 h and cell viability was measured using the MTT assay. The data from three independent experiments were pooled and IC_50_ was calculated using a nonlinear regression model of GraphPad Prism (see Section 4.2.7).

#### 4.2.3. DNA Relaxation Assay

The relaxation of the supercoiled EGFP plasmid DNA by the nucleic extract from MCF-7 cells was determined as previously described [50]. Concisely, plasmid DNA (EGFP) was incubated for 30 min at 37 °C with decreasing concentrations of the test compound dissolved in DMSO in a final volume of 30 μL nucleic extract (1:55), 10 mM Tris (pH 7.9), 100 mM KCl, 10 mM MgCl2, 0.5 mM dithiothreitol (DTT), 0.5 mM EDTA, and 0.03 mg/mL bovine serum albumin (BSA). Camptothecin, dissolved in DMSO, was used as positive control. The reaction was stopped by incubation with 5% SDS containing 1 mg/mL proteinase K at 37 °C for 30 min. Subsequently, samples were separated by submarine 1% agarose gel electrophoresis (55 V, 2 h) and gels were stained with 10 μL/100 mL ethidium bromide for 30 min. UV-transilluminated gels (Gel Doc™ XR, BioRad) were documented by Quantity One analysis software. 

#### 4.2.4. Cell Cycle Distribution Assay (FACS) 

HL60 cells were seeded at a density of 5 × 10^5^ cells per mL in a suspension culture flask. SW480 cells were seeded at a density of 3 × 10^5^ cells per mL in a 6 cm petri dish. 24 h later, compounds were added in defined concentration diluted into serum-free media. Incubation was done for 24 h for HL60 and 72 h for SW480 cultures. After this, the cells were harvested and nuclei isolated and stained with propidium iodide as described before [40]. Cell cycle distribution was evaluated using BD science FACSCalibur®.

#### 4.2.5. Caspase Assay

For determination of caspase activity under influence of experimental substance, the cells were seeded at appropriate density incubated with the compounds for 24 h (for HL60) or 72 h (for SW480). Cell proteins were then isolated and prepared for caspase assay. Caspase reaction solution was prepared fresh and added to protein lysate. After 90 min of incubation at 37 °C under light excision, TECAN Infinite® 200 PRO plate reader was used to measure a caspase activity at 405Ex/535Em.

#### 4.2.6. Nuclear Staining

SW480 cells were cultured in 24-well plates at a density of 10^5^ cells per mL for 24 h. After 72 h of treatment, the cells were washed, fixed in ice-cold acetone-methanol (1:1), then washed again with PBS and stained with Hoechst 33285, incubating cells for 15 min in dark at room temperature. Photos were captured with a fluorescence microscope equipped with UV light source and a blue/cyan filter.

#### 4.2.7. Statistics

Statistical analysis was performed using GraphPad Prism 5.00, GraphPad Software, California, USA. Conducted was one-way analysis of variance (ANOVA) with Dunnett’s post or Bonferroni test and two-way ANOVA, as appropriate. Significance was designated as * for *P* < 0.05, ** for *P* < 0.01, and *** for *P* < 0.001. Error bars depict ± SD.

## Figures and Tables

**Table 1:**
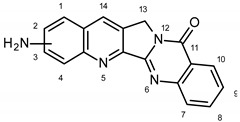
^1^H-NMR (400 MHz) and ^13^C-NMR (100 MHz) signals of the amino-substituted Luotonin A derivatives **5a**,**b** and **10a**,**b** (DMSO-*d*_6_ solutions, chemical shifts in ppm, coupling constants in Hz).

Signal	10a (1-amino)	5a (2-amino)	10b (3-amino)	5b (4-amino)
1-H	–	6.91 (d, *J* = 2.3)	7.81 (d, *J* = 8.0)	7.22 (dd, *J* = 8.2, 1.0)
1-C	149.7	104.1	128.9	113.8
2-H	6.86 (dd, *J* = 7.6)	–	7.16 (dd, *J* = 8.8, 2.0)	7.45 (t, *J* = 7.8)
2-C	108.8	149.0	120-9	129.7
3-H	7.57 (t, *J* = 8.1)	7.31 (dd, *J* = 9.1, 2.4)	–	6.98 (dd, *J* = 7.6, 1.1)
3-C	131.3	123.0	151.1 ^a^	109.1
4-H	7.43 (d, *J* = 8.2)	7.93 (d, *J* = 9.3)	7.12 (d, unresolved)	–
4-C	116.7	130.7	105.9	146.2
4a-C	145.7	142.8	151.0 ^a^	138.1
5a-C	150.7	145.5	150.6	147.9
5b-C	153.4	153.6	153.7	153.2
6a-C	149.1	149.3	149.2	149.1
7-H	7.97–7.90 (m)	7.90–7.86 (m)	7.95–7.91 (m)	7.96–7.90 (m)
7-C	128.0	127.7	127.9	127.8
8-H	7.97–7.90 (m)	7.90–7.86 (m)	7.95–7.91 (m)	7.96–7.90 (m)
8-C	134.5	134.3	134.4	134.5
9-H	7.66–7.61 (m)	7.58 (ddd, *J* = 8.0, 6.1, 2.2)	7.63–7.59 (m)	7.62 (ddd, *J* = 7.7, 5.7, 2.5)
9-C	127.1	126.5	126.9	127.0
10-H	8.30 (dd, *J* = 7.8, 1.0)	8.26 (d, *J* = 7.1)	8.28 (d, *J* = 8.0)	8.30 (d, *J* = 7.7)
10-C	125.9	125.8	125.9	125.9
10a-C	121.0	120.7	121.0	121.0
11-C	159.7	159.7	159.8	159.7
13-H	5.30 (s)	5.20 (s)	5.19 (s)	5.28 (s)
13-C	47.5	47.3	47.3	47.4
13a-C	128.4	131.2 ^b^	126.4	131.1
14-H	8.92 (s)	8.25 (s)	8.41 (s)	8.55 (s)
14-C	126.8	127.3	131.0	131.3
14a-C	118.2	131.1 ^b^	121.6	129.3
NH_2_	6.17 (br s)	6.07 (br s)	6.06 (br s)	6.18 (br s)

^a,b^ assignment ambiguous.

## Supplementary Materials

Spectra (^1^H-NMR, ^13^C-NMR, EI-MS) of all new compounds are available online.
