# Supplementary material for: Position-Selective Synthesis and Biological Evaluation of Four Isomeric A-Ring Amino Derivatives of the Alkaloid Luotonin A
Source: Molecules. 2019 Feb 16;24(4):716. doi: 10.3390/molecules24040716 (PMC6412769; doi:10.3390/molecules24040716)
Supplement: Supplementary file 1 [file molecules-24-00716-s001.pdf]

Position-Selective Synthesis and Biological Evaluation of Four Isomeric A-Ring Amino Derivatives of the Alkaloid Luotonin A

Amra Ibric [1], Stefan Eckerstorfer [1], Martin Eder [1], Ivan Louko [1], Leopold Tunjic [1], Petra Heffeter [2], Hemma Henrike Schueffl [2], Brigitte Marian [2] and Norbert Haider [1*]

[1] Department of Pharmaceutical Chemistry, University of Vienna, Althanstraße 14, A-1090 Vienna, Austria ; amra.ibric@univie.ac.at (A.I.) ; stefan.eckerstorfer@me.com (S.E.) ; semrad@gmx.at (M.E.) ; ivan_louko@yahoo.com (I.L.) ; leopold.tunjic@gmail.com (L.T.) ; norbert.haider@univie.ac.at (N.H.)

[2] Institute of Cancer Research and Comprehensive Cancer Center, Medical University of Vienna, Borschkegasse 8a, A-1090 Vienna, Austria ; petra.heffeter@meduniwien.ac.at (P.H.) ; hemma.schueffl@meduniwien.ac.at (H.H.S) ; brigitte.marian@meduniwien.ac.at (B.M.) ;

* Correspondence: norbert.haider@univie.ac.at; Tel.: +43-1-4277-55624

Supplementary Material

IL1; 2-Nitrophenyl-Amid / DMSO 1H

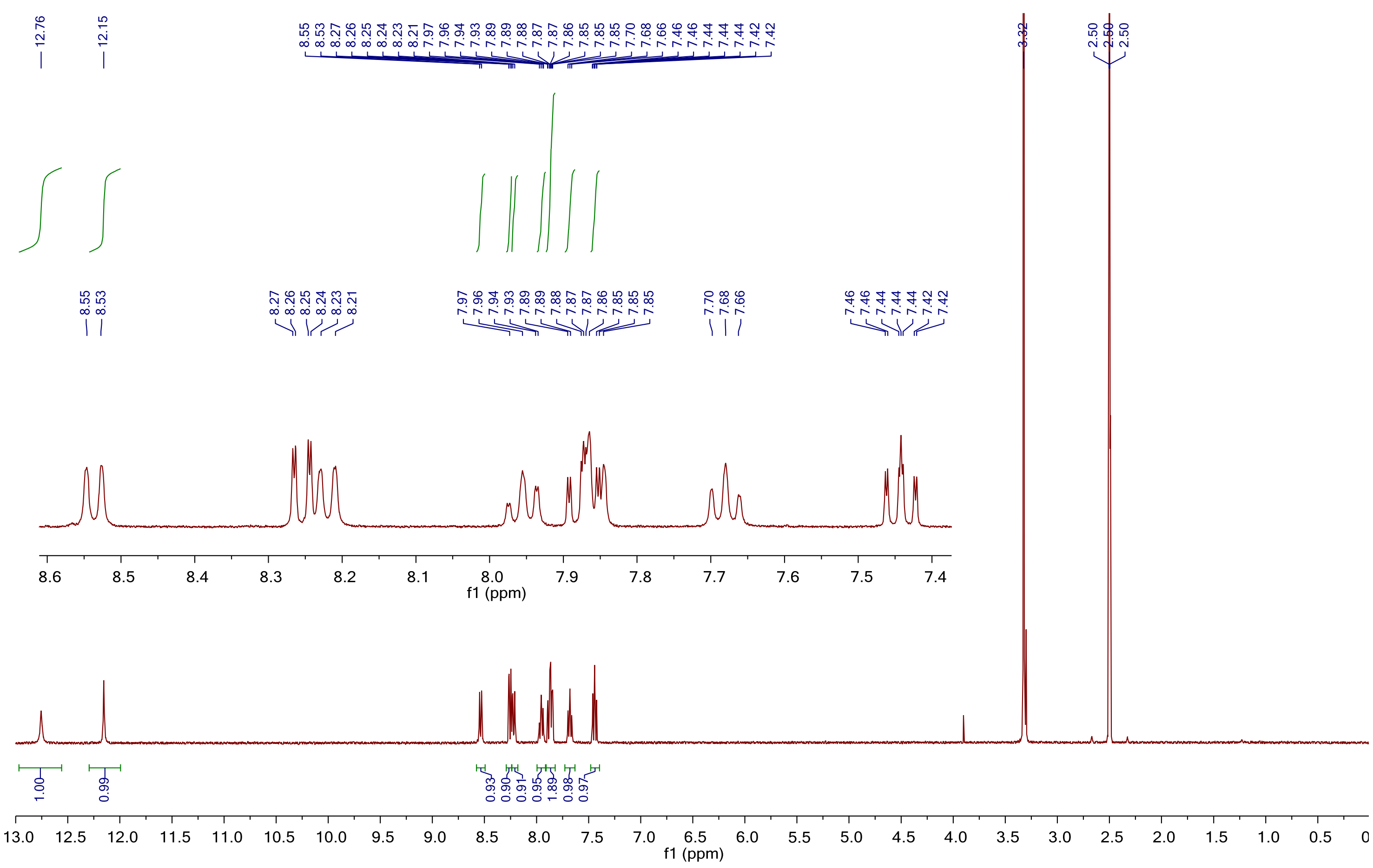

Figure S1. ^{1}H NMR spoectrum of *N*-(2-nitrophenyl)-4-oxo-3,4-dihydroquinazoline-2-carboxamide (**2b**).

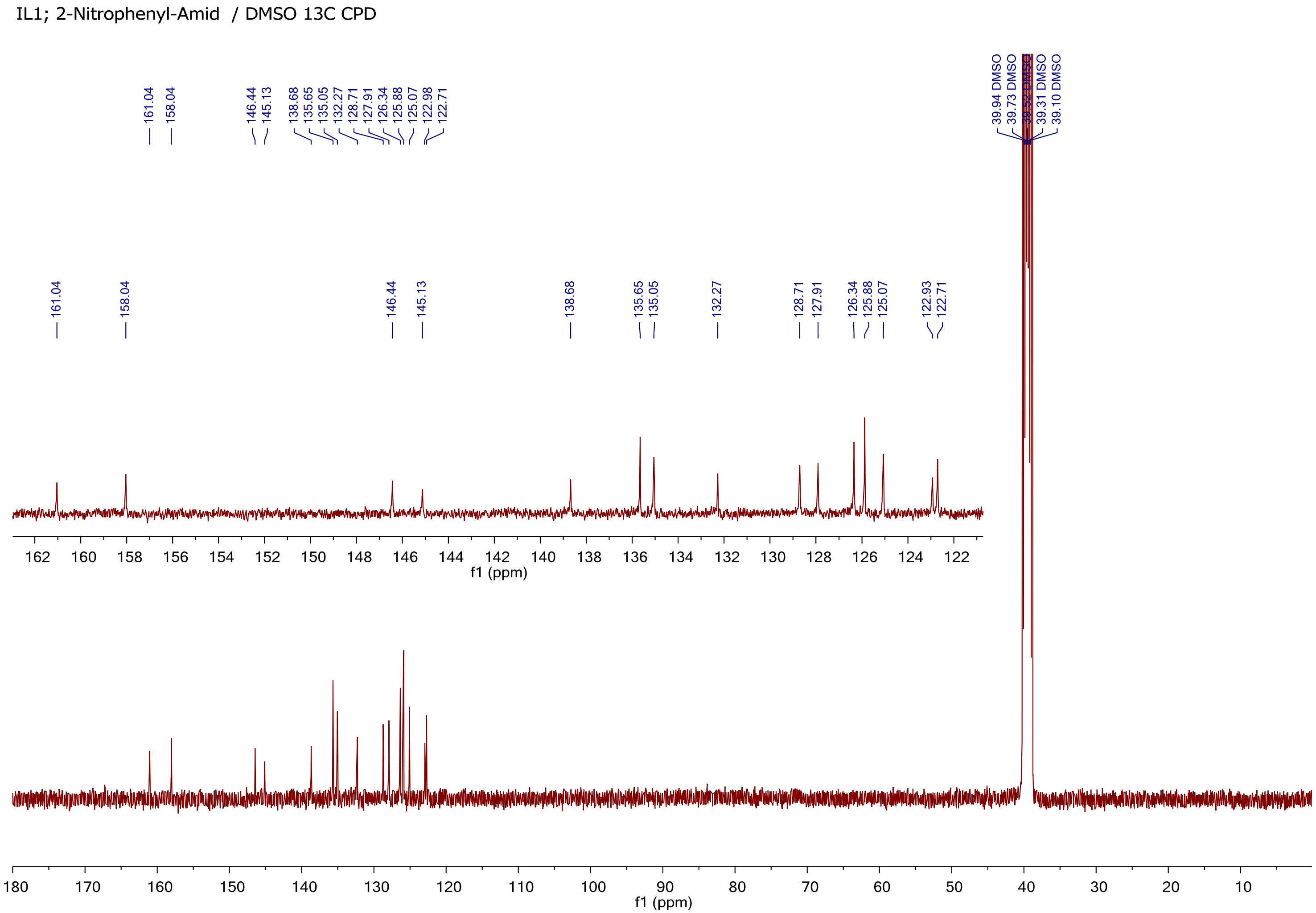

Figure S2. ^{13}C NMR spoectrum of *N*-(2-nitrophenyl)-4-oxo-3,4-dihydroquinazoline-2-carboxamide (**2b**).

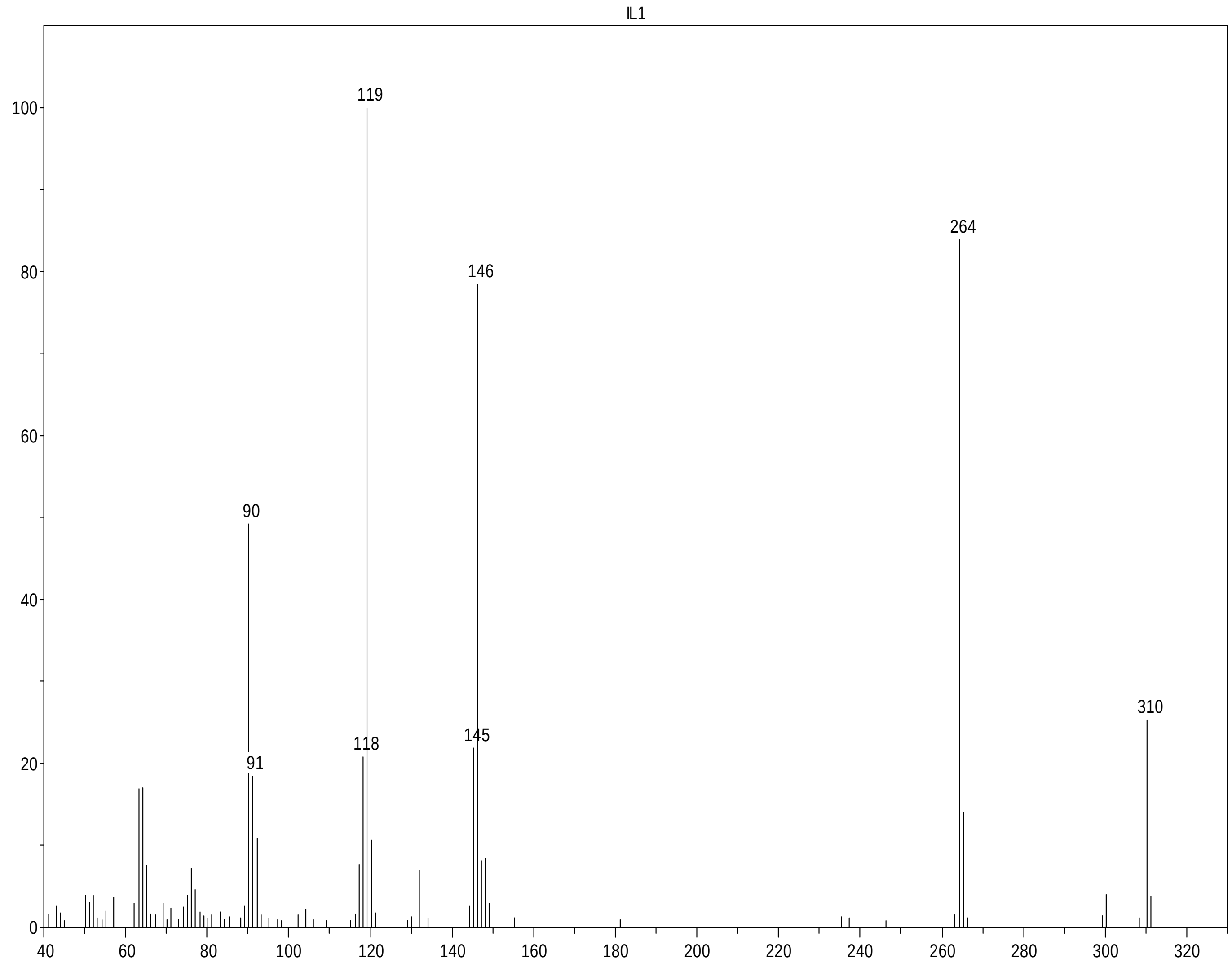

Figure S3. EI-MS of *N*-(2-nitrophenyl)-4-oxo-3,4-dihydroquinazoline-2-carboxamide (**2b**).

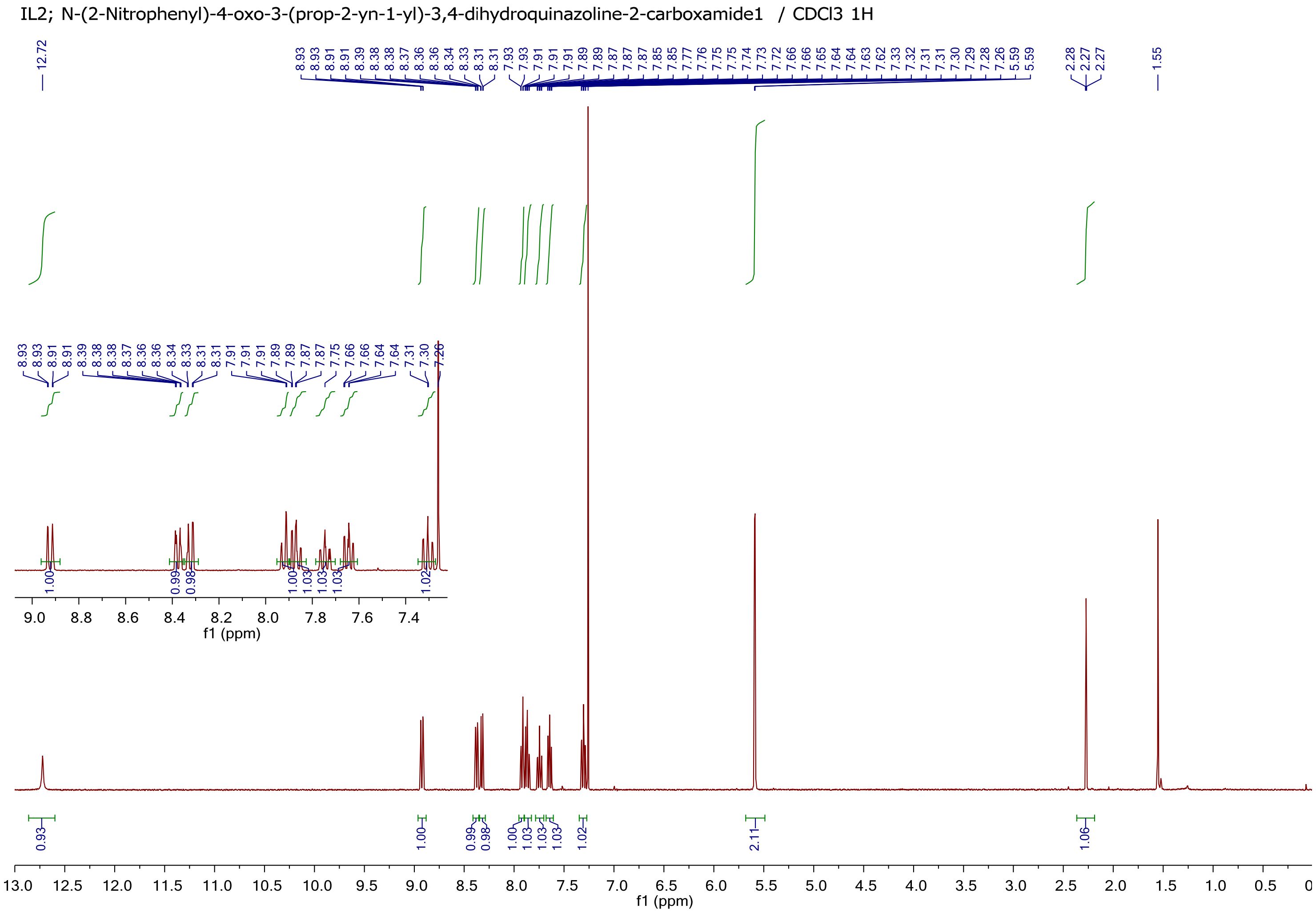

Figure S4. ^{1}H NMR spoectrum of *N*-(2-nitrophenyl)-4-oxo-3-(prop-2-yn-1-yl)-3,4-dihydroquinazoline-2-carboxamide (**3b**).

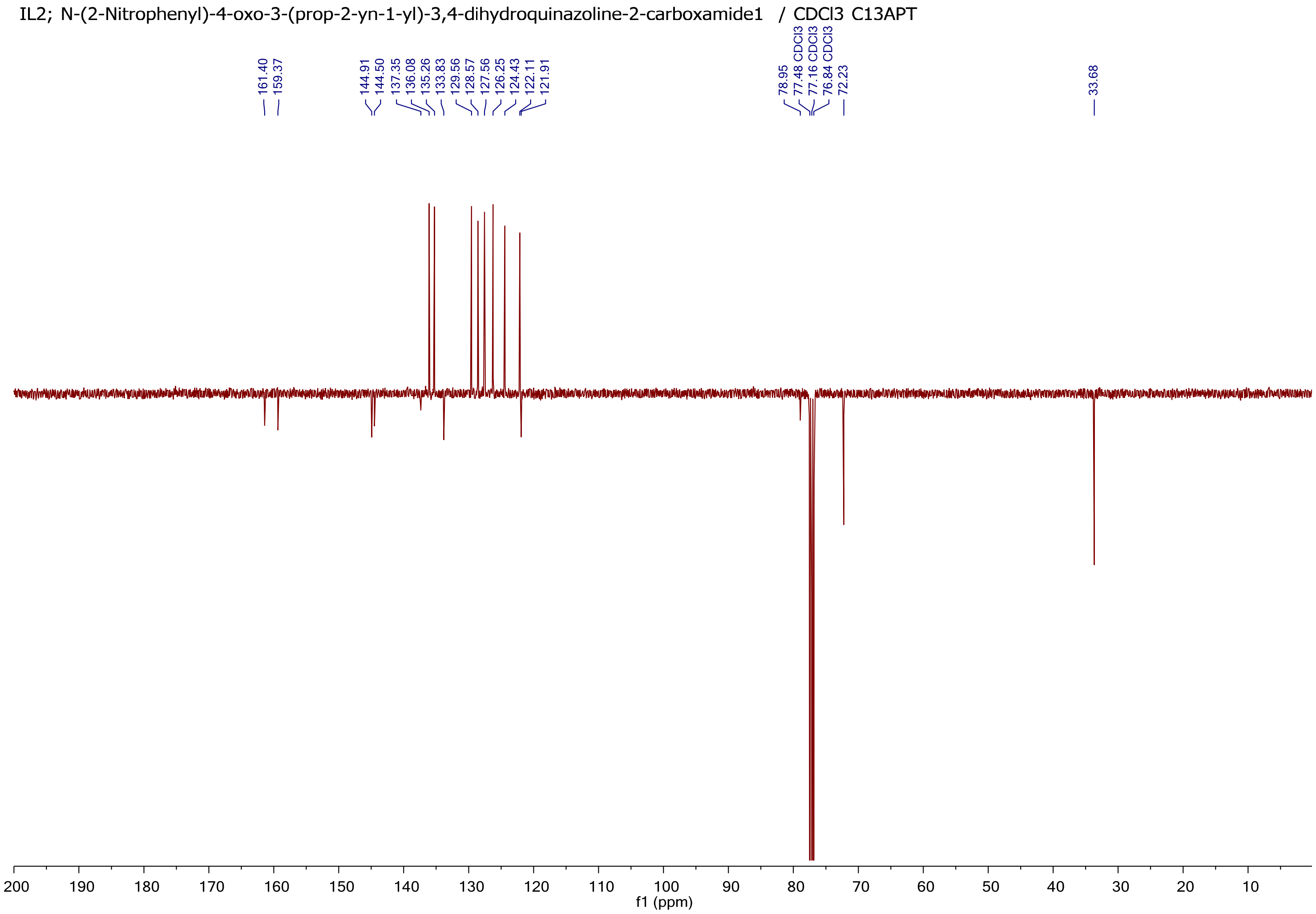

Figure S5. ^{13}C NMR spoectrum of *N*-(2-nitrophenyl)-4-oxo-3-(prop-2-yn-1-yl)-3,4-dihydroquinazoline-2-carboxamide (**3b**).

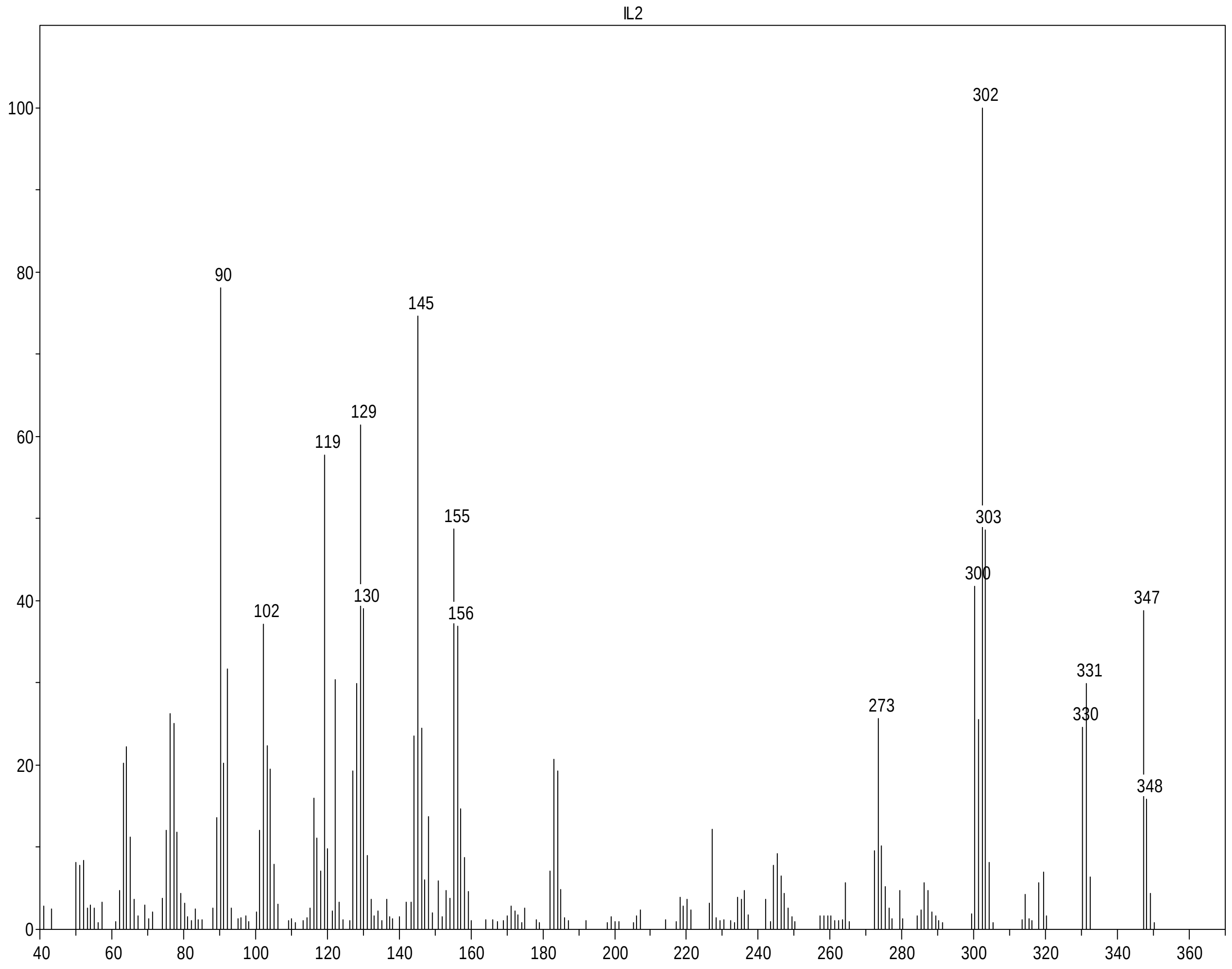

Figure S6. EI-MS of *N*-(2-nitrophenyl)-4-oxo-3-(prop-2-yn-1-yl)-3,4-dihydroquinazoline-2-carboxamide (**3b**).

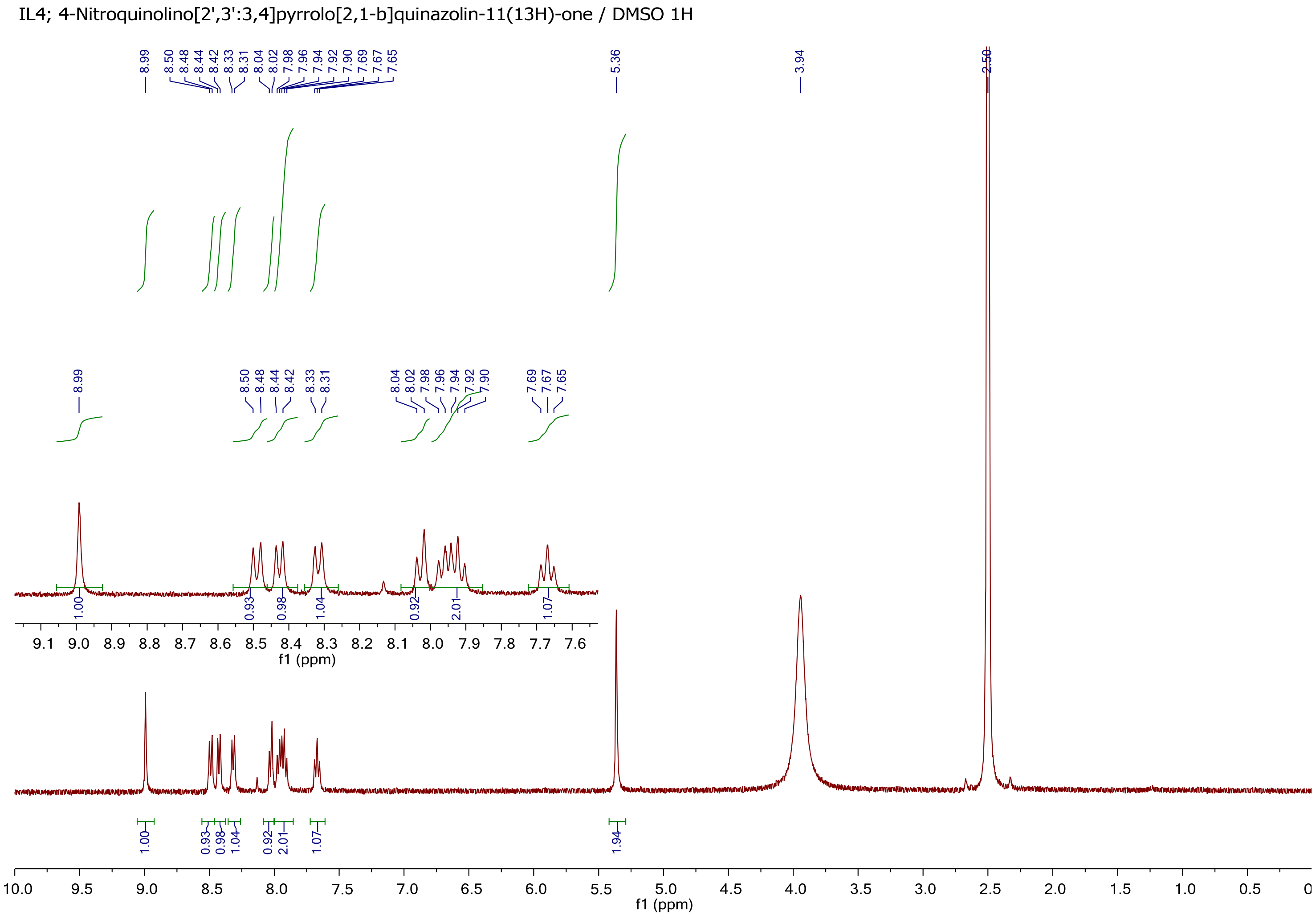

Figure S7. ^{1}H NMR spoectrum of 4-Nitroquinolino[2',3':3,4]pyrrolo[2,1-*b*]quinazolin-11(13*H*)-one (**4b**).

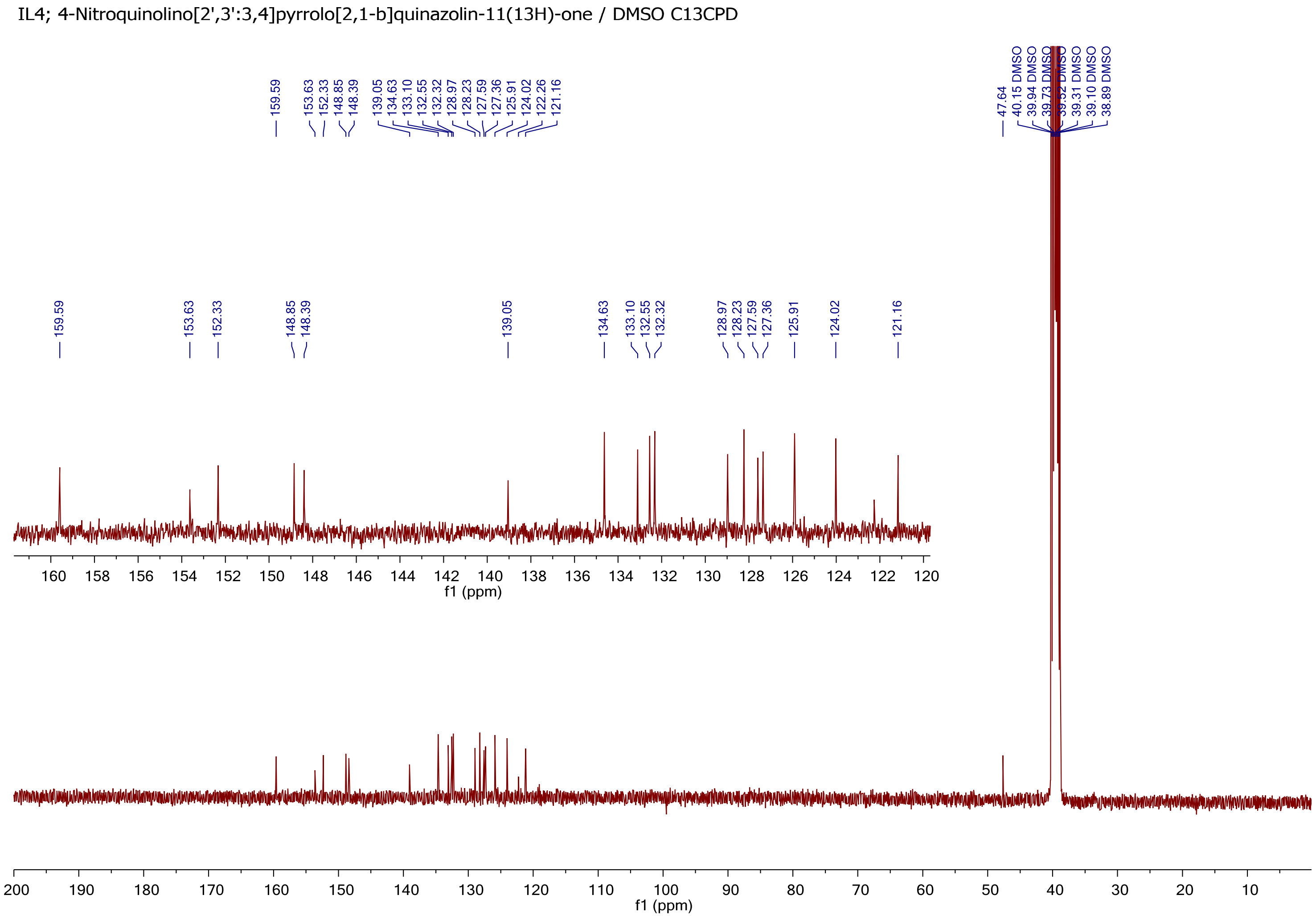

Figure S8. ^{13}C NMR spoectrum of 4-Nitroquinolino[2',3':3,4]pyrrolo[2,1-*b*]quinazolin-11(13*H*)-one (**4b**).

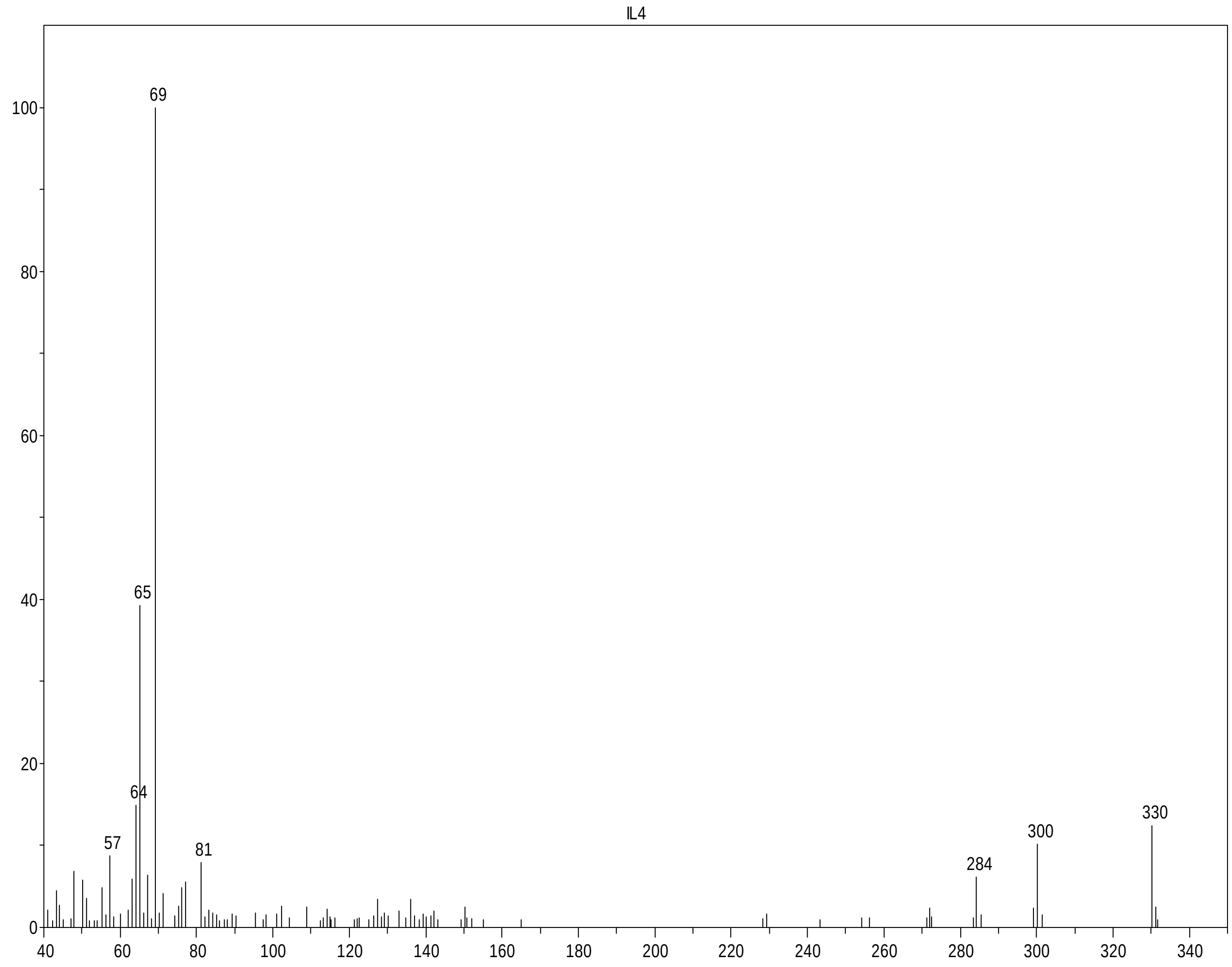

Figure S9. EI-MS of 4-Nitroquinolino[2',3':3,4]pyrrolo[2,1-*b*]quinazolin-11(13*H*)-one (**4b**).

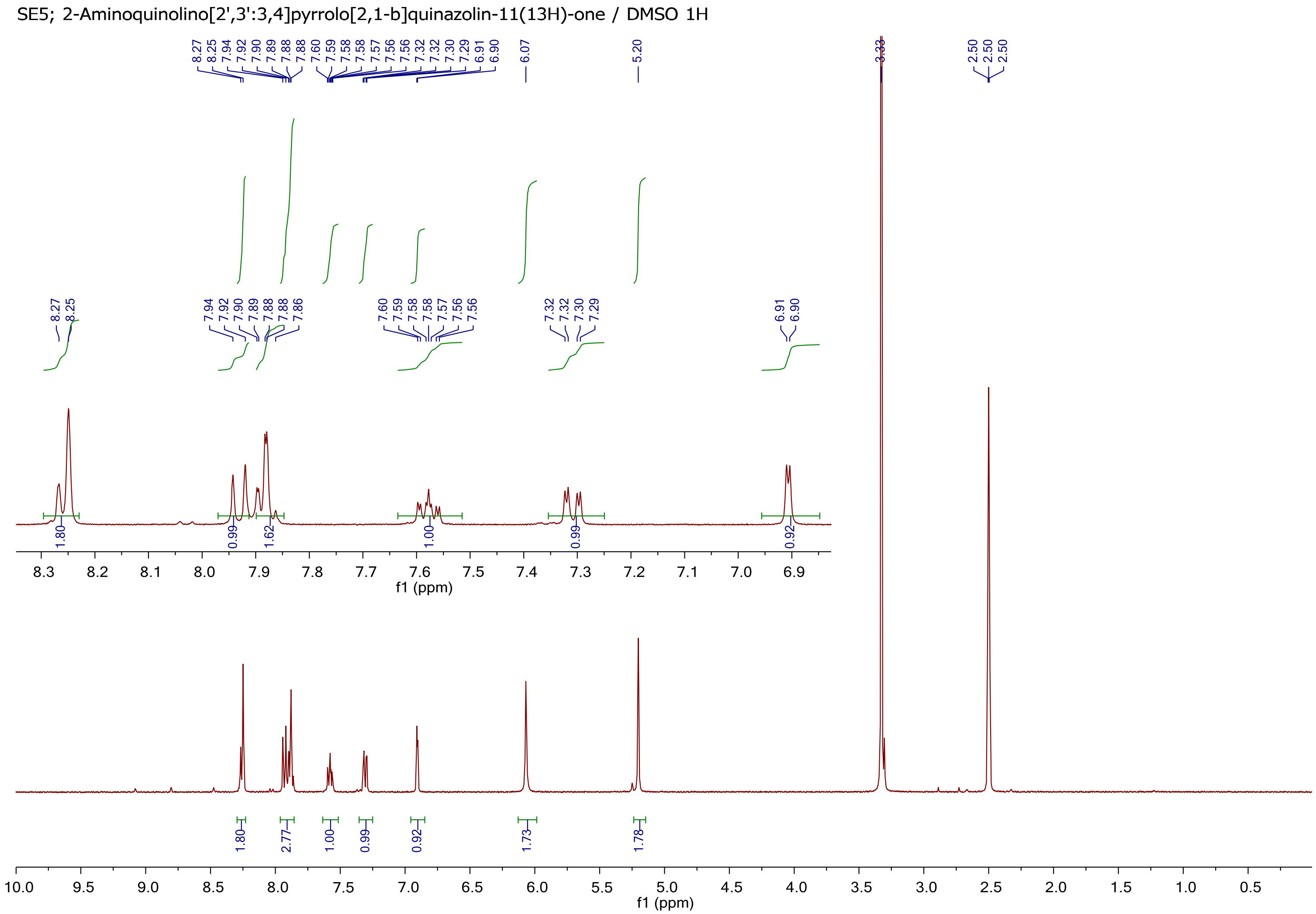

Figure S10. ^{1}H NMR spoectrum of 2-aminoquinolino[2',3':3,4]pyrrolo[2,1-*b*]quinazolin-11(13*H*)-one (**5a**).

SE5; 2-Aminoquinolino[2',3':3,4]pyrrolo[2,1-b]quinazolin-11(13H)-one / DMSO COSY

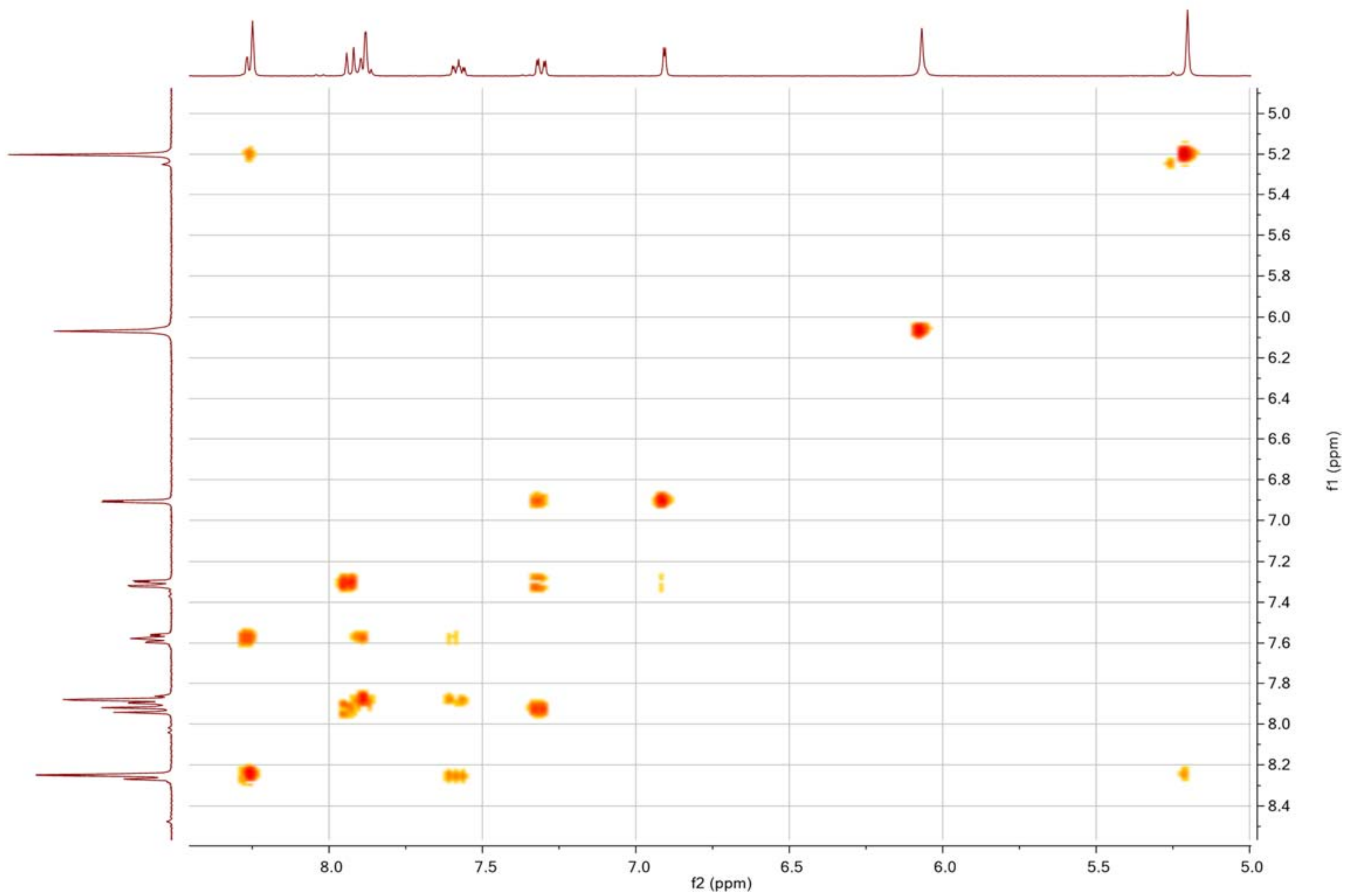

Figure S11. COSY spoectrum of 2-aminoquinolino[2',3':3,4]pyrrolo[2,1-*b*]quinazolin-11(13*H*)-one (**5a**).

SE5; 2-Aminoquinolino[2',3':3,4]pyrrolo[2,1-b]quinazolin-11(13H)-one / DMSO NOESY

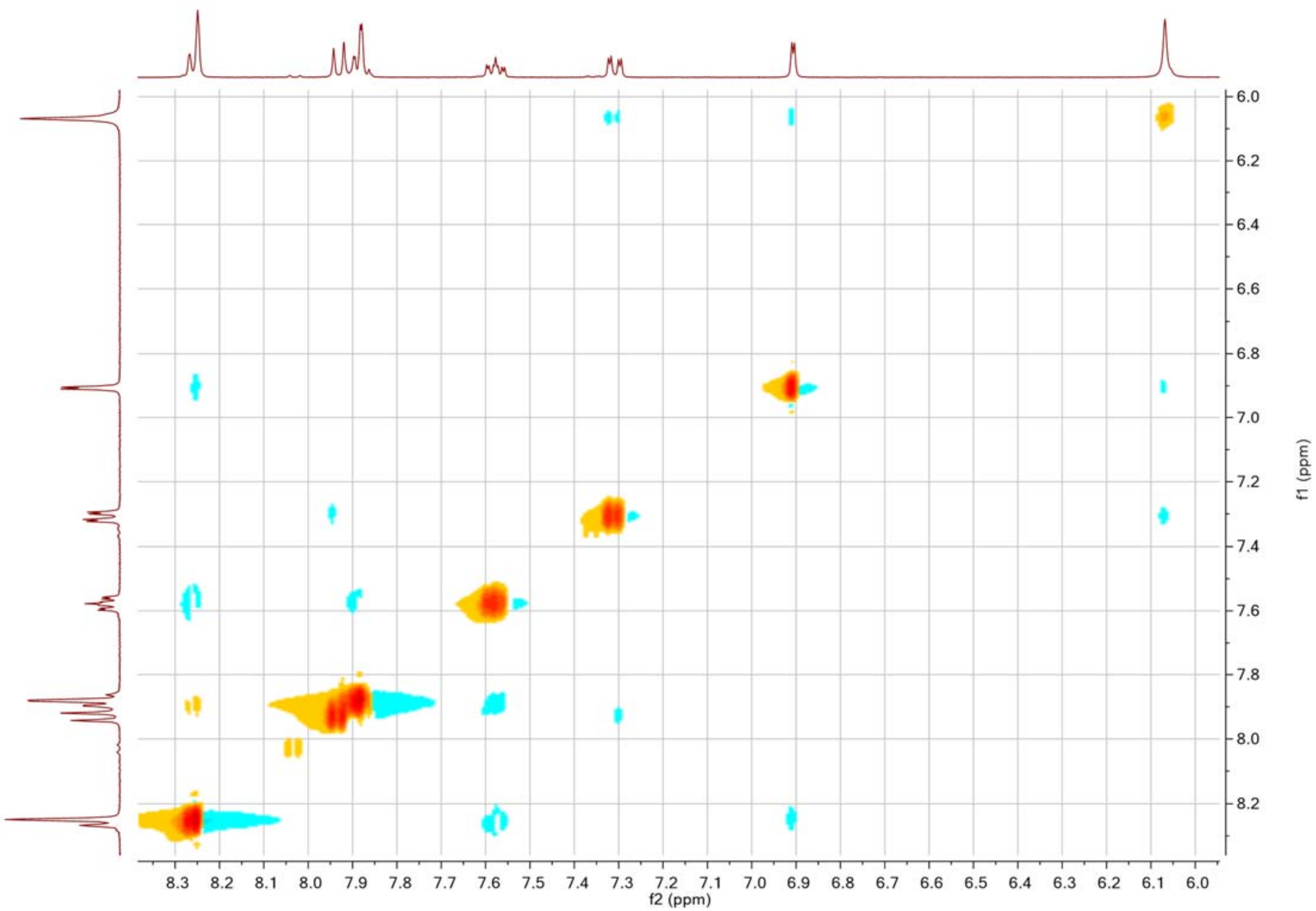

Figure S12. NOESY spoectrum of 2-aminoquinolino[2',3':3,4]pyrrolo[2,1-*b*]quinazolin-11(13*H*)-one (**5a**).

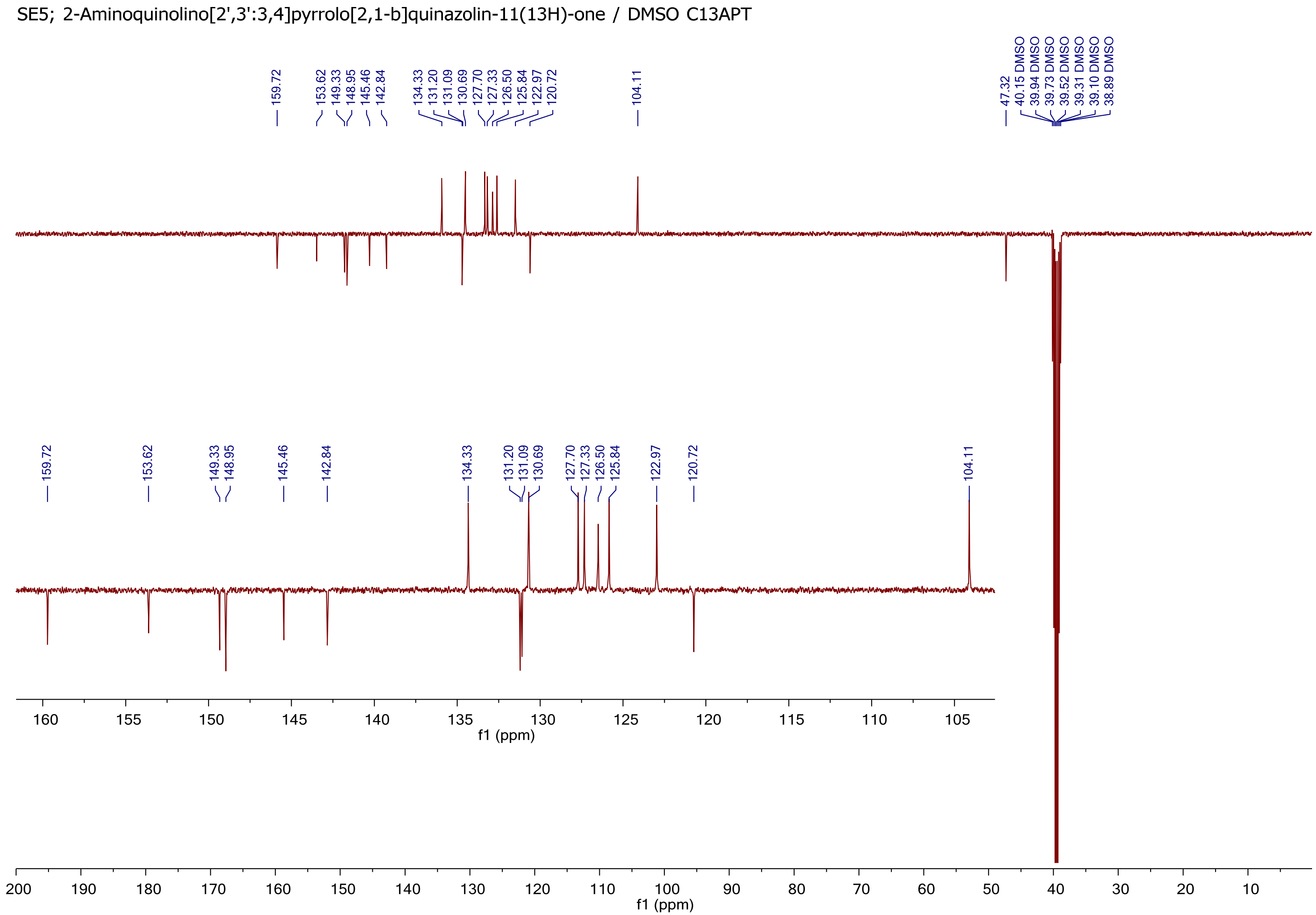

Figure S13. ^{13}C NMR spoectrum of 2-aminoquinolino[2',3':3,4]pyrrolo[2,1-*b*]quinazolin-11(13*H*)-one (**5a**).

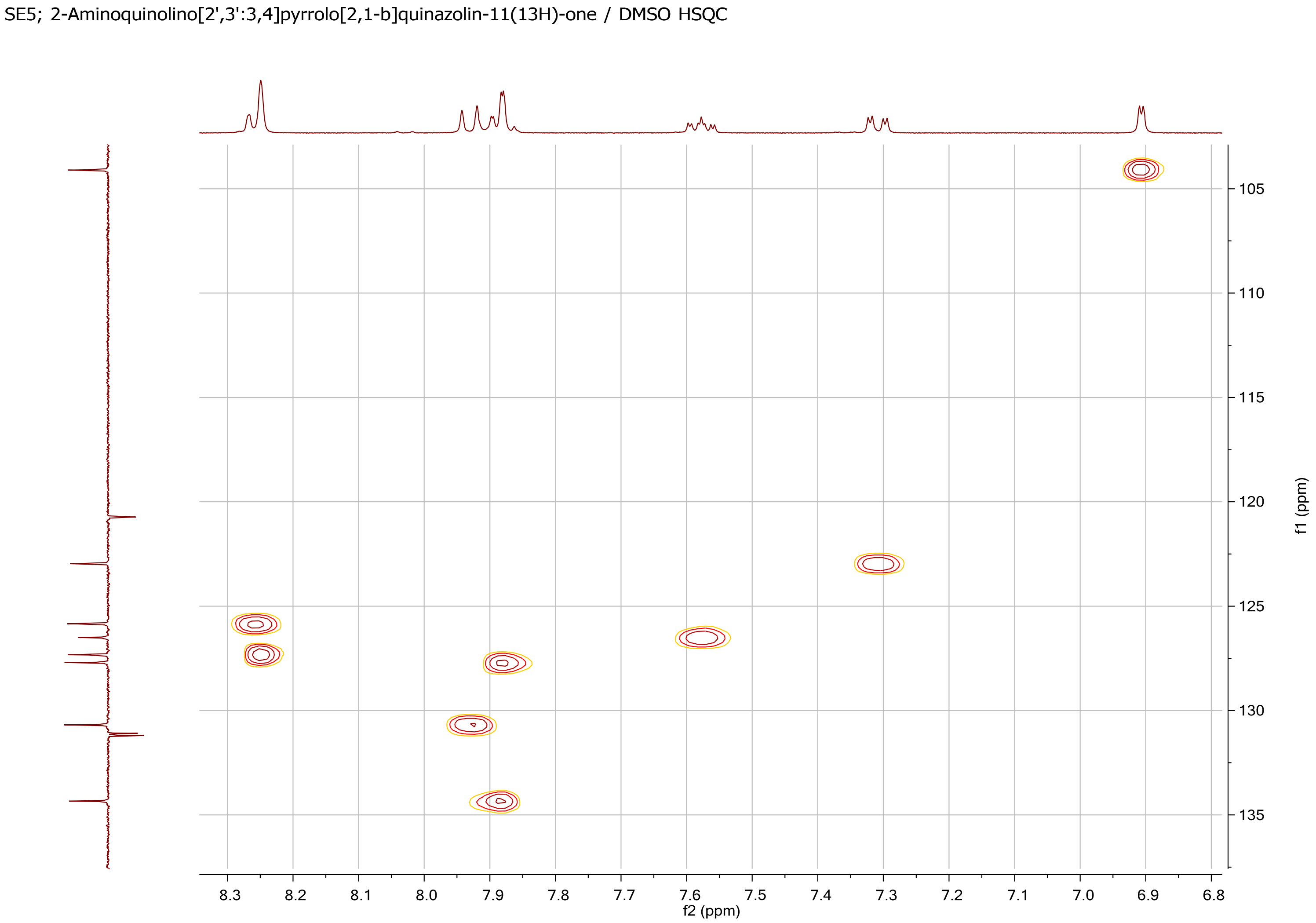

Figure S14. HSQC spoectrum of 2-aminoquinolino[2',3':3,4]pyrrolo[2,1-*b*]quinazolin-11(13*H*)-one (**5a**).

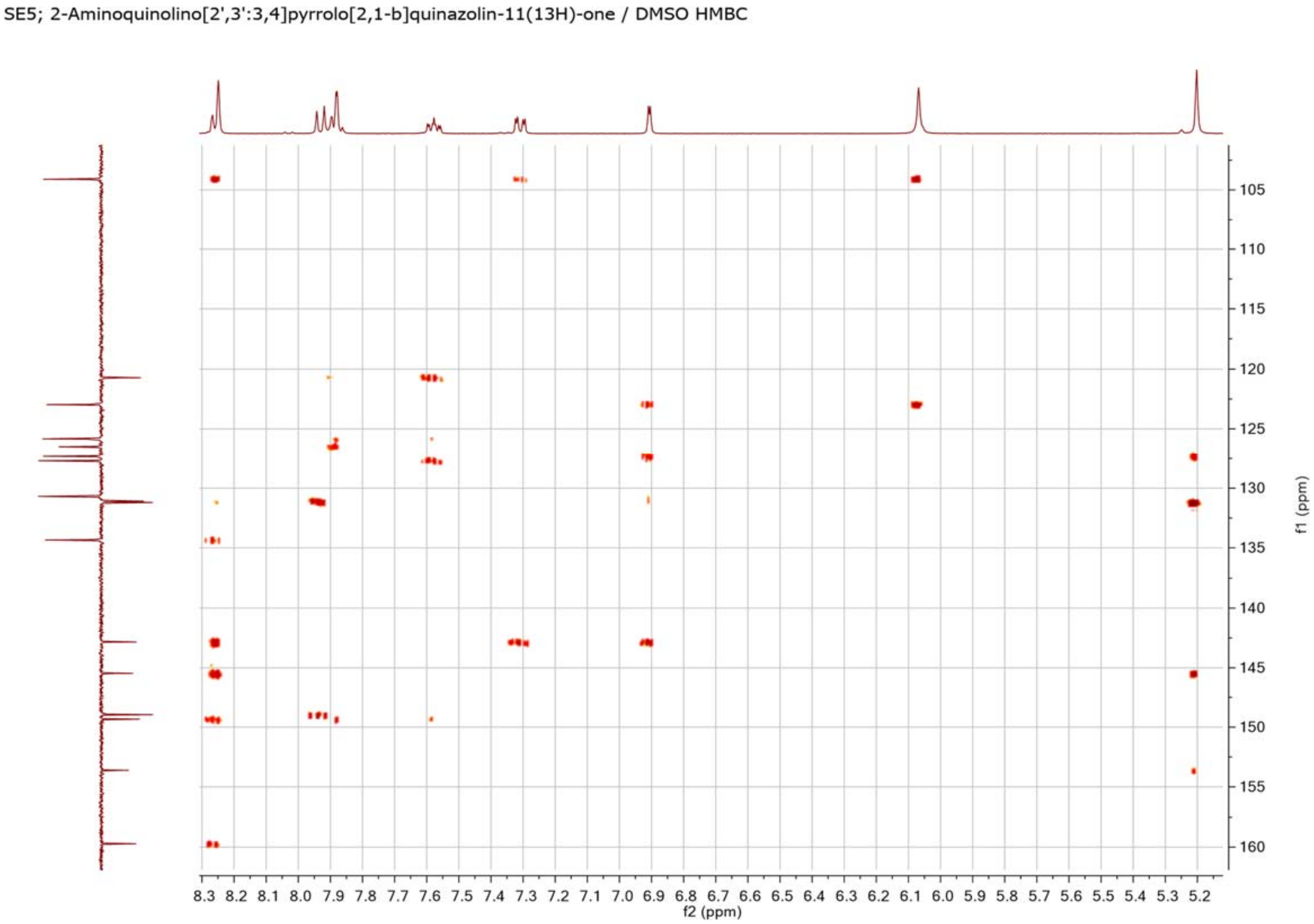

Figure S15. HMBC spoectrum of 2-aminoquinolino[2',3':3,4]pyrrolo[2,1-*b*]quinazolin-11(13*H*)-one (**5a**).

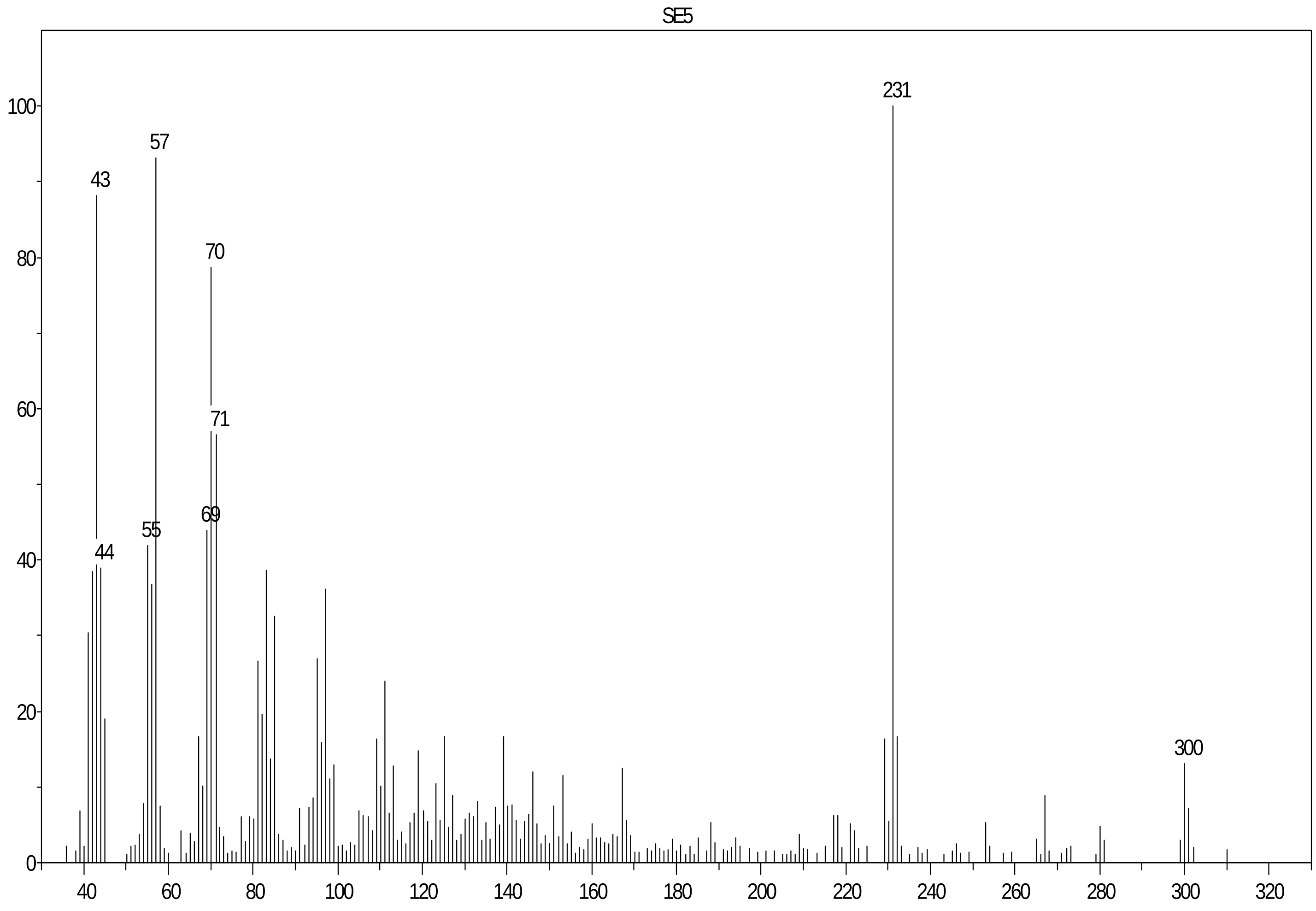

Figure S16. EI-MS of 2-aminoquinolino[2',3':3,4]pyrrolo[2,1-*b*]quinazolin-11(13*H*)-one (**5a**).

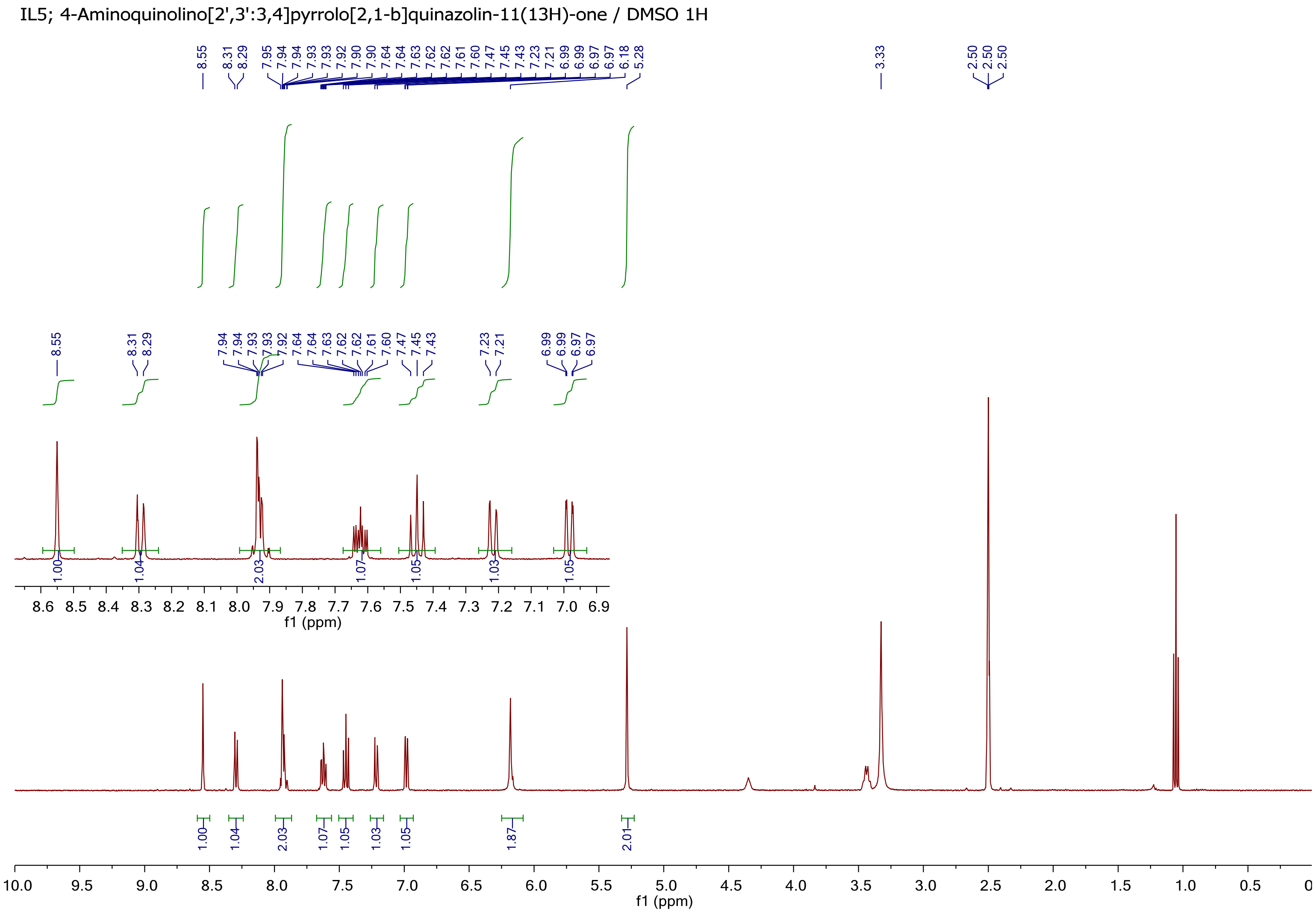

Figure S17. ^{1}H NMR spoectrum of 4-aminoquinolino[2',3':3,4]pyrrolo[2,1-*b*]quinazolin-11(13*H*)-one (**5b**).

IL5; 4-Aminoquinolino[2',3':3,4]pyrrolo[2,1-b]quinazolin-11(13H)-one / DMSO COSY

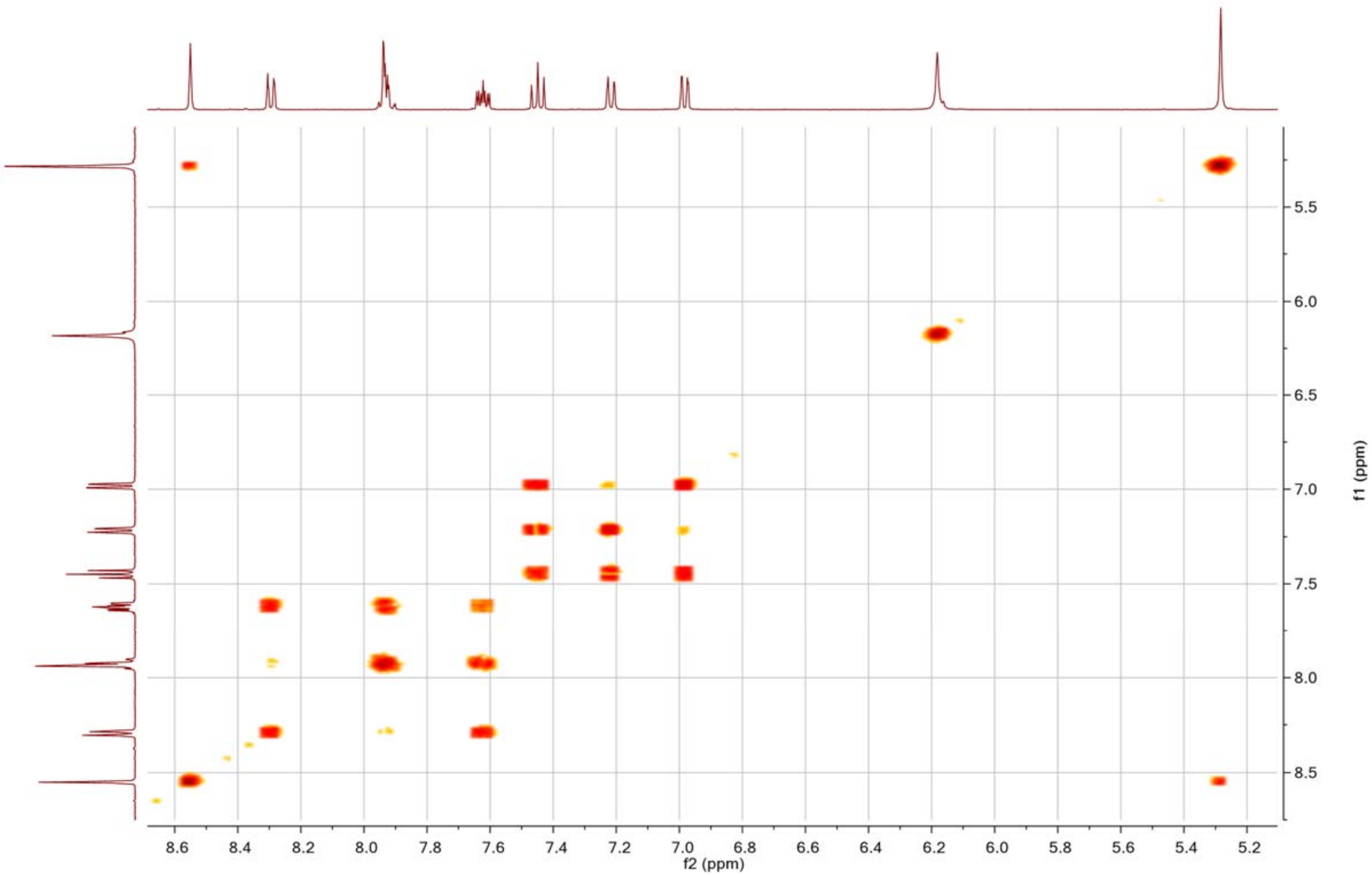

Figure S18. COSY spoectrum of 4-aminoquinolino[2',3':3,4]pyrrolo[2,1-*b*]quinazolin-11(13*H*)-one (**5b**).

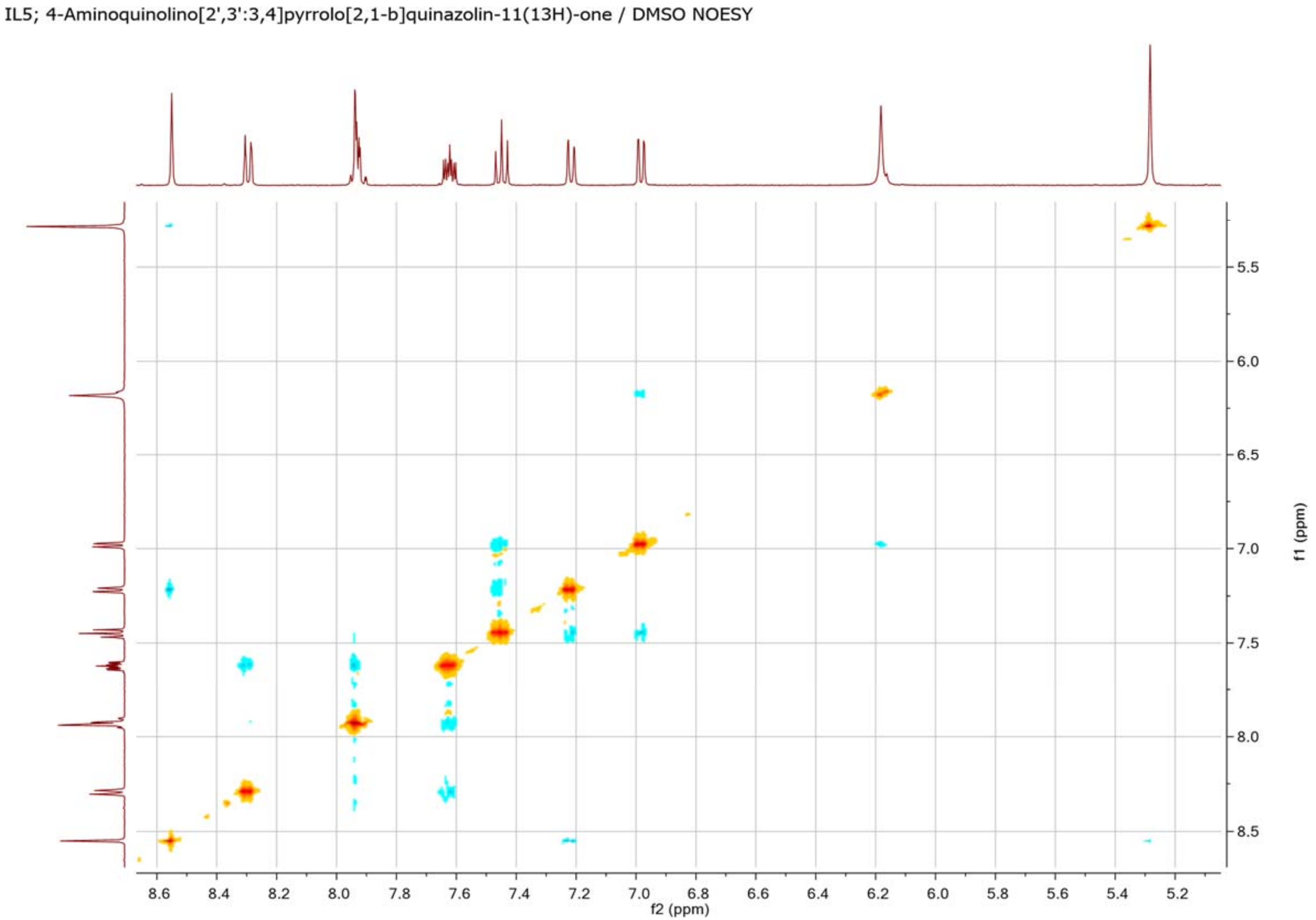

Figure S19. NOESY spoectrum of 4-aminoquinolino[2',3':3,4]pyrrolo[2,1-*b*]quinazolin-11(13*H*)-one (**5b**).

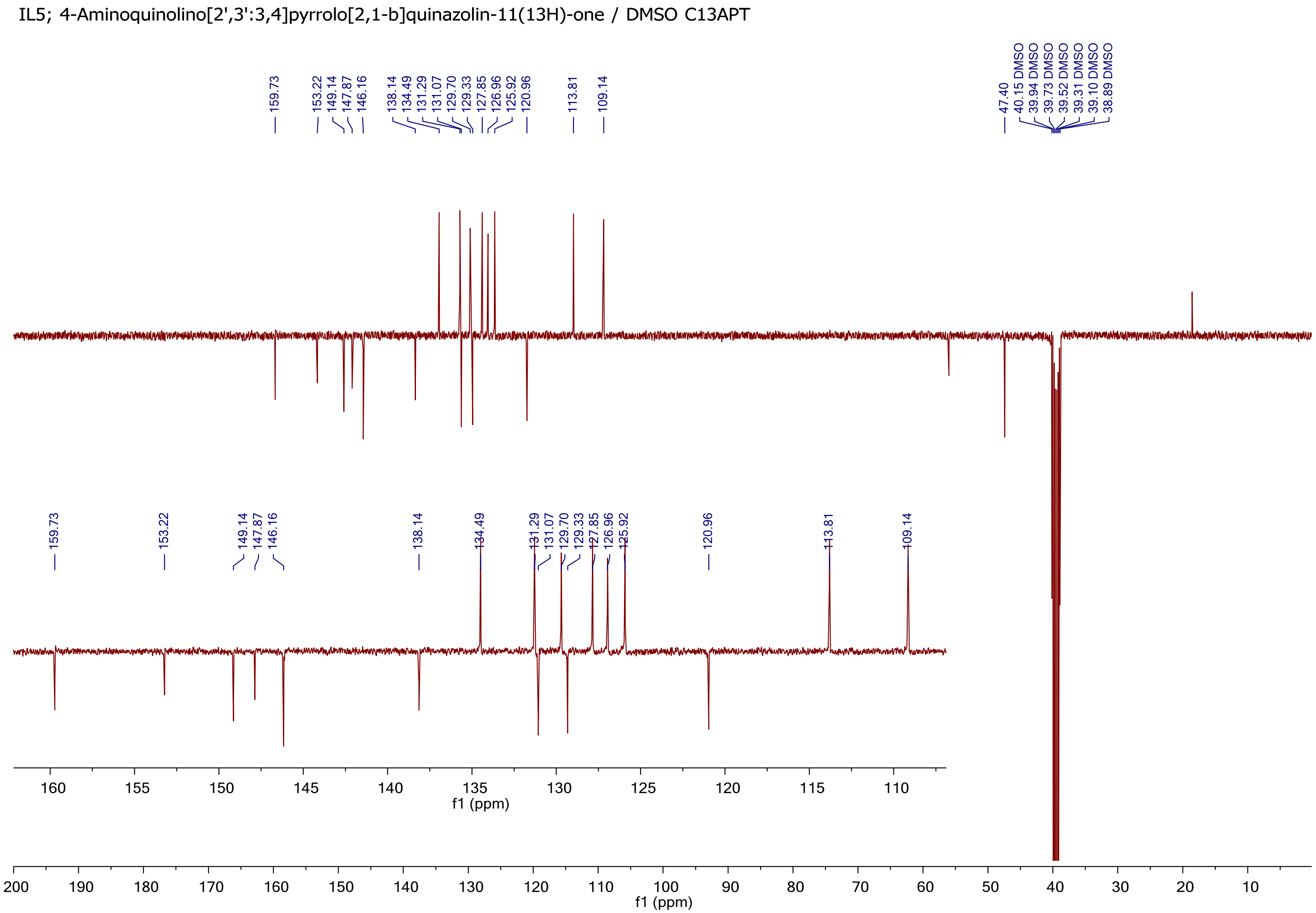

Figure S20. ^{13}C NMR spoectrum of 4-aminoquinolino[2',3':3,4]pyrrolo[2,1-*b*]quinazolin-11(13*H*)-one (**5b**).

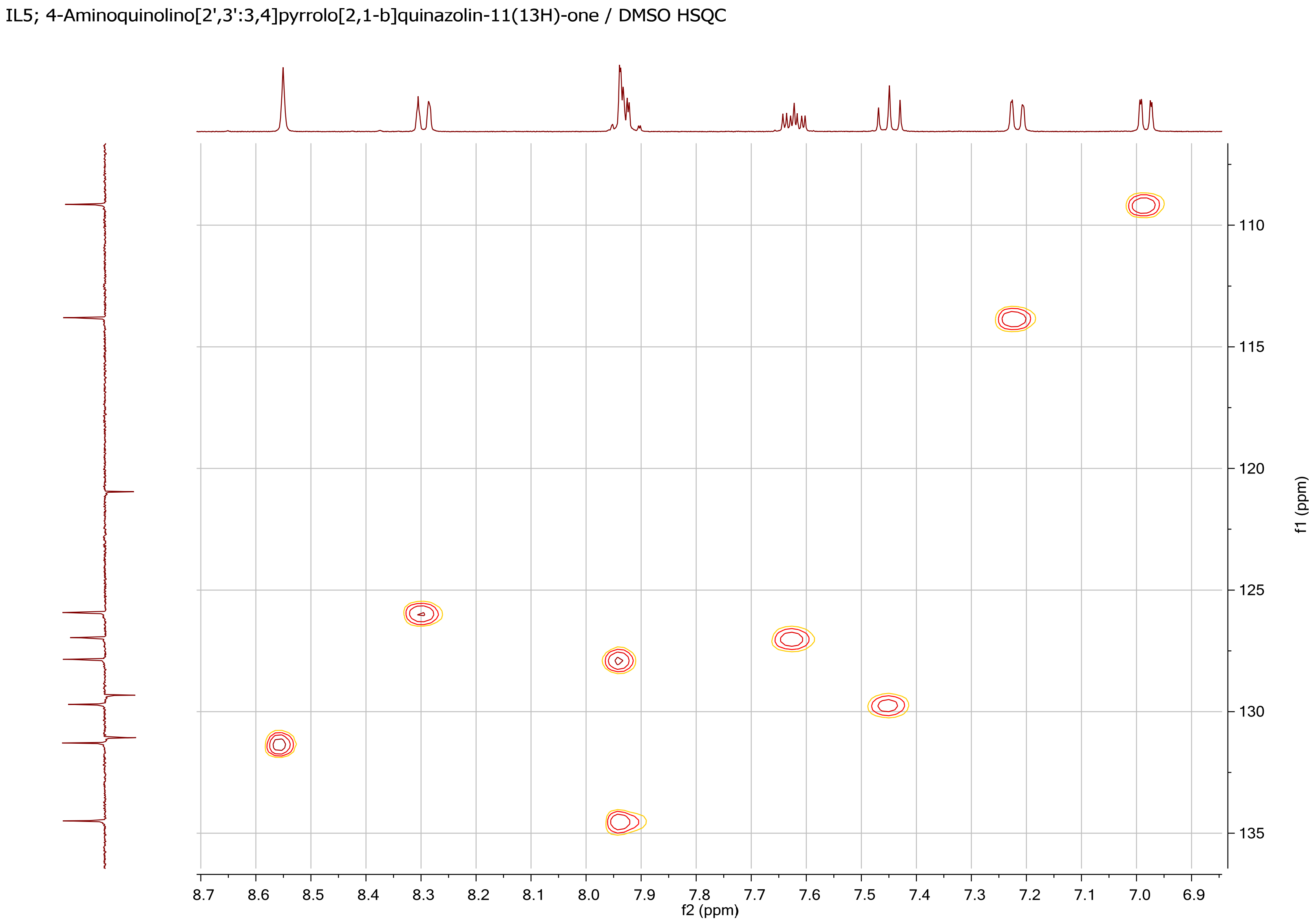

Figure S21. HSQC spoectrum of 4-aminoquinolino[2',3':3,4]pyrrolo[2,1-*b*]quinazolin-11(13*H*)-one (**5b**).

IL5; 4-Aminoquinolino[2',3':3,4]pyrrolo[2,1-b]quinazolin-11(13H)-one / DMSO HMBC

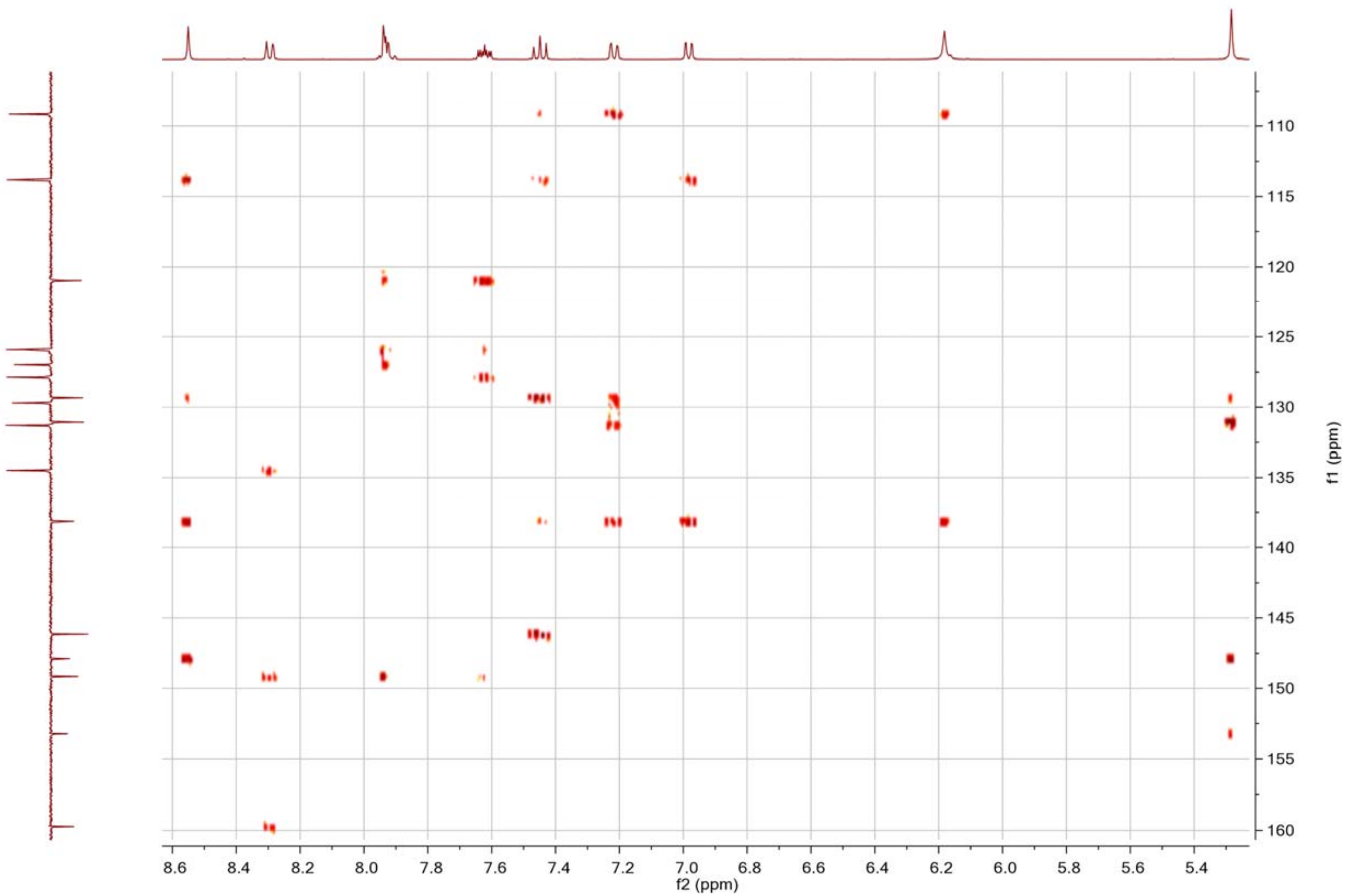

Figure S22. HMBC spoectrum of 4-aminoquinolino[2',3':3,4]pyrrolo[2,1-*b*]quinazolin-11(13*H*)-one (**5b**).

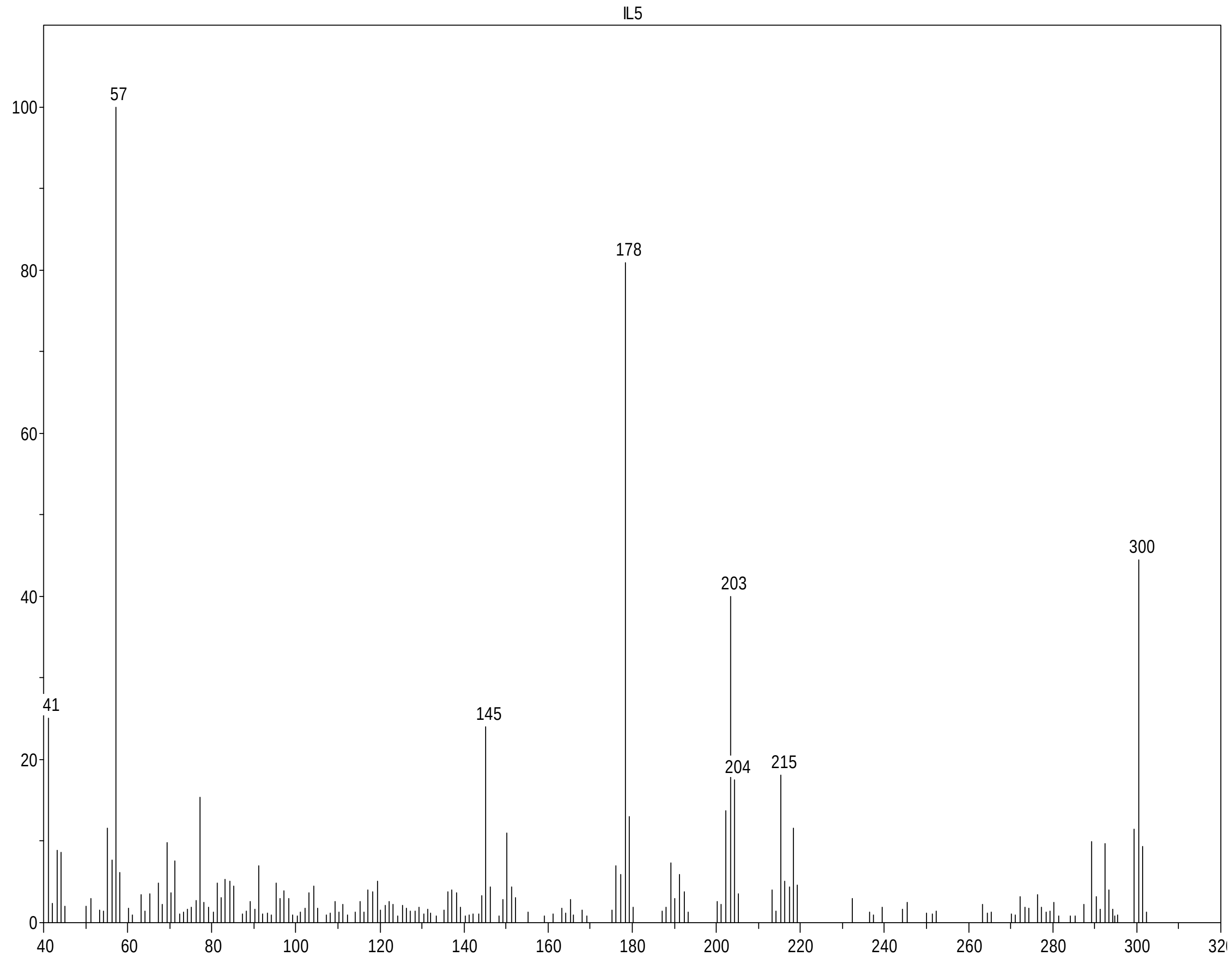

Figure S23. EI-MS of 4-aminoquinolino[2',3':3,4]pyrrolo[2,1-*b*]quinazolin-11(13*H*)-one (**5b**).

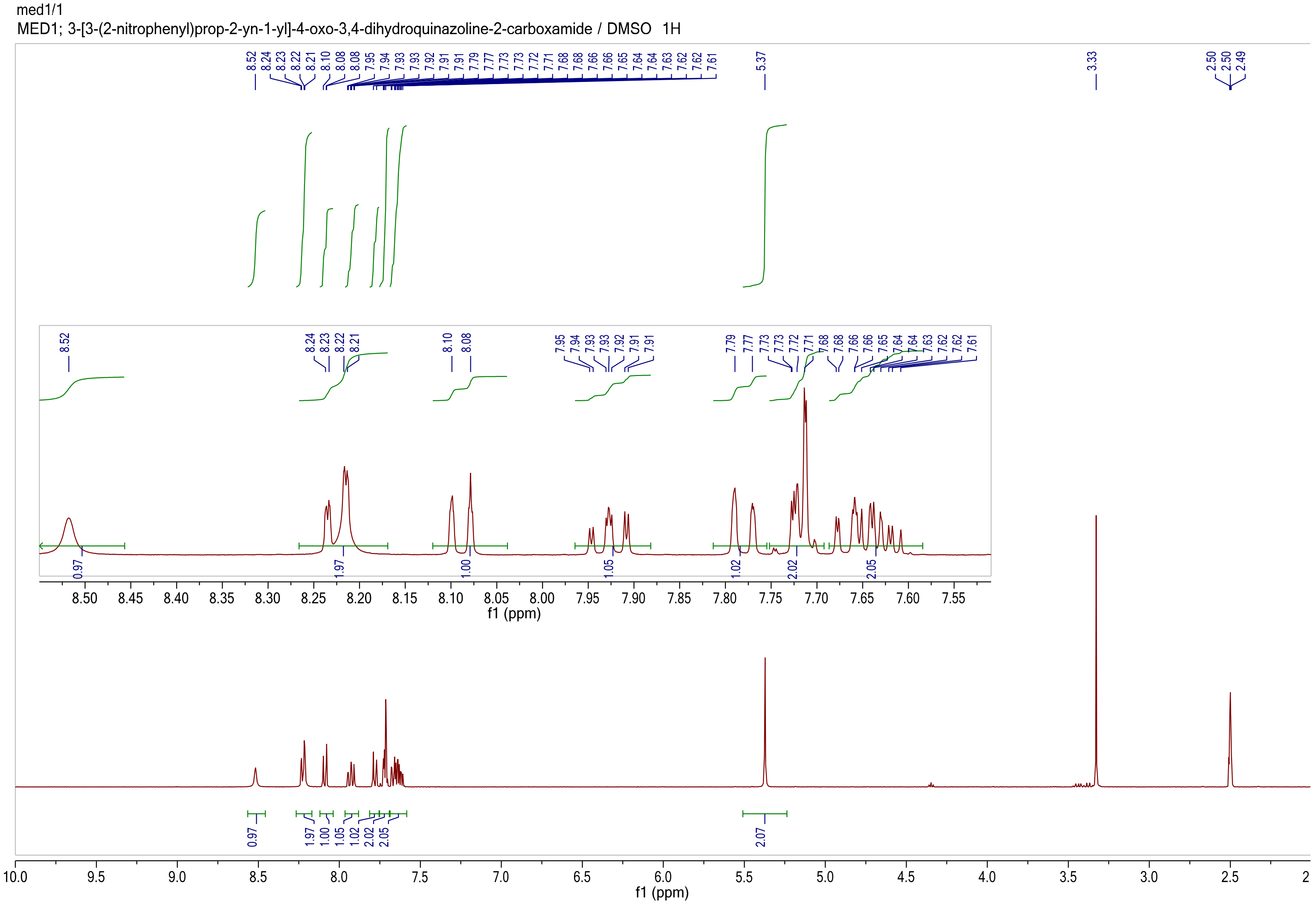

Figure S24. ^{1}H NMR spoectrum of 3-[3-(2-nitrophenyl)prop-2-yn-1-yl]-4-oxo-3,4-dihydroquinazoline-2-carboxamide (**7a**).

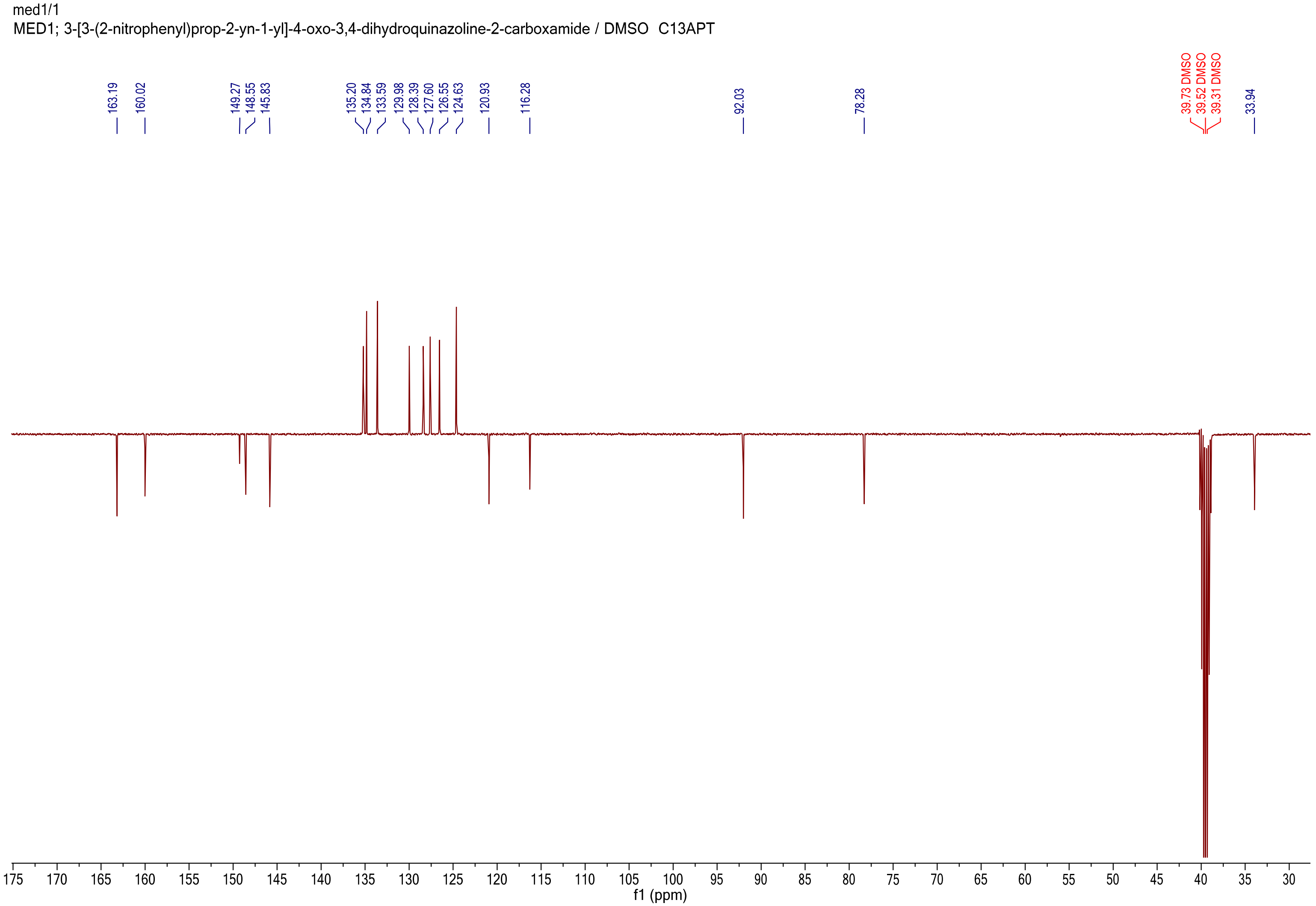

Figure S25. ^{13}C NMR spoectrum of 3-[3-(2-nitrophenyl)prop-2-yn-1-yl]-4-oxo-3,4-dihydroquinazoline-2-carboxamide (**7a**).

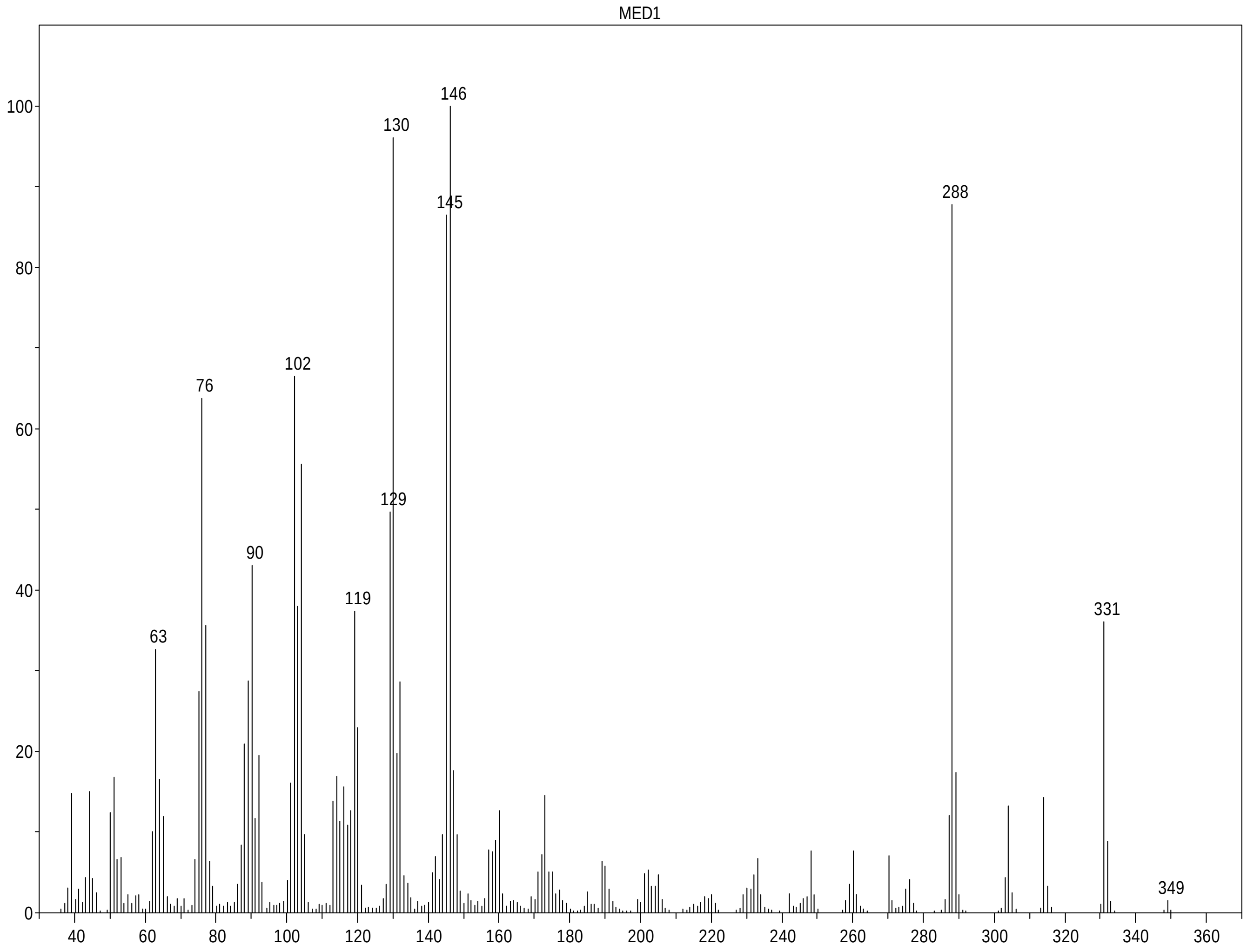

Figure S26. EI-MS of 3-[3-(2-nitrophenyl)prop-2-yn-1-yl]-4-oxo-3,4-dihydroquinazoline-2-carboxamide (**7a**).

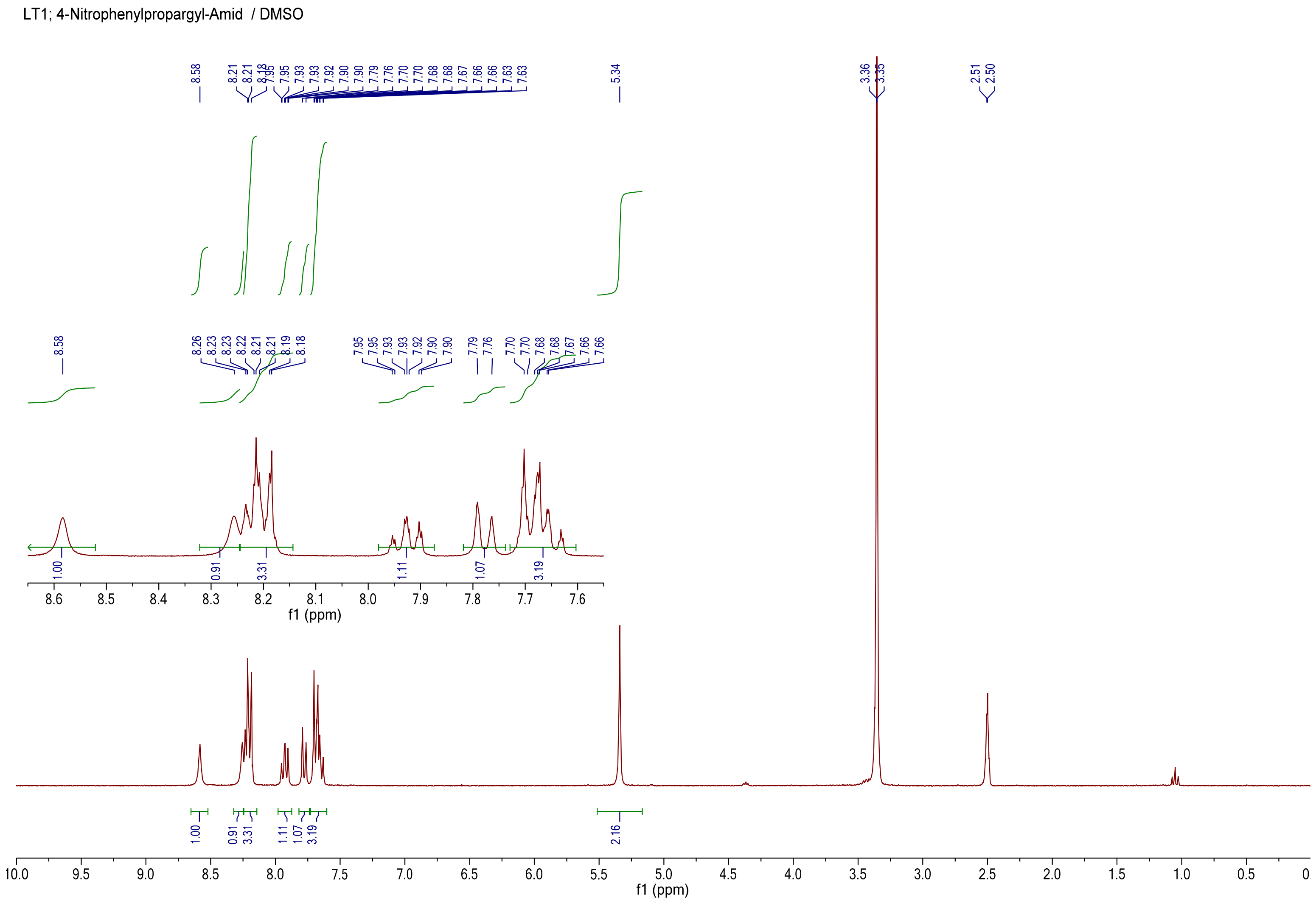

Figure S27. ^{1}H NMR spoectrum of 3-[3-(4-nitrophenyl)prop-2-yn-1-yl]-4-oxo-3,4-dihydroquinazoline-2-carboxamide (**7b**).

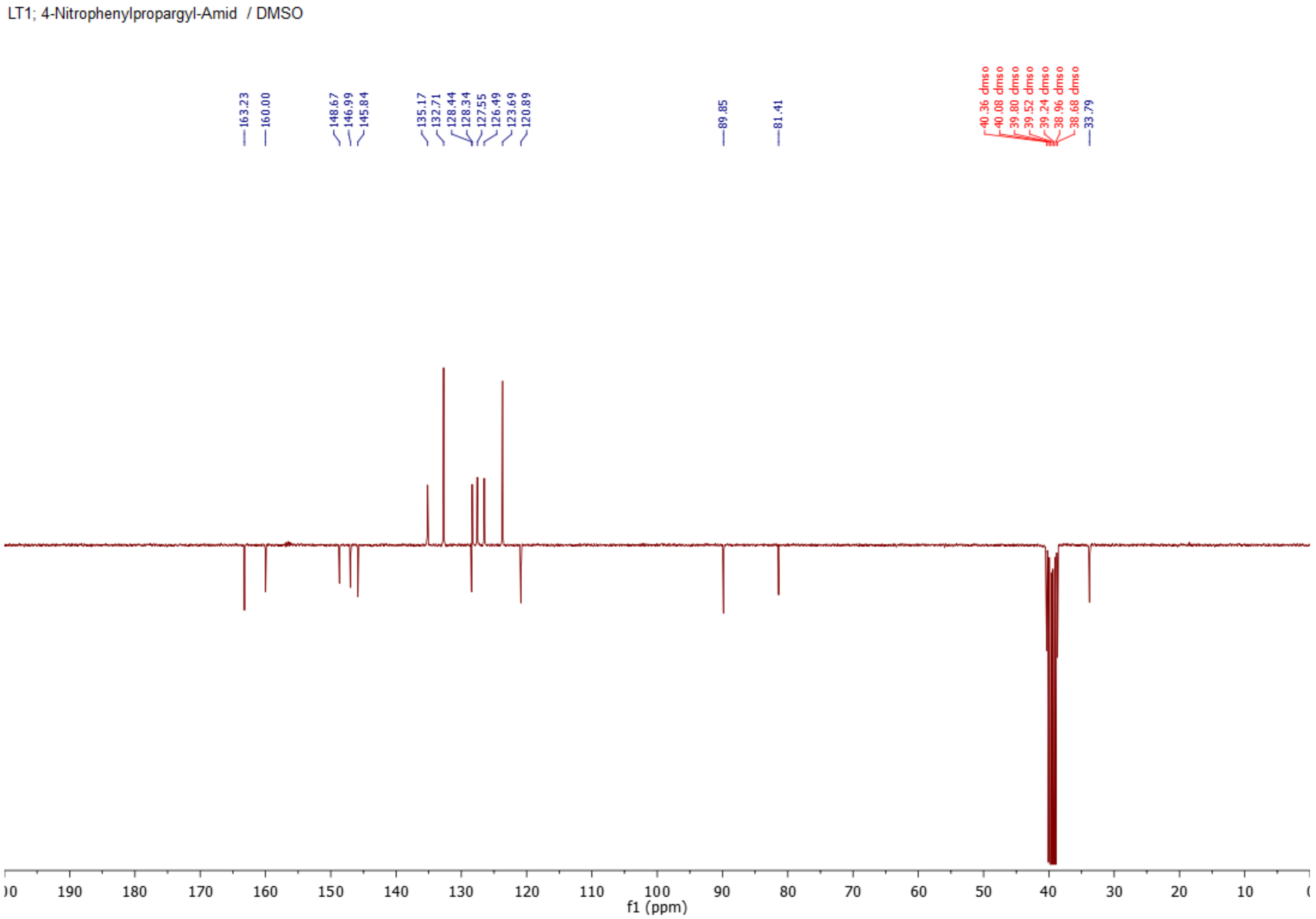

Figure S28. ^{13}C NMR spoectrum of 3-[3-(4-nitrophenyl)prop-2-yn-1-yl]-4-oxo-3,4-dihydroquinazoline-2-carboxamide (**7b**).

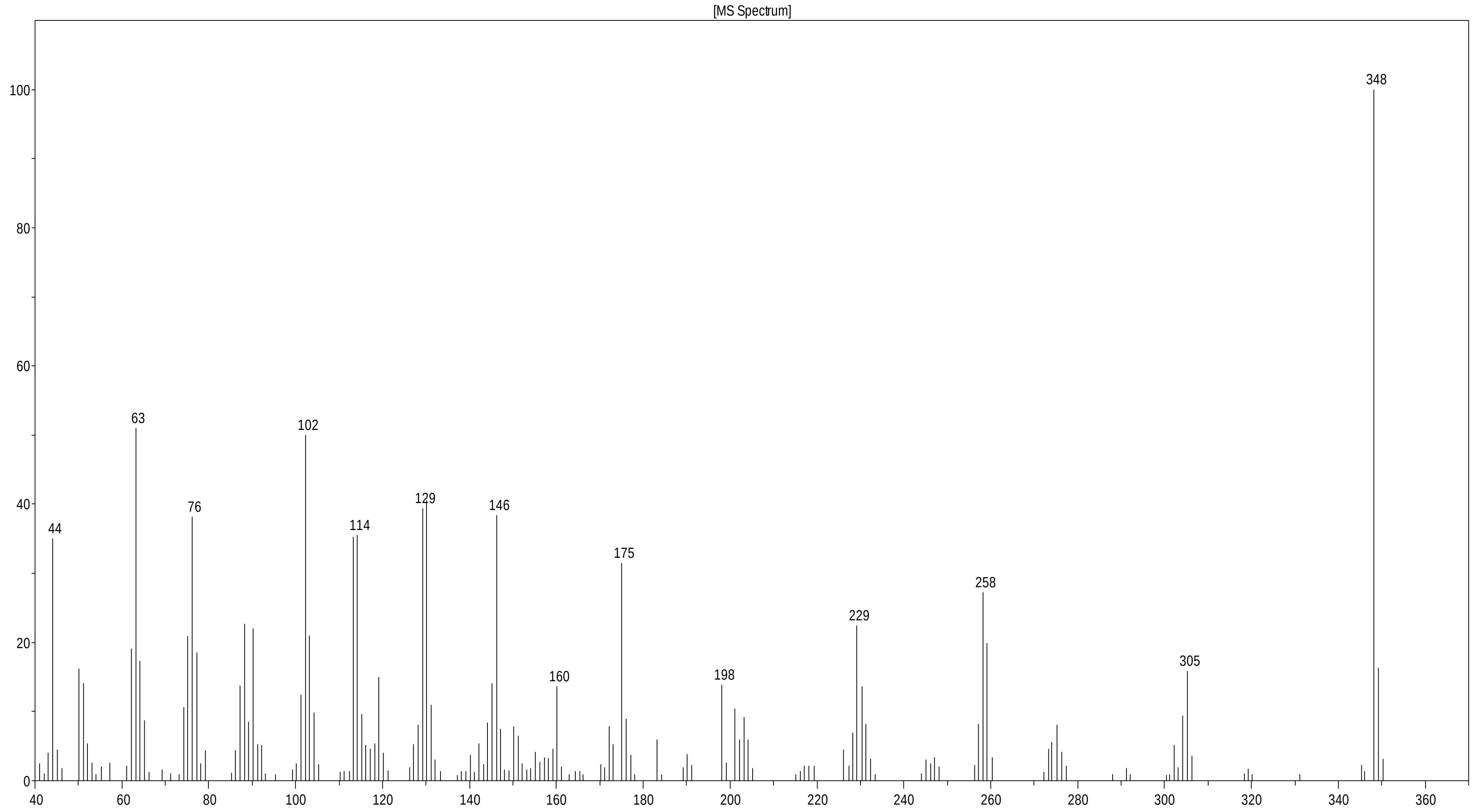

Figure S29. EI-MS of 3-[3-(4-nitrophenyl)prop-2-yn-1-yl]-4-oxo-3,4-dihydroquinazoline-2-carboxamide (**7b**).

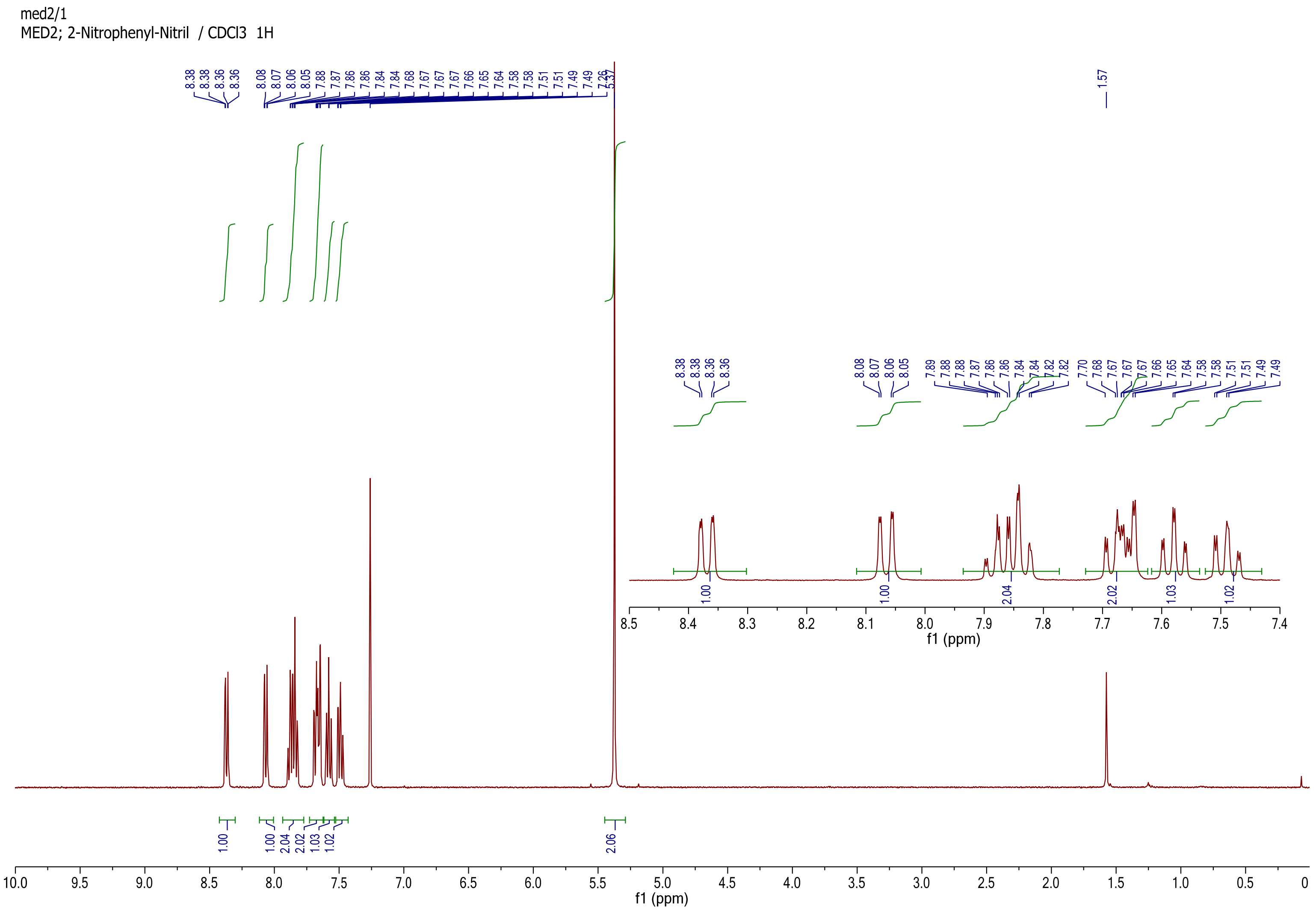

Figure S30. ^{1}H NMR spoectrum of 3-[3-(2-nitrophenyl)prop-2-yn-1-yl]-4-oxo-3,4-dihydroquinazoline-2-carbonitrile (**8a**).

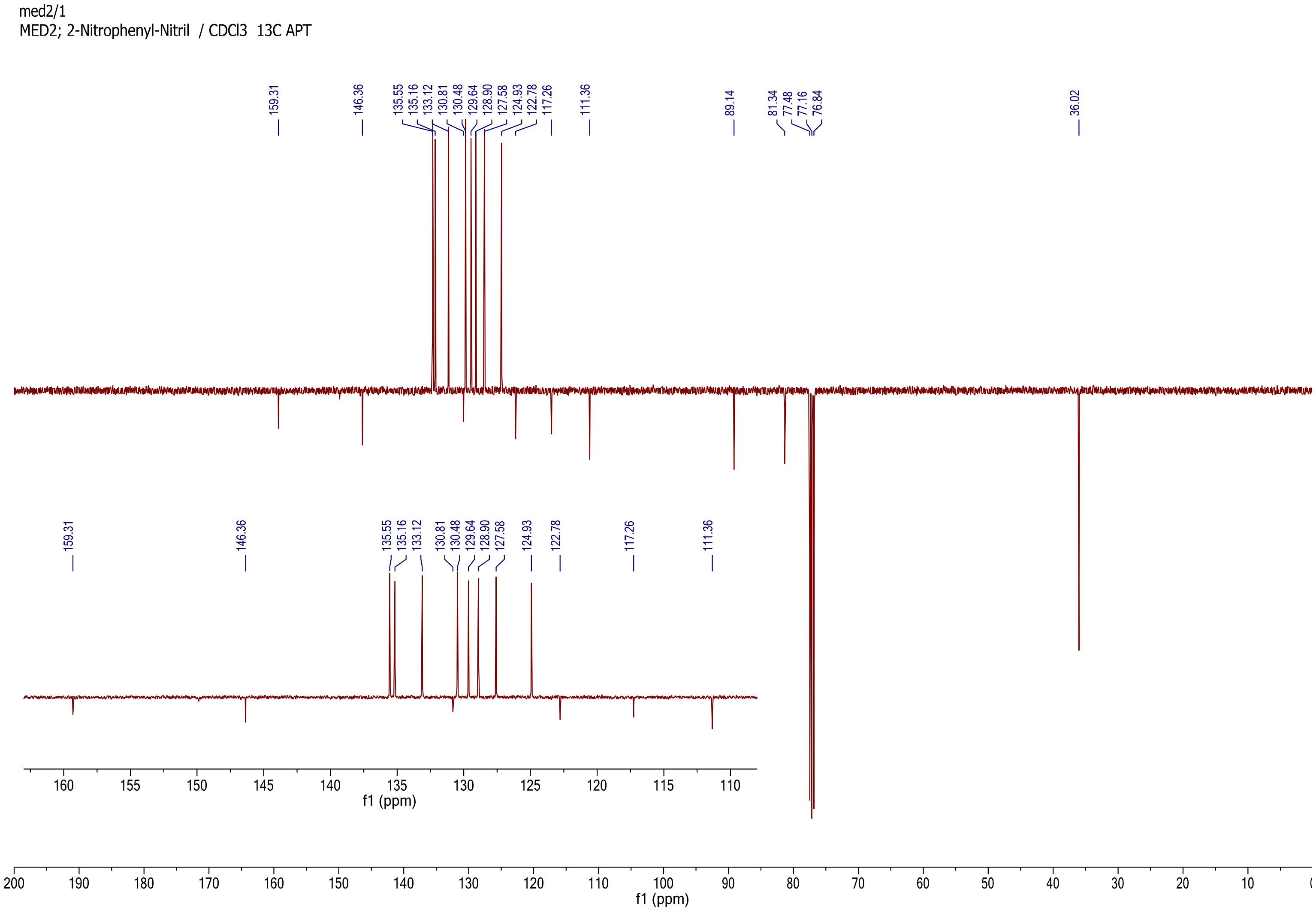

Figure S31. ^{1}H NMR spoectrum of 3-[3-(2-nitrophenyl)prop-2-yn-1-yl]-4-oxo-3,4-dihydroquinazoline-2-carbonitrile (**8a**).

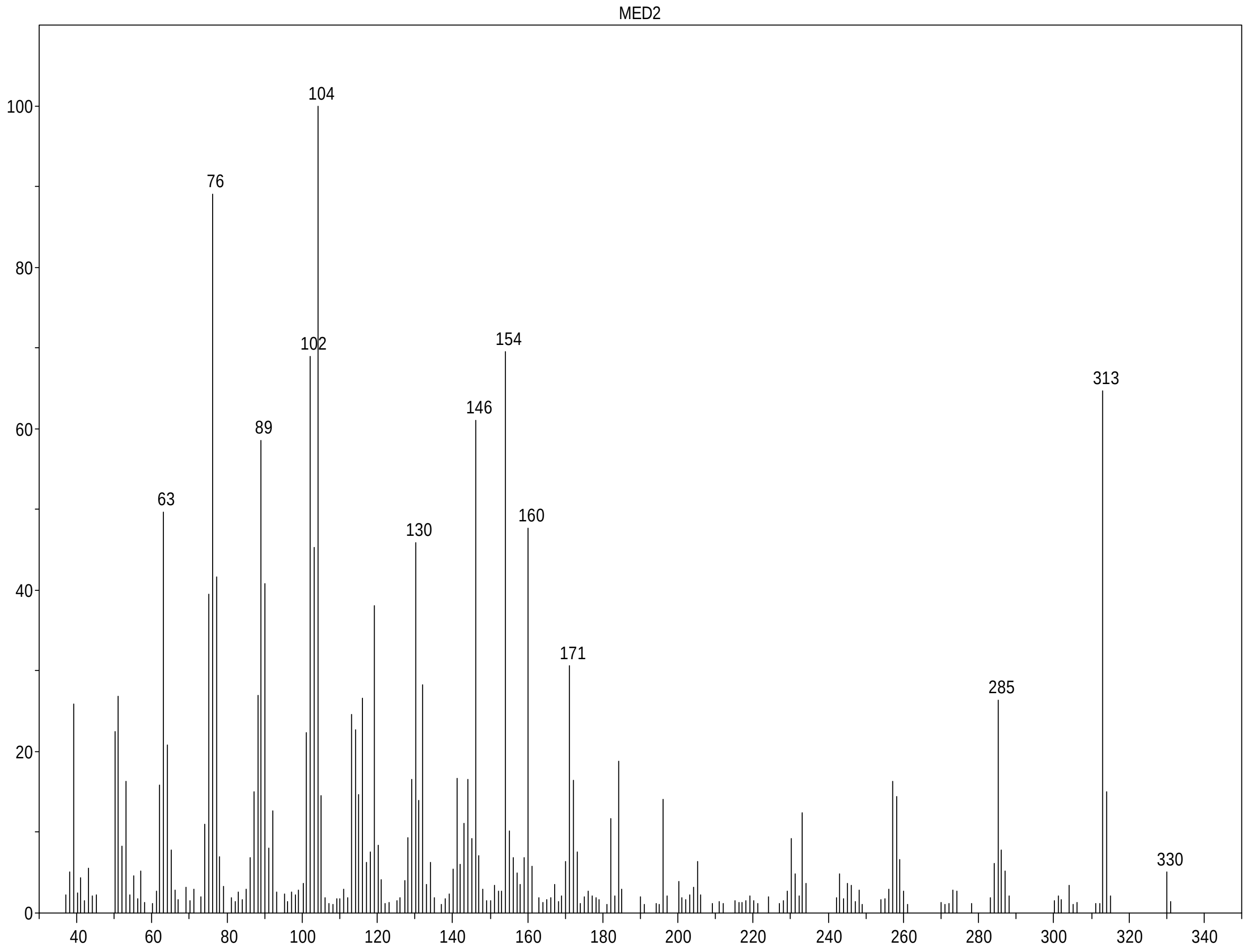

Figure S32. EI-MS of 3-[3-(2-nitrophenyl)prop-2-yn-1-yl]-4-oxo-3,4-dihydroquinazoline-2-carbonitrile (**8a**).

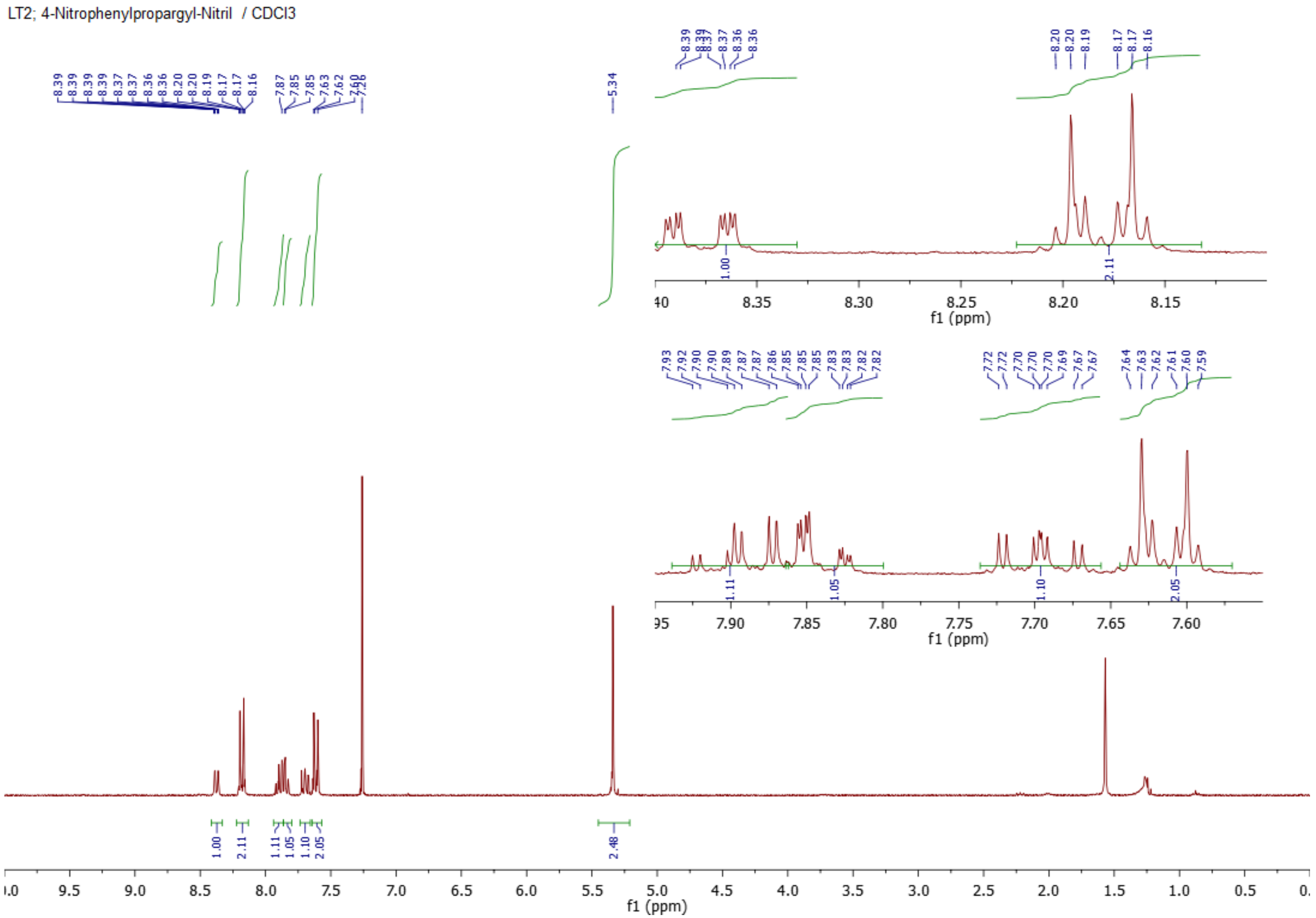

Figure S33. ^{1}H NMR spoectrum of 3-[3-(4-nitrophenyl)prop-2-yn-1-yl]-4-oxo-3,4-dihydroquinazoline-2-carbonitrile (**8b**).

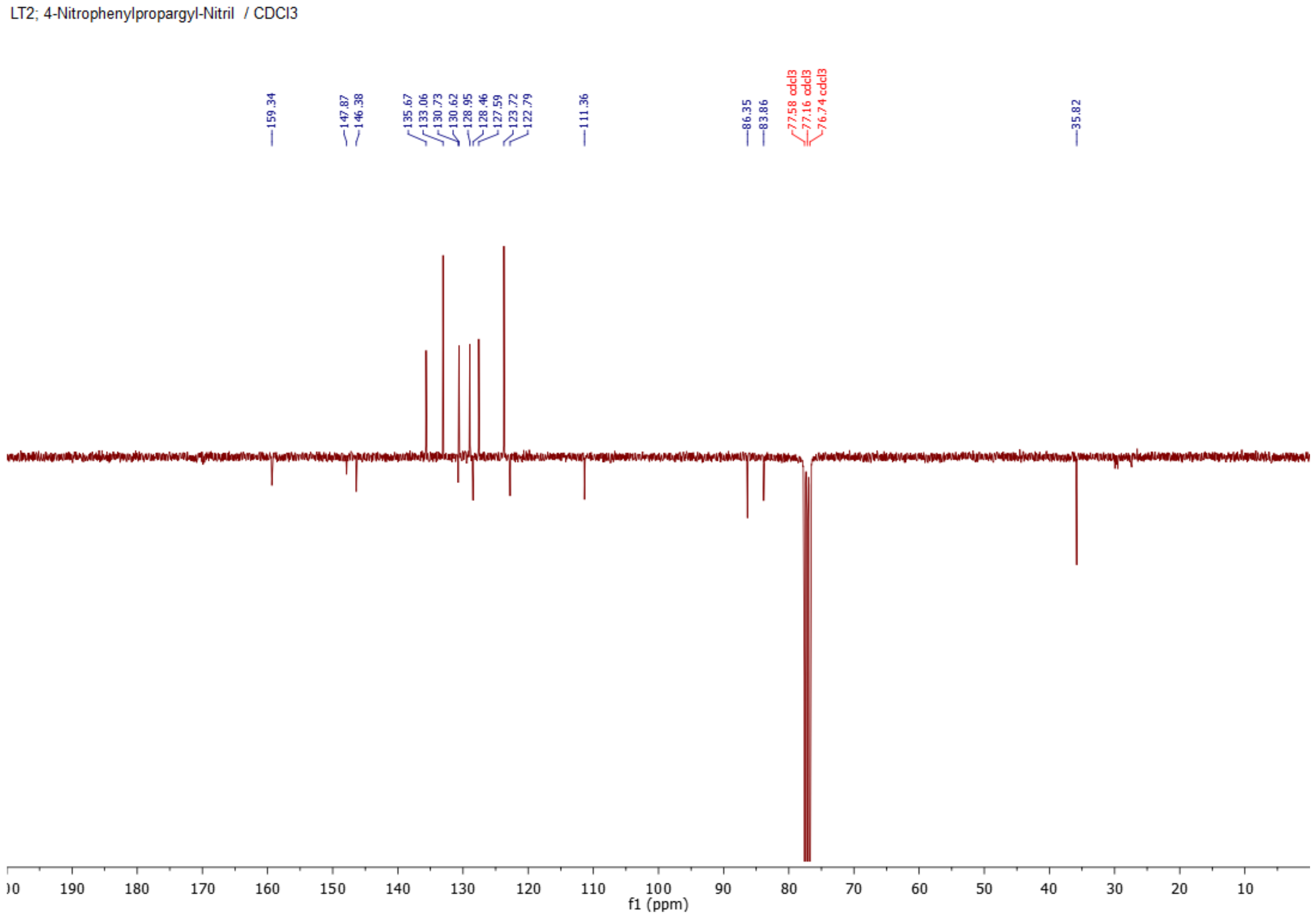

Figure S34. ^{13}C NMR spoectrum of 3-[3-(4-nitrophenyl)prop-2-yn-1-yl]-4-oxo-3,4-dihydroquinazoline-2-carbonitrile (**8b**).

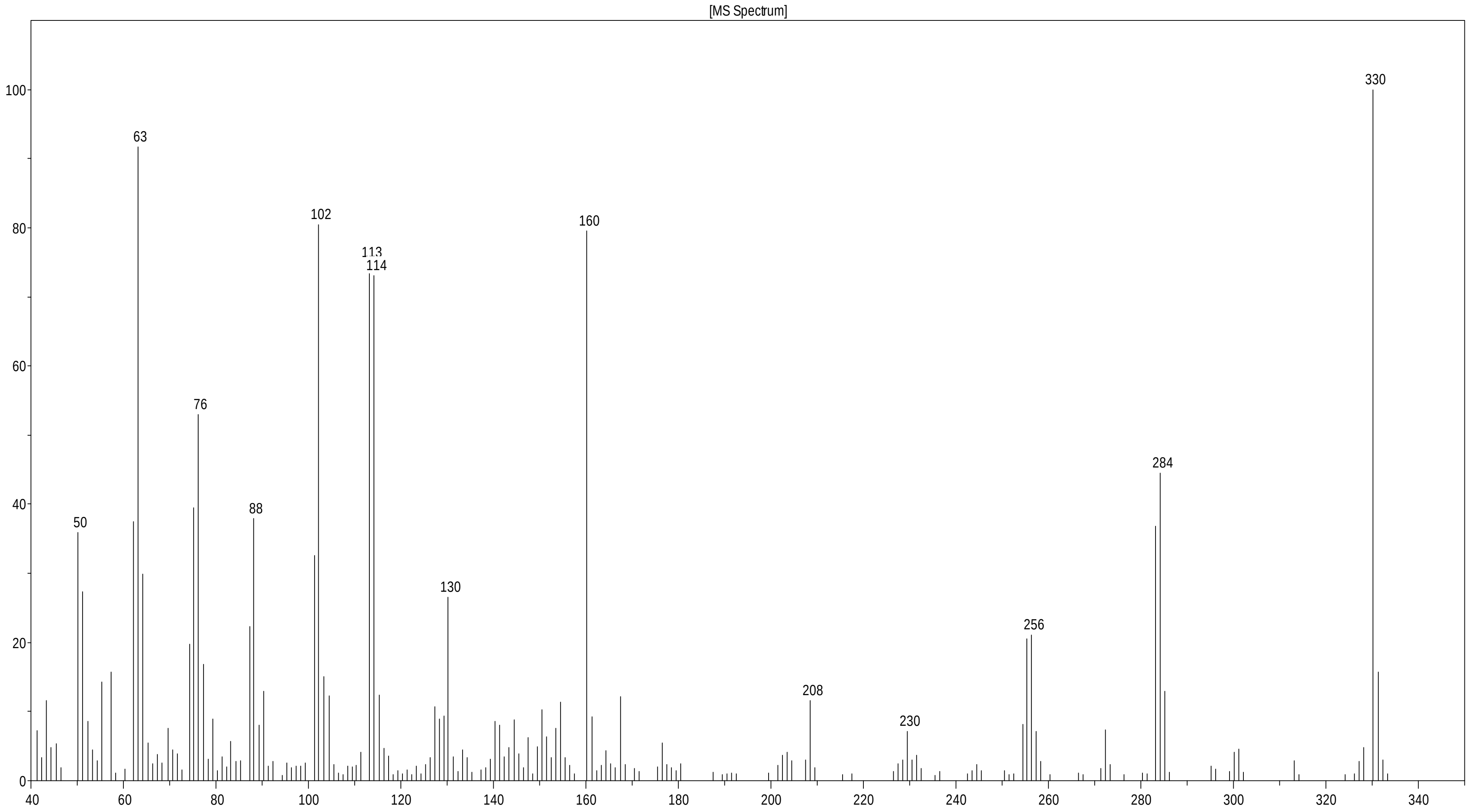

Figure S35. EI-MS of 3-[3-(4-nitrophenyl)prop-2-yn-1-yl]-4-oxo-3,4-dihydroquinazoline-2-carbonitrile (**8b**).

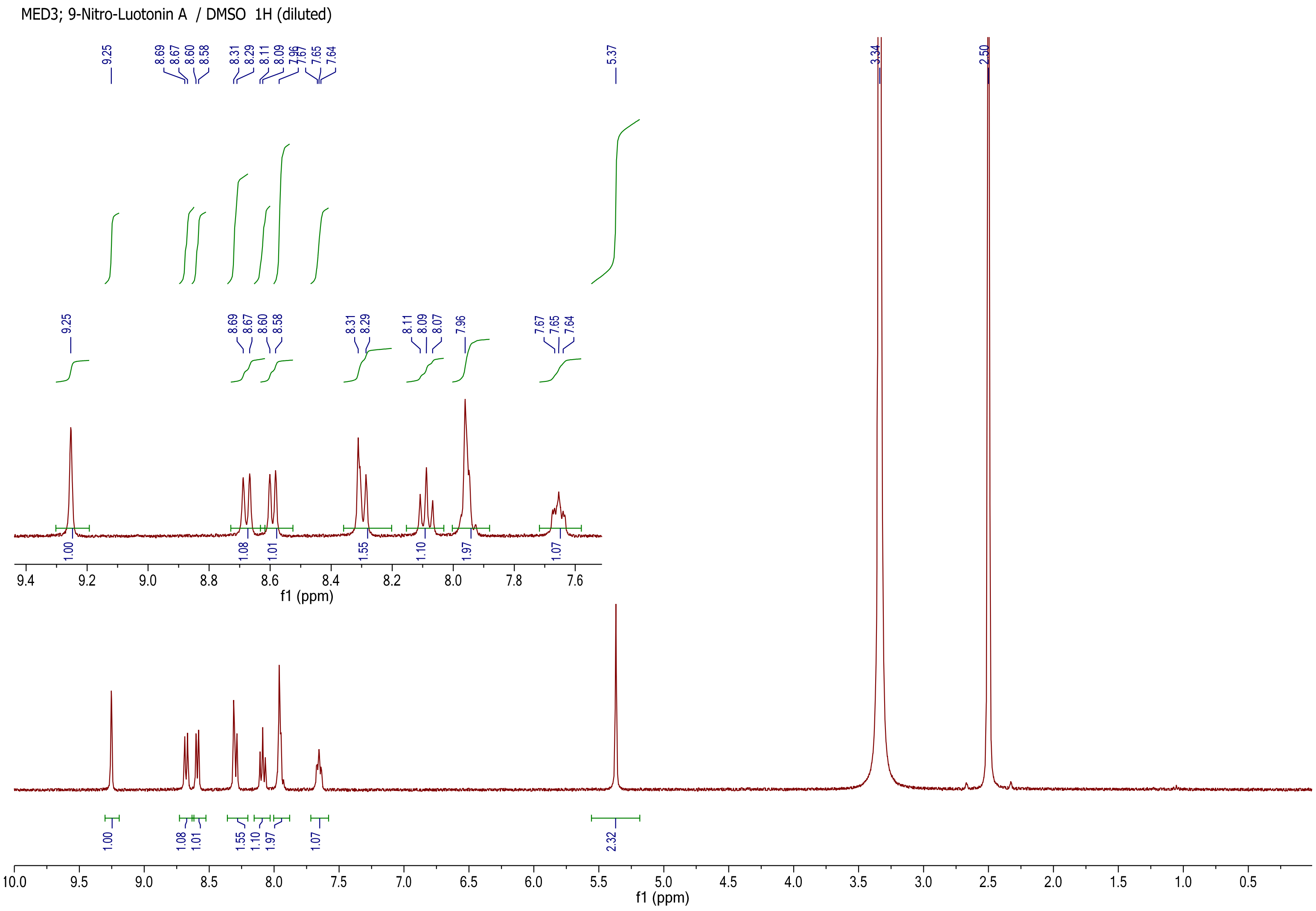

Figure S36. ^{1}H NMR spoectrum of 1-nitroquinolino[2',3':3,4]pyrrolo[2,1-*b*]quinazolin-11(13*H*)-one (**9a**).

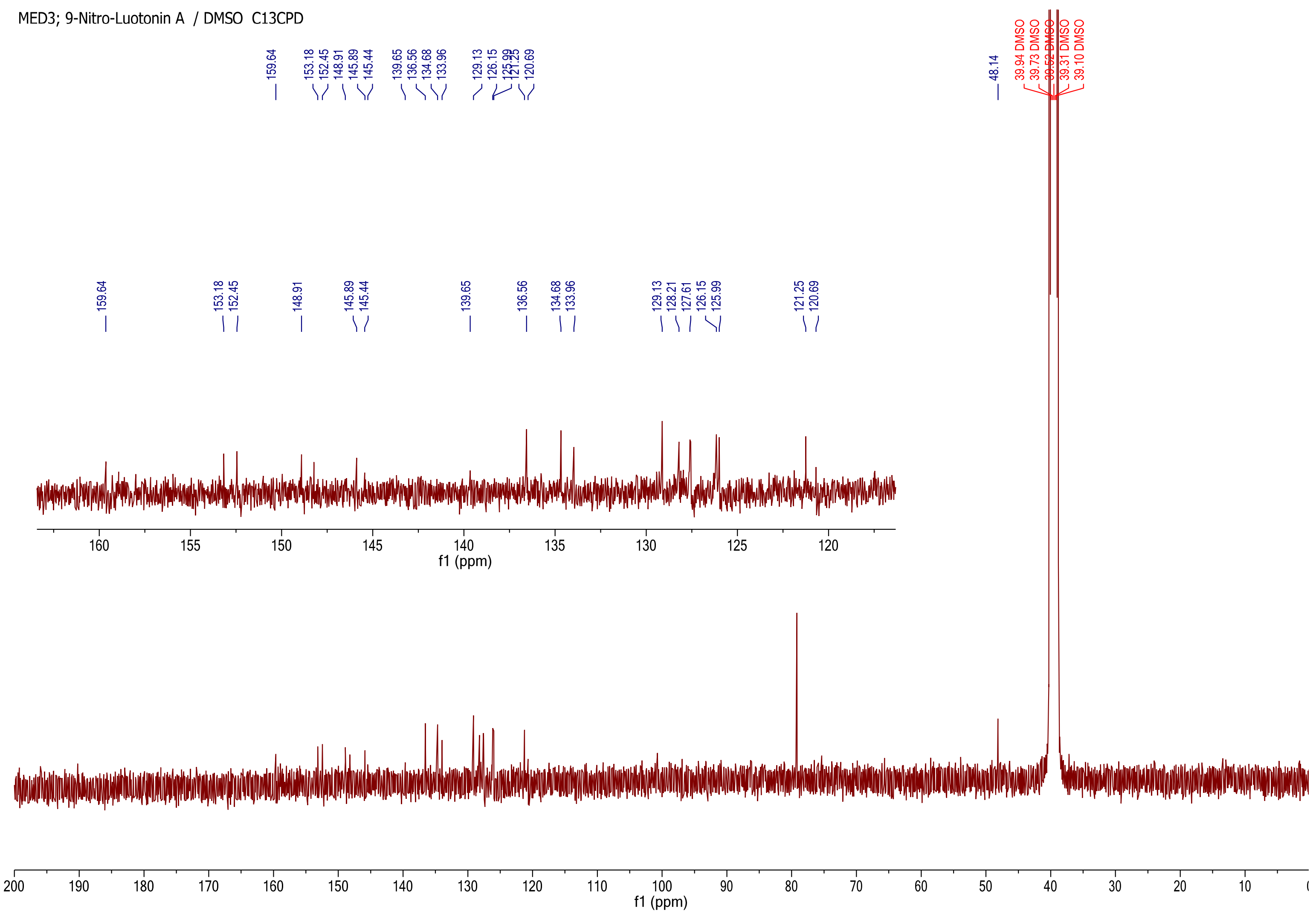

Figure S37. ^{13}C NMR spoectrum of 1-nitroquinolino[2',3':3,4]pyrrolo[2,1-*b*]quinazolin-11(13*H*)-one (**9a**).

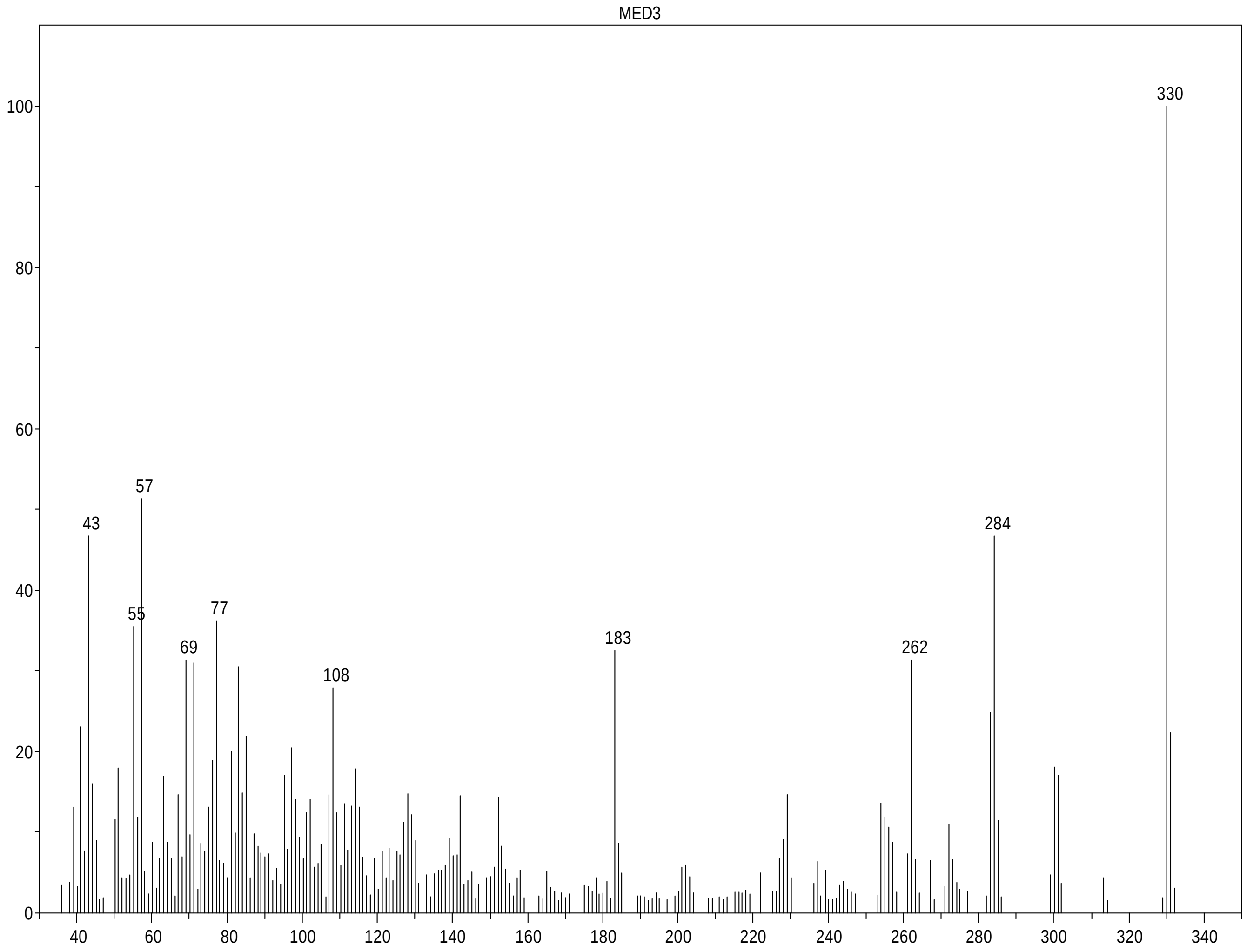

Figure S38. EI-MS of 1-nitroquinolino[2',3':3,4]pyrrolo[2,1-*b*]quinazolin-11(13*H*)-one (**9a**).

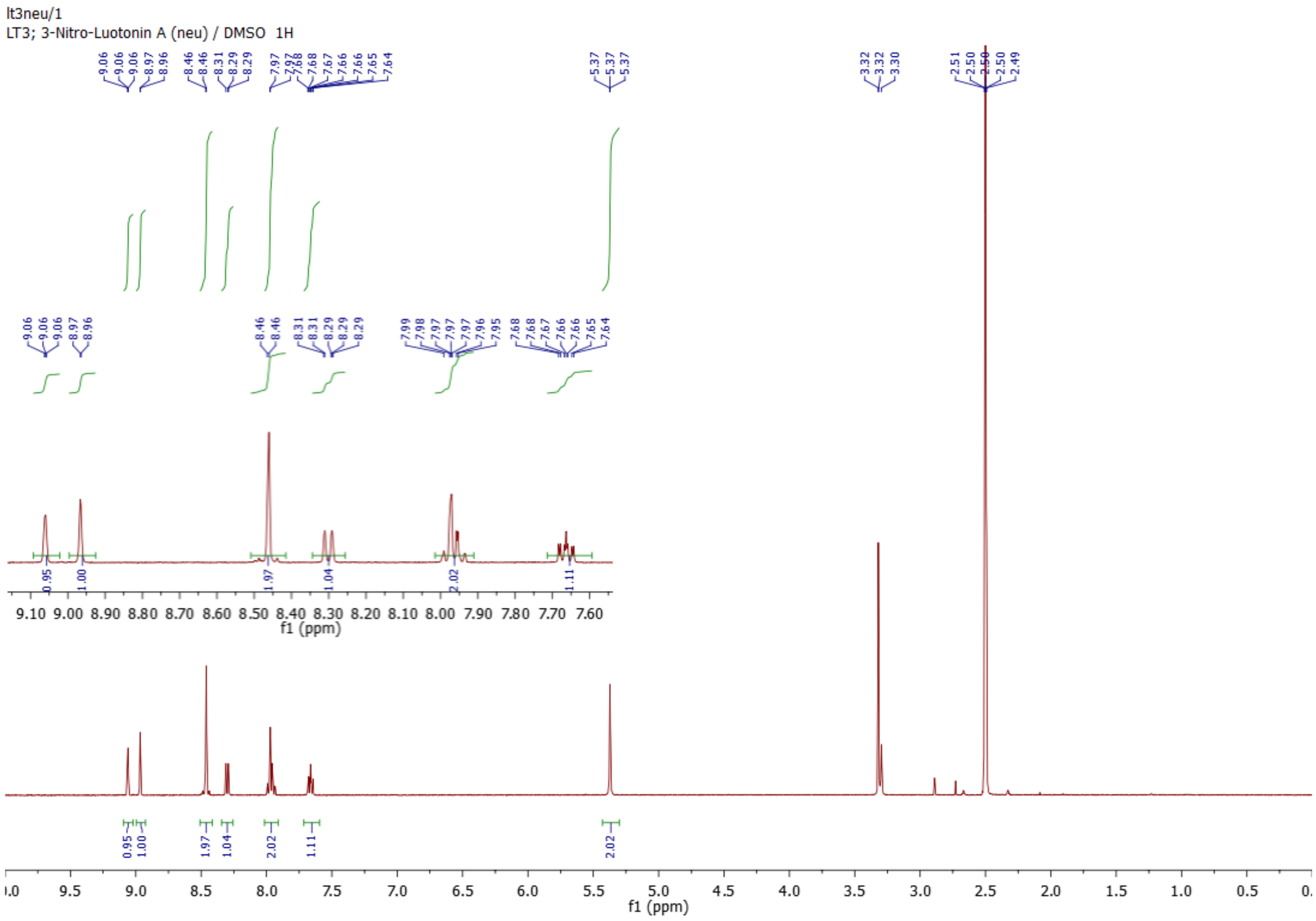

Figure S39. ^{1}H NMR spoectrum of 3-nitroquinolino[2',3':3,4]pyrrolo[2,1-*b*]quinazolin-11(13*H*)-one (**9b**).

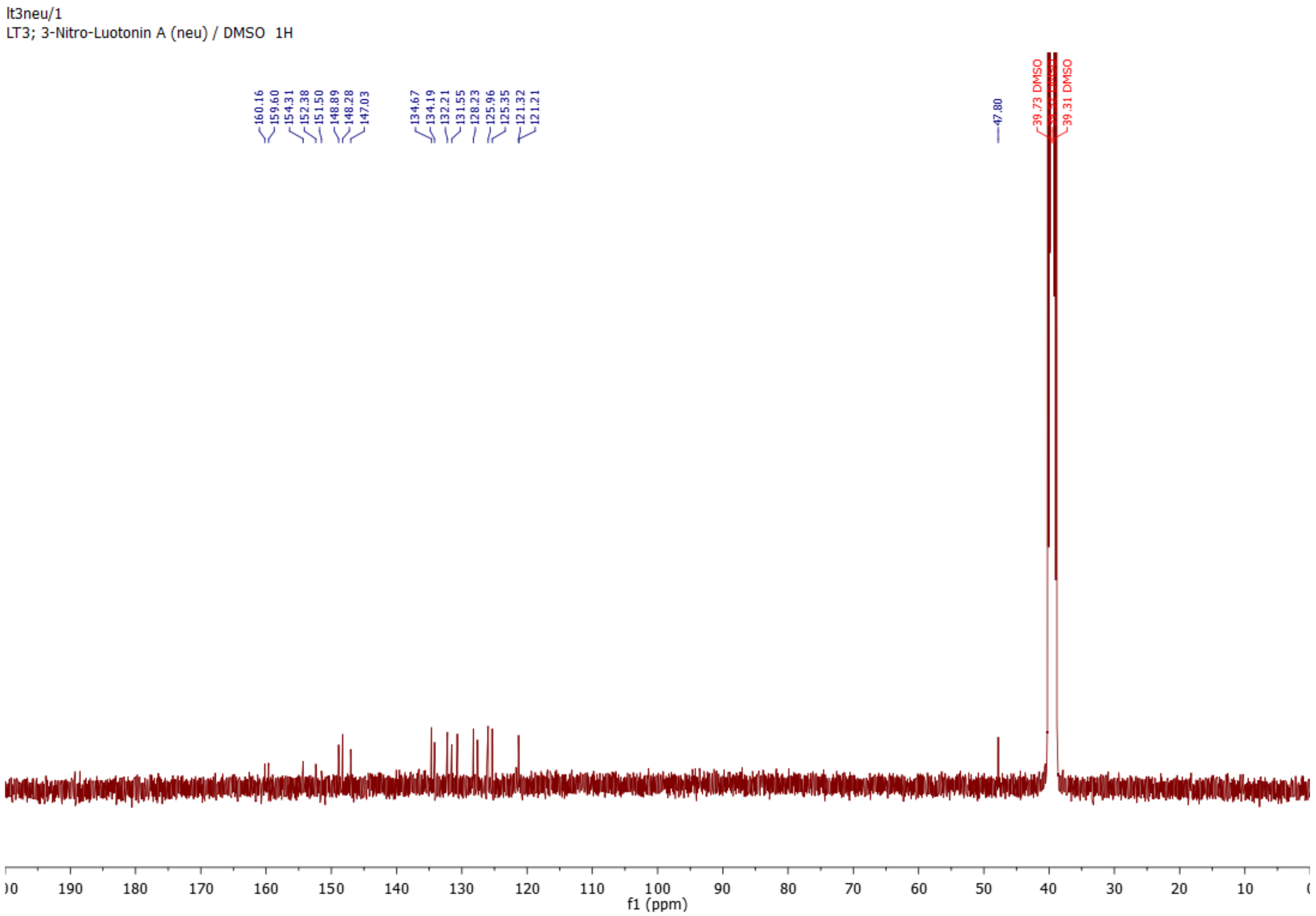

Figure S40. ^{13}C NMR spoectrum of 3-nitroquinolino[2',3':3,4]pyrrolo[2,1-*b*]quinazolin-11(13*H*)-one (**9b**).

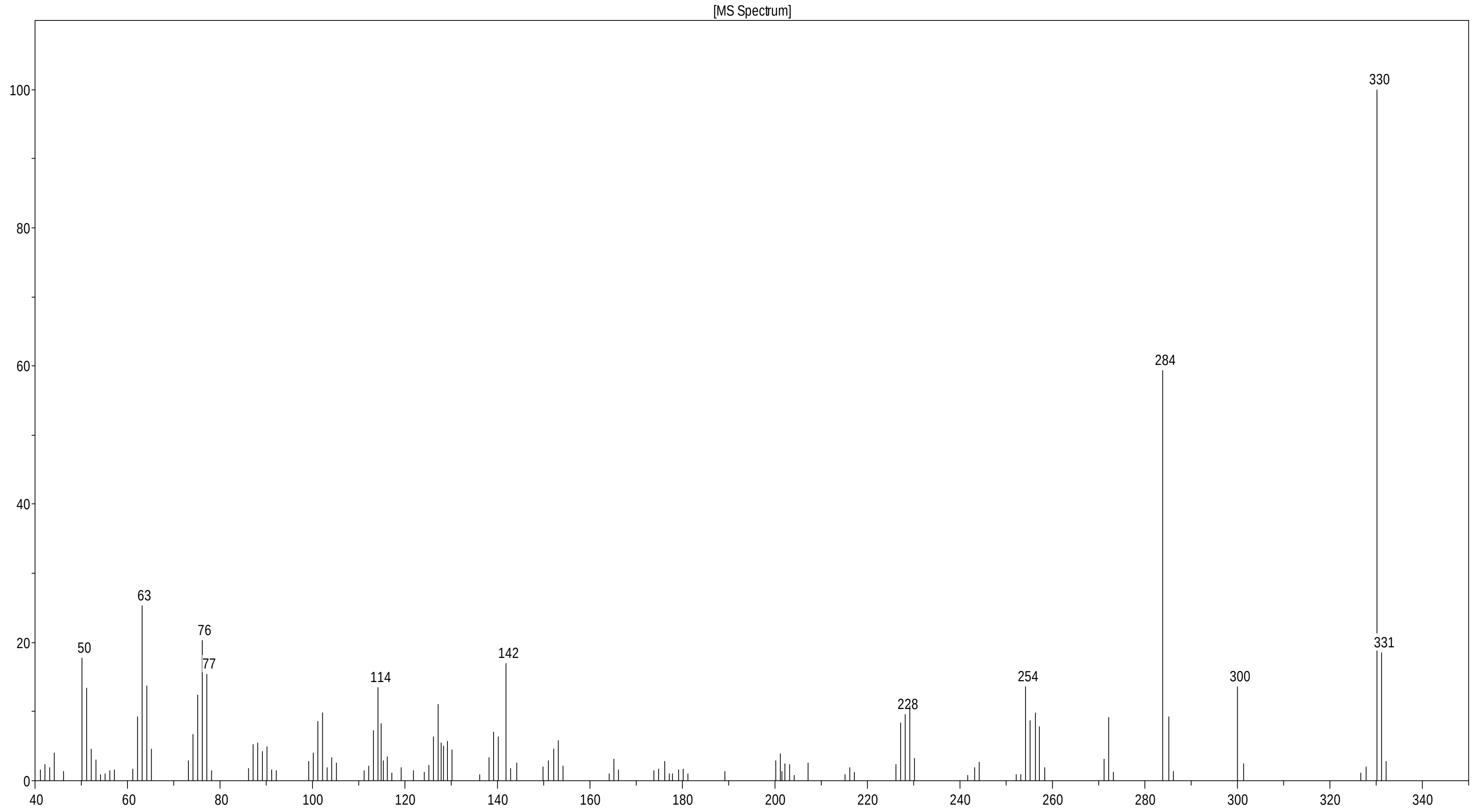

Figure S41. EI-MS of 3-nitroquinolino[2',3':3,4]pyrrolo[2,1-*b*]quinazolin-11(13*H*)-one (**9b**).

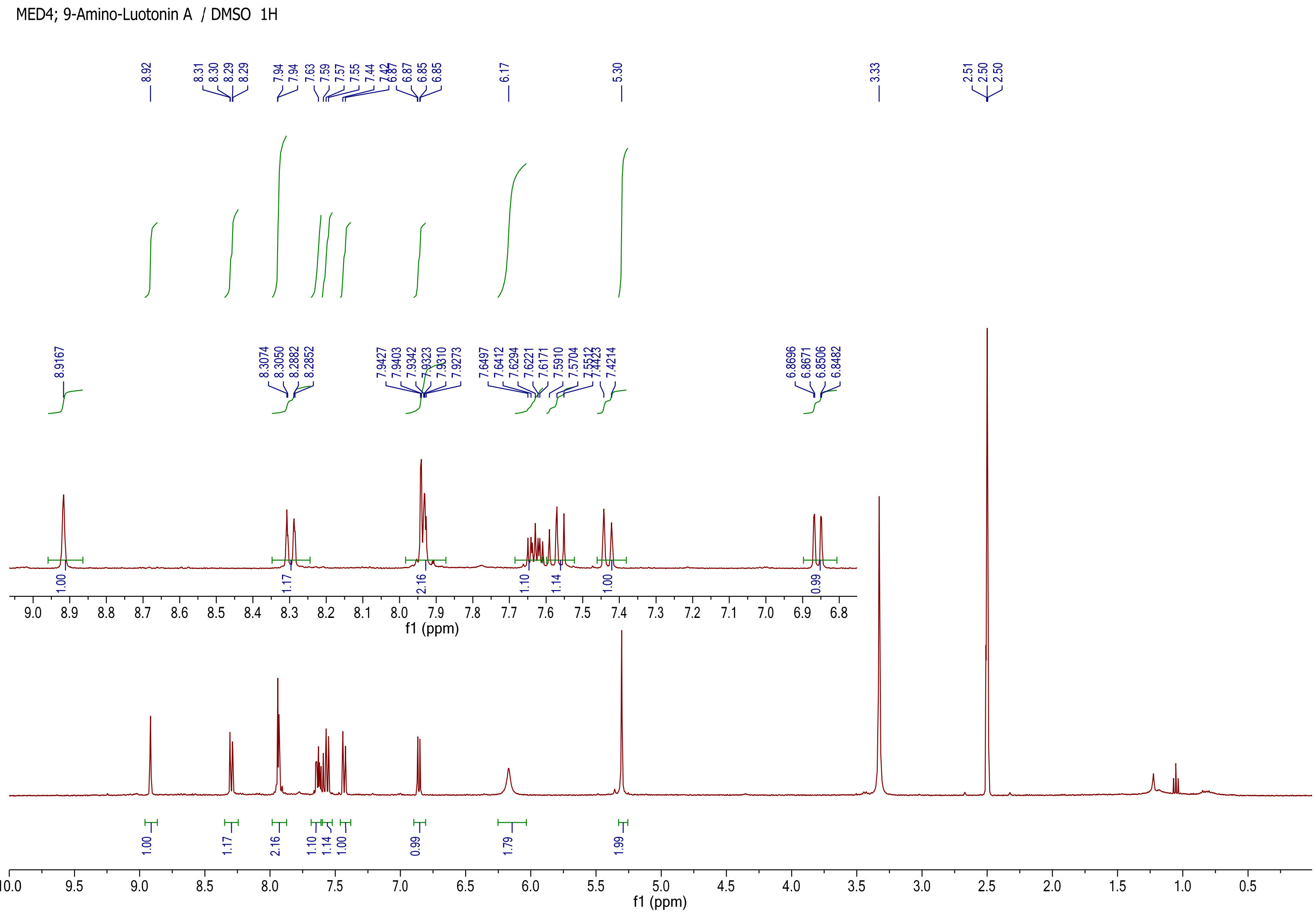

Figure S42. ^{1}H NMR spoectrum of 1-aminoquinolino[2',3':3,4]pyrrolo[2,1-*b*]quinazolin-11(13*H*)-one (**10a**).

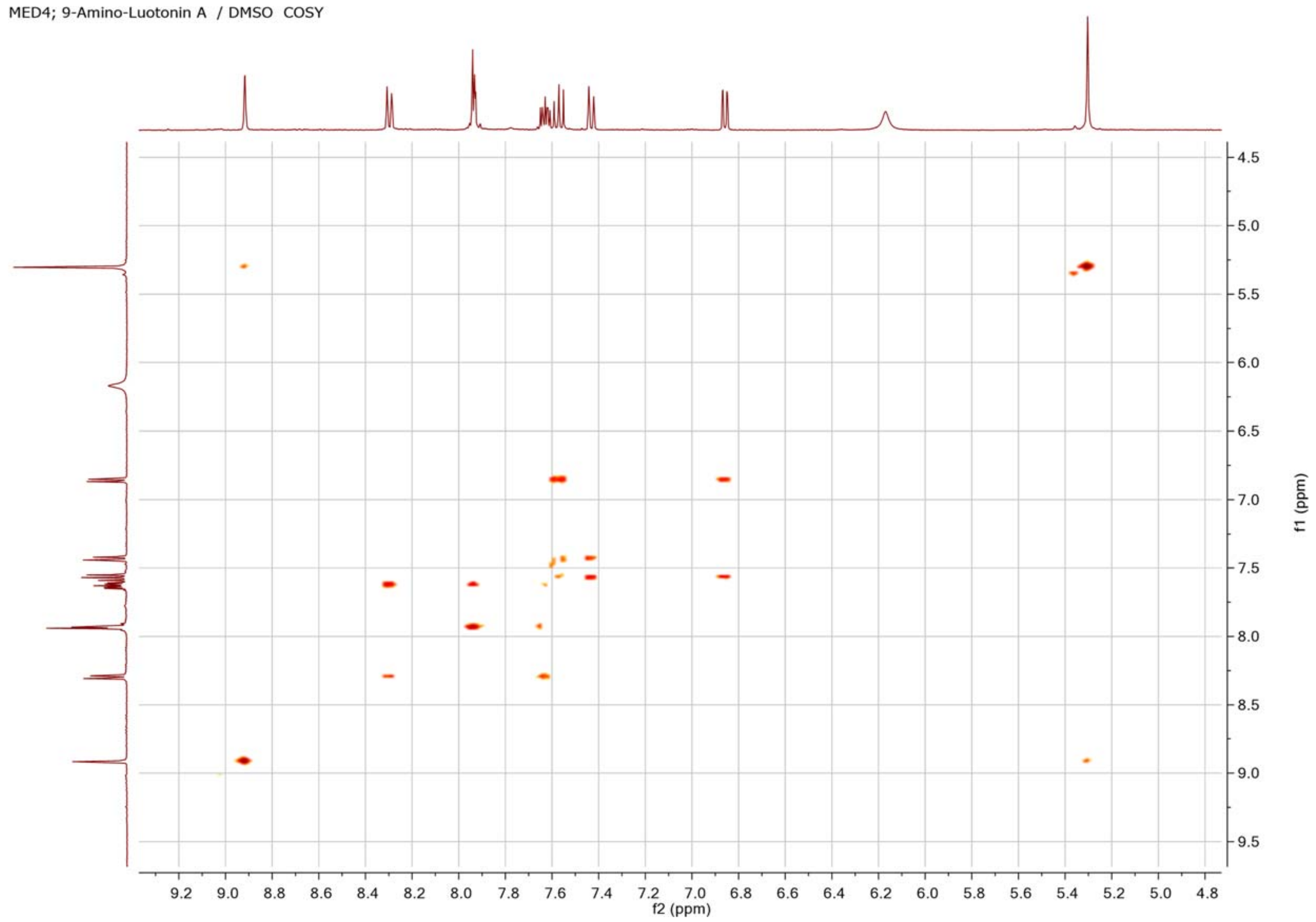

Figure S43. COSY spoectrum of 1-aminoquinolino[2',3':3,4]pyrrolo[2,1-*b*]quinazolin-11(13*H*)-one (**10a**).

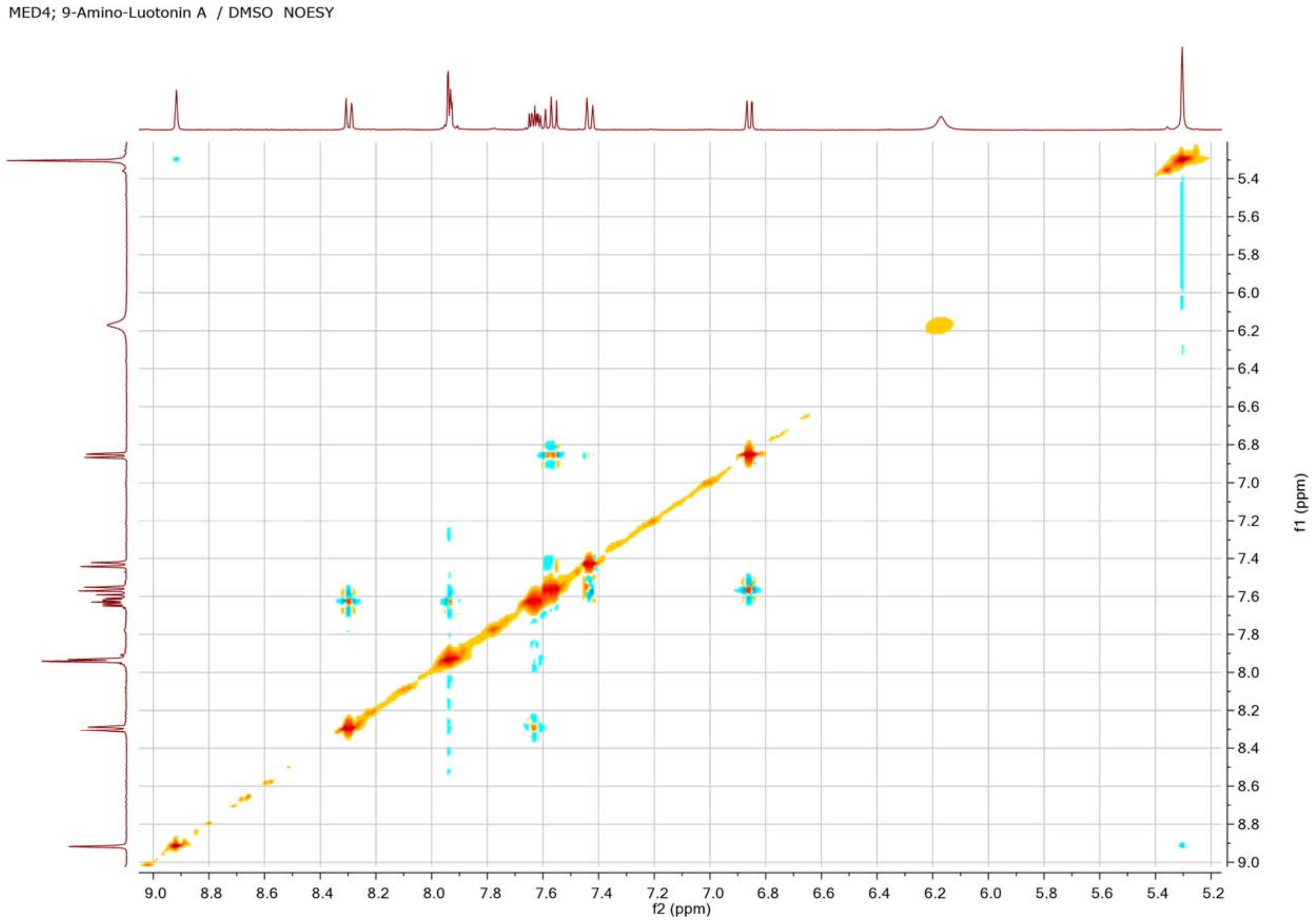

Figure S44. NOESY spoectrum of 1-aminoquinolino[2',3':3,4]pyrrolo[2,1-*b*]quinazolin-11(13*H*)-one (**10a**).

MED4; 9-Amino-Luotonin A / DMSO C13APT

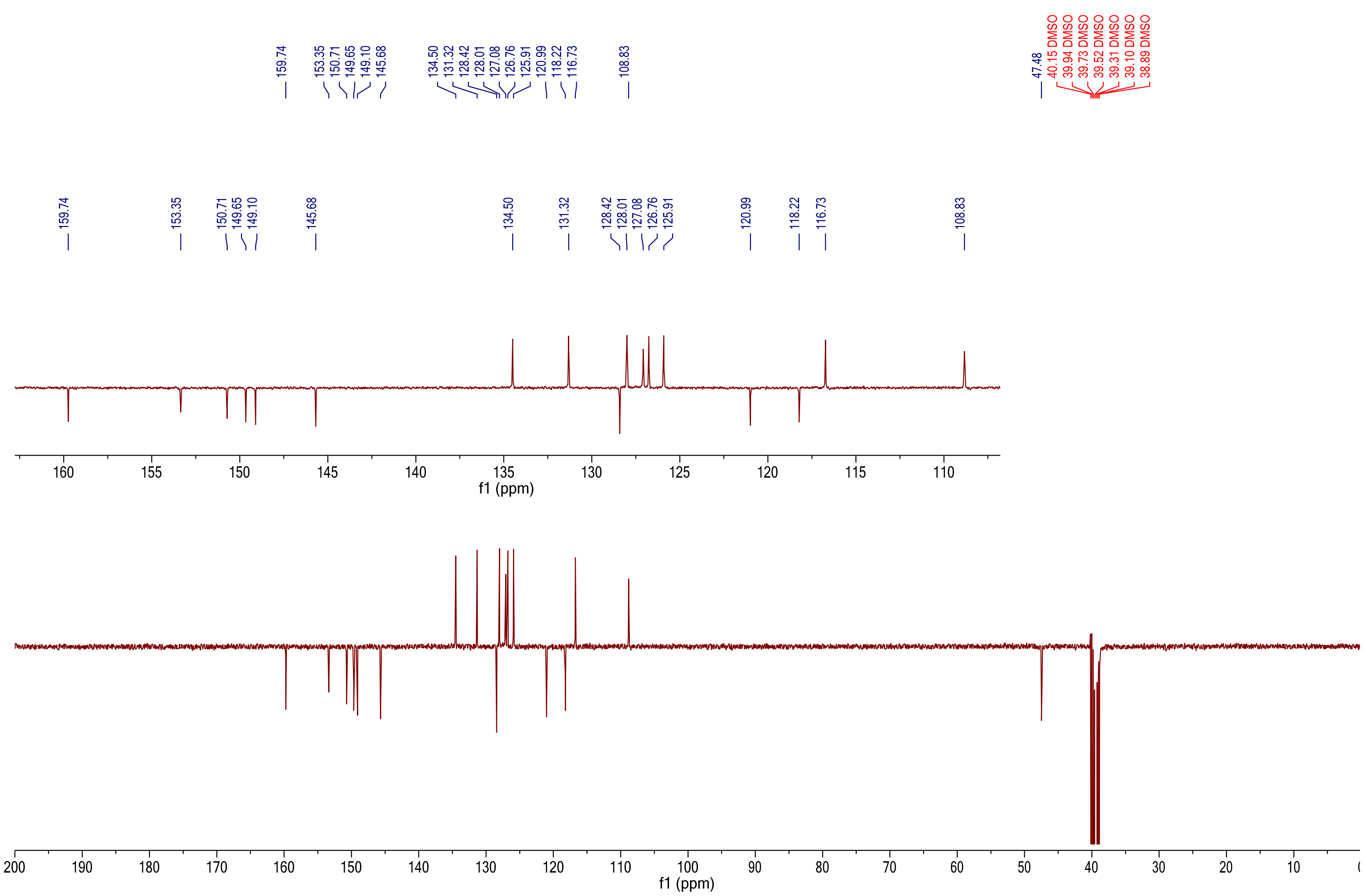

Figure S45. ^{13}C NMR spoectrum of 1-aminoquinolino[2',3':3,4]pyrrolo[2,1-*b*]quinazolin-11(13*H*)-one (**10a**).

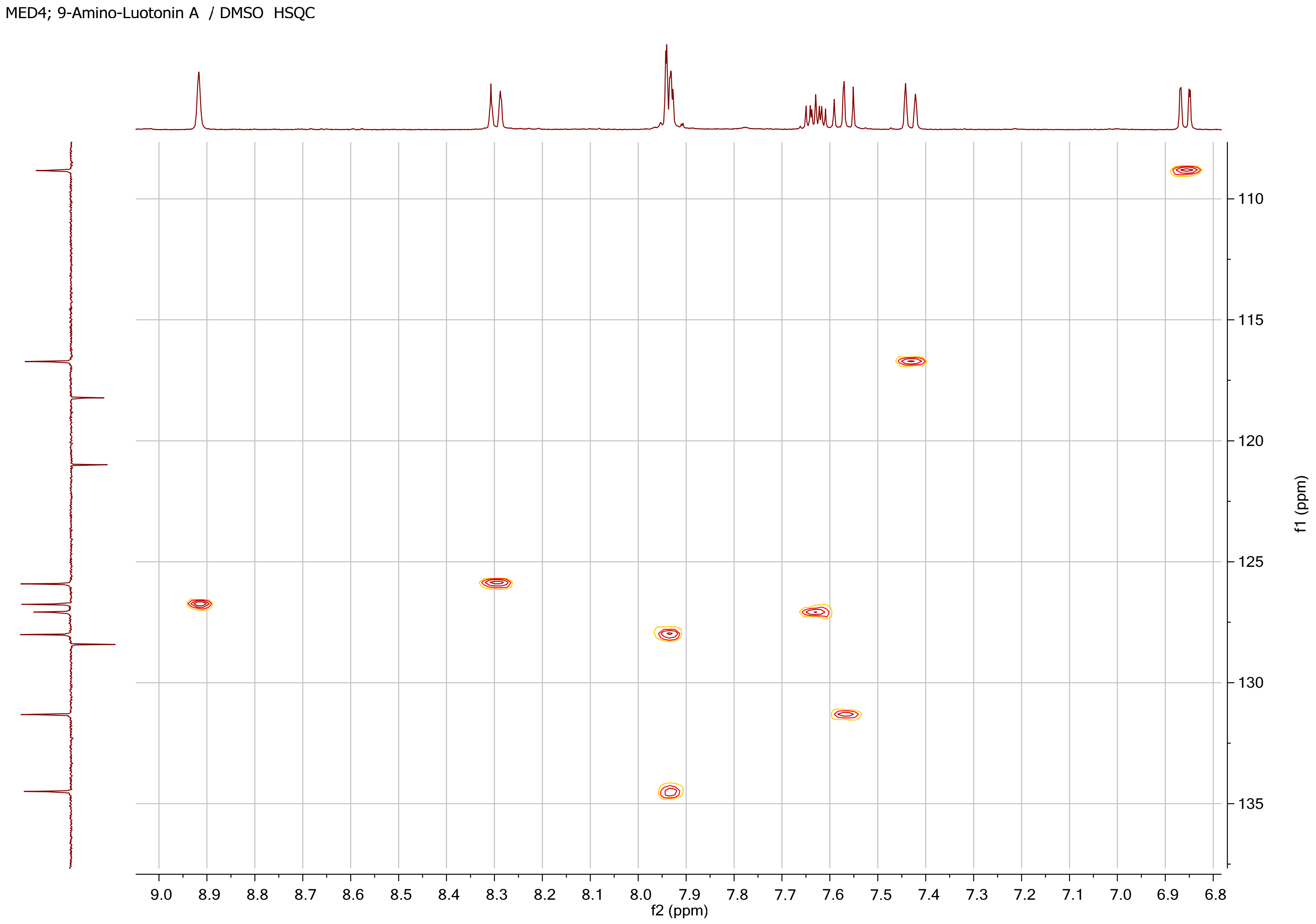

Figure S46. HSQC spoectrum of 1-aminoquinolino[2',3':3,4]pyrrolo[2,1-*b*]quinazolin-11(13*H*)-one (**10a**).

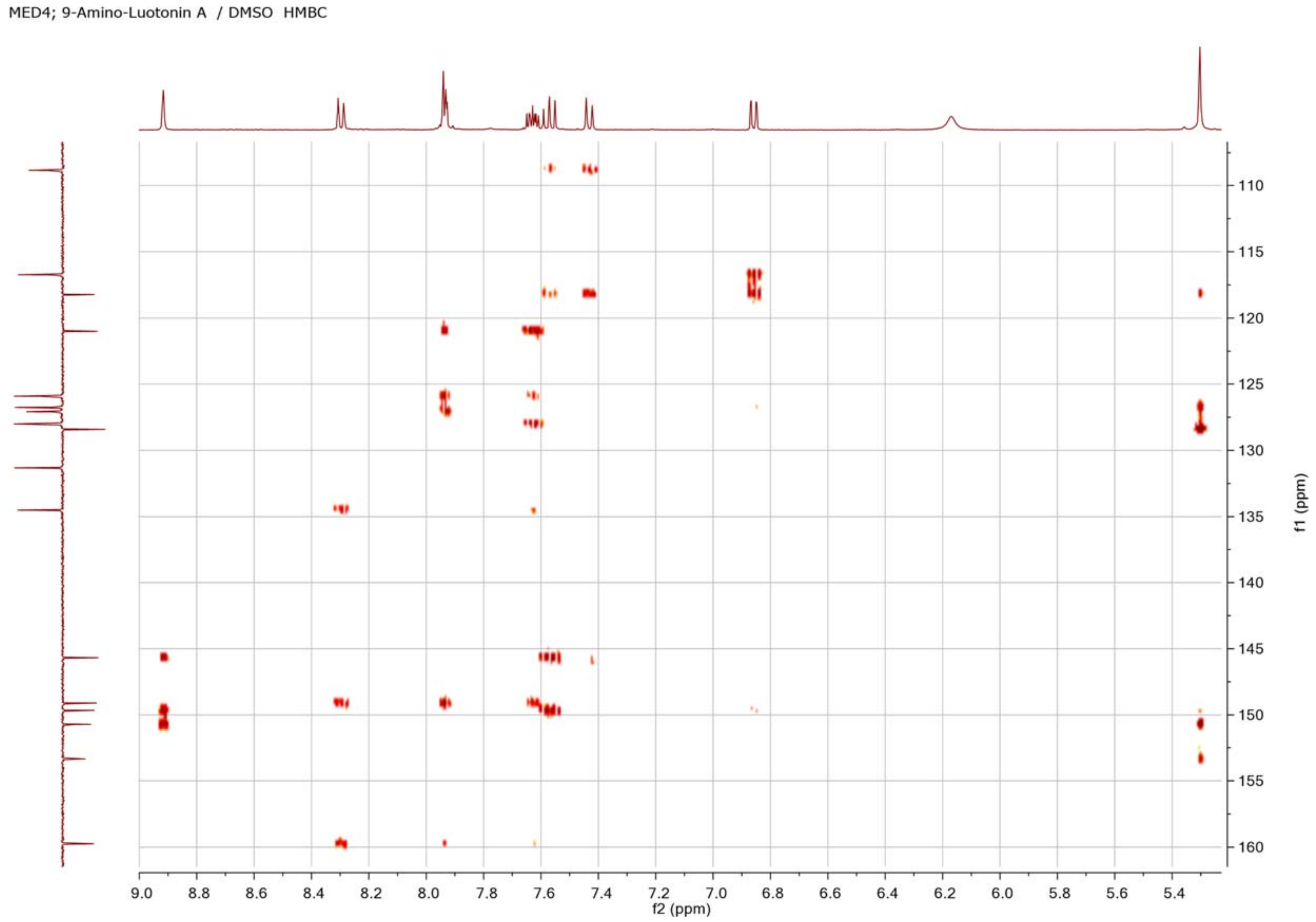

Figure S47. HMBC spoectrum of 1-aminoquinolino[2',3':3,4]pyrrolo[2,1-*b*]quinazolin-11(13*H*)-one (**10a**).

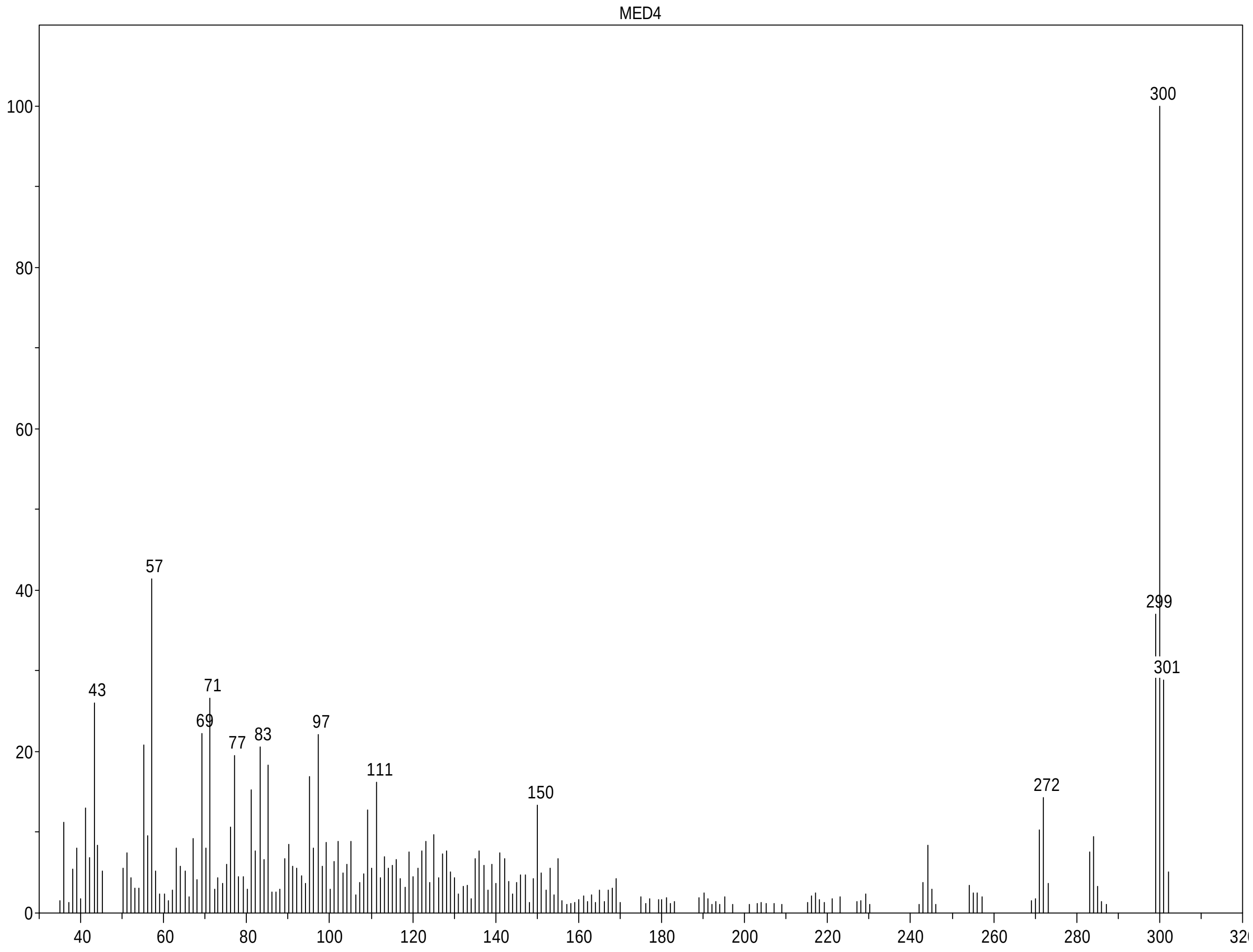

Figure S48. EI-MS of 1-aminoquinolino[2',3':3,4]pyrrolo[2,1-*b*]quinazolin-11(13*H*)-one (**10a**).

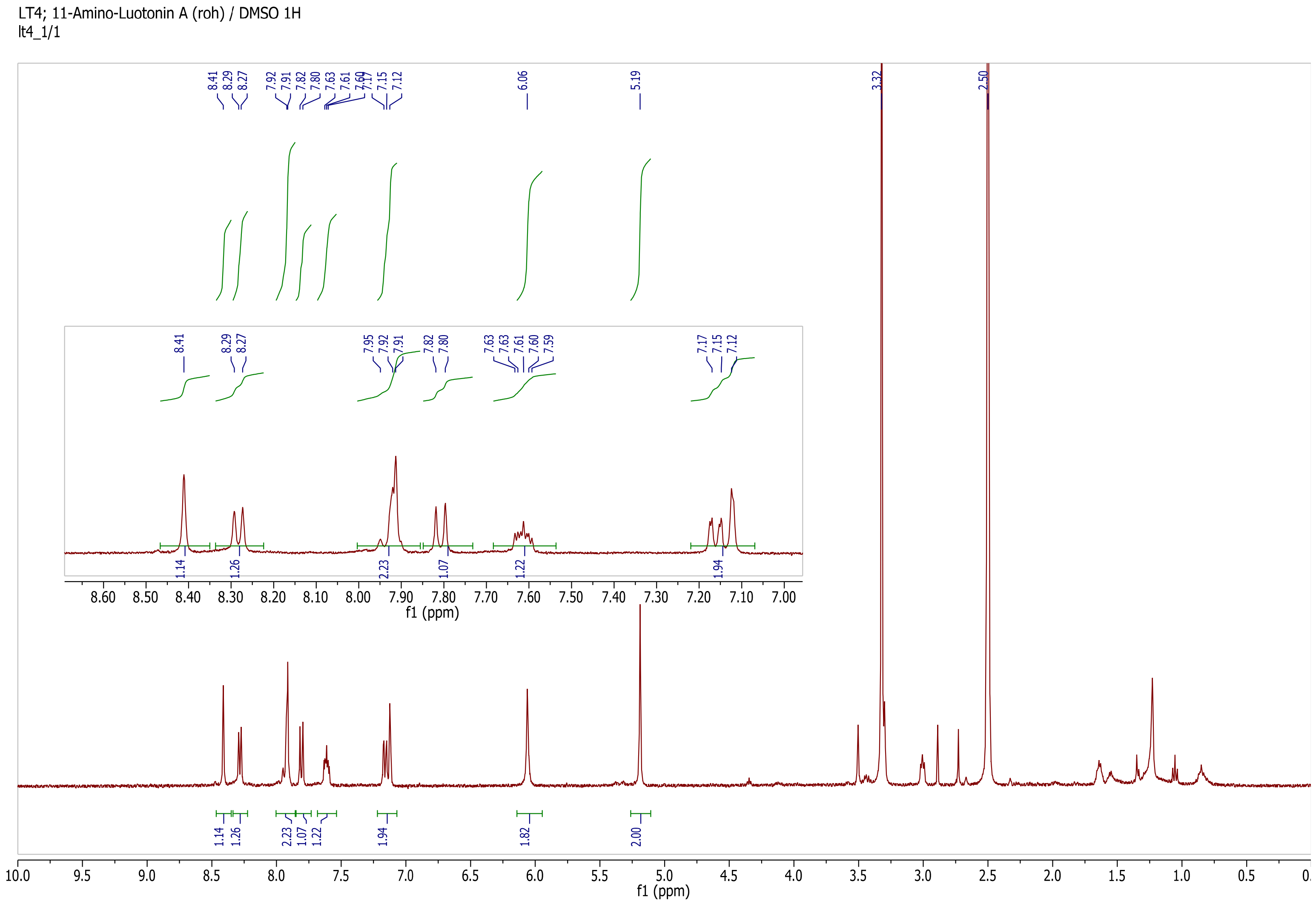

Figure S49. ^{1}H NMR spoectrum of 3-aminoquinolino[2',3':3,4]pyrrolo[2,1-*b*]quinazolin-11(13*H*)-one (**10b**).

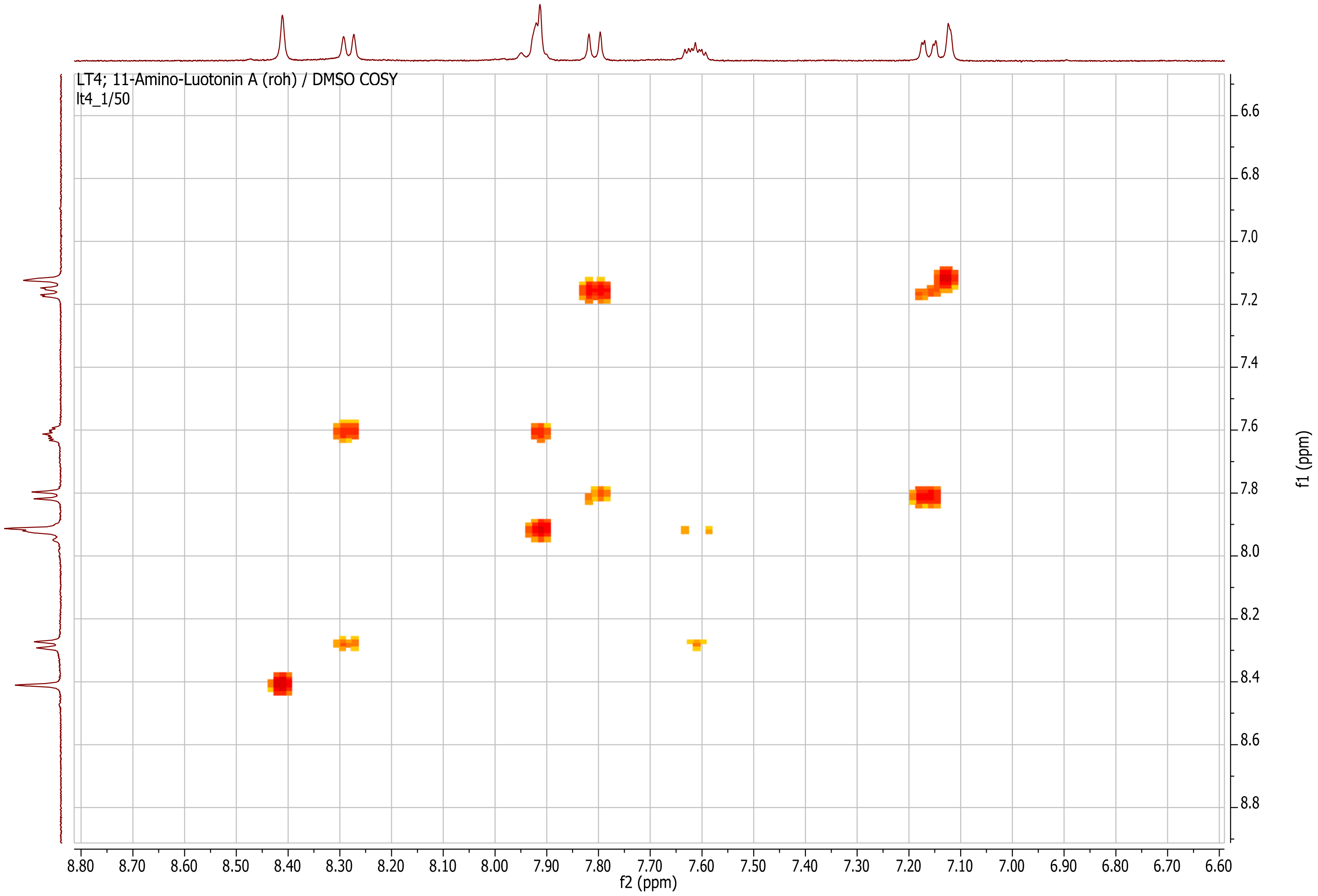

Figure S50. COSY spoectrum of 3-aminoquinolino[2',3':3,4]pyrrolo[2,1-*b*]quinazolin-11(13*H*)-one (**10b**).

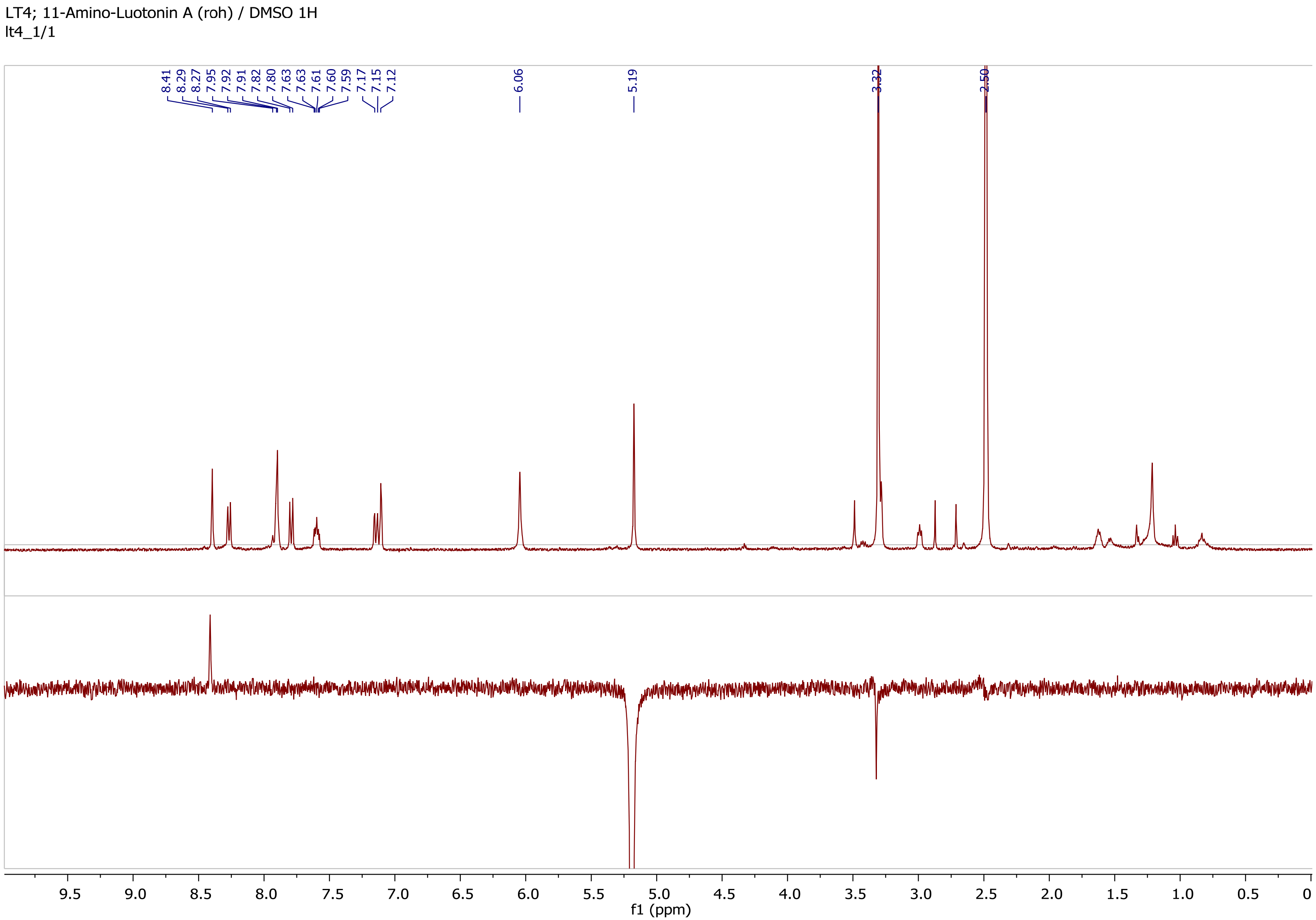

Figure S51. NOE difference spoectrum #1 of 3-aminoquinolino[2',3':3,4]pyrrolo[2,1-*b*]quinazolin-11(13*H*)-one (**10b**).

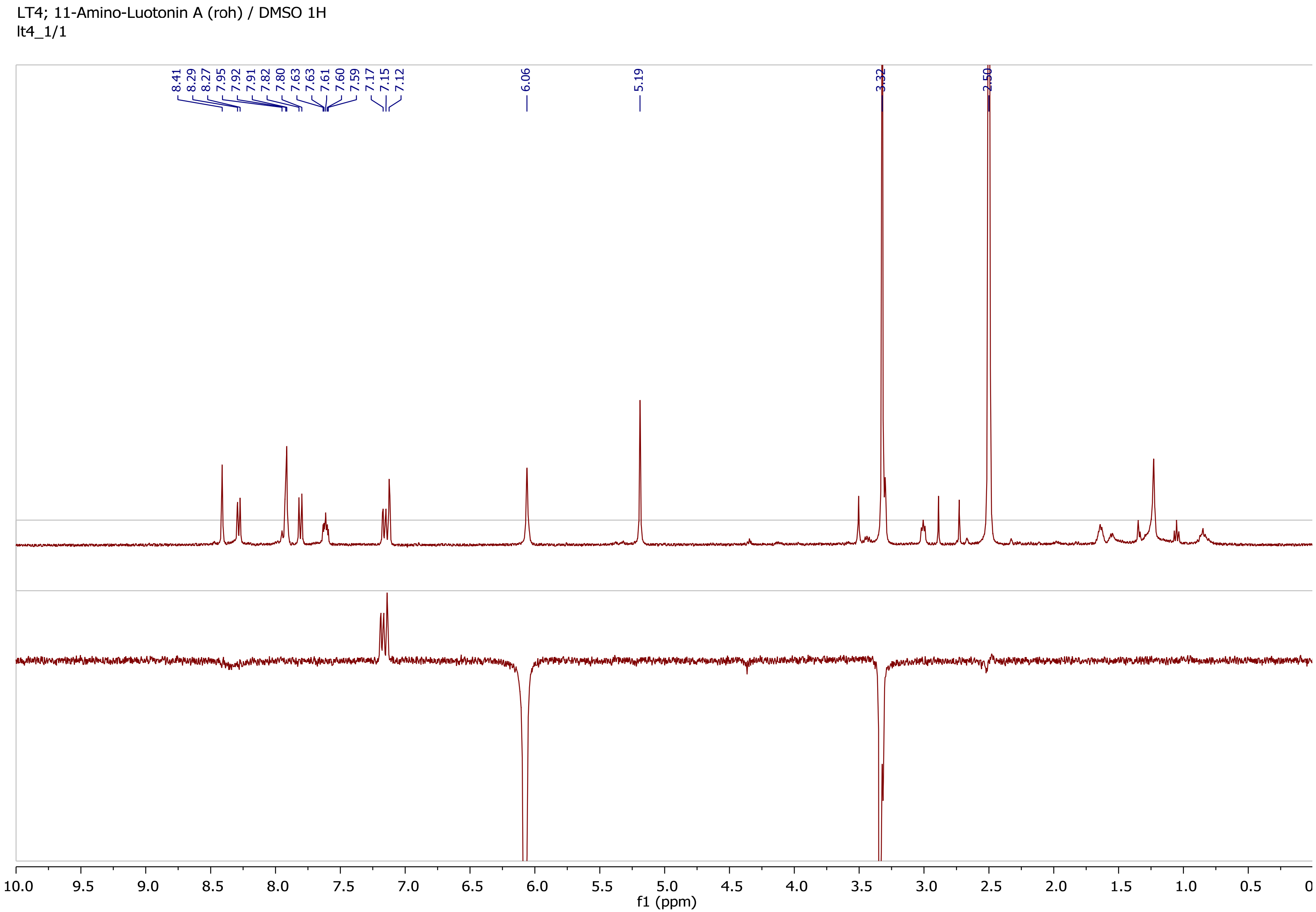

Figure S52. NOE difference spoectrum #2 of 3-aminoquinolino[2',3':3,4]pyrrolo[2,1-*b*]quinazolin-11(13*H*)-one (**10b**).

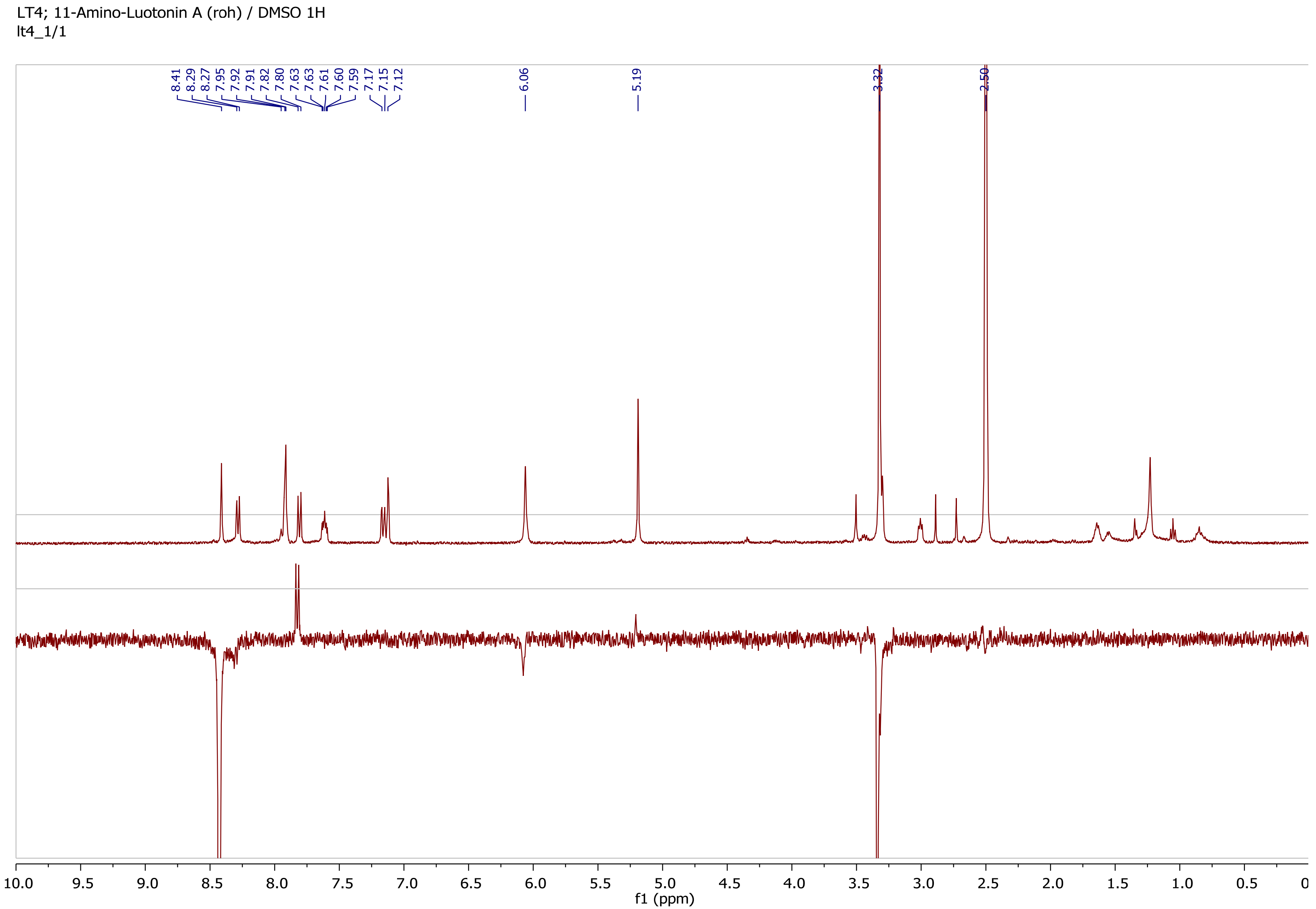

Figure S53. NOE difference spoectrum #3 of 3-aminoquinolino[2',3':3,4]pyrrolo[2,1-*b*]quinazolin-11(13*H*)-one (**10b**).

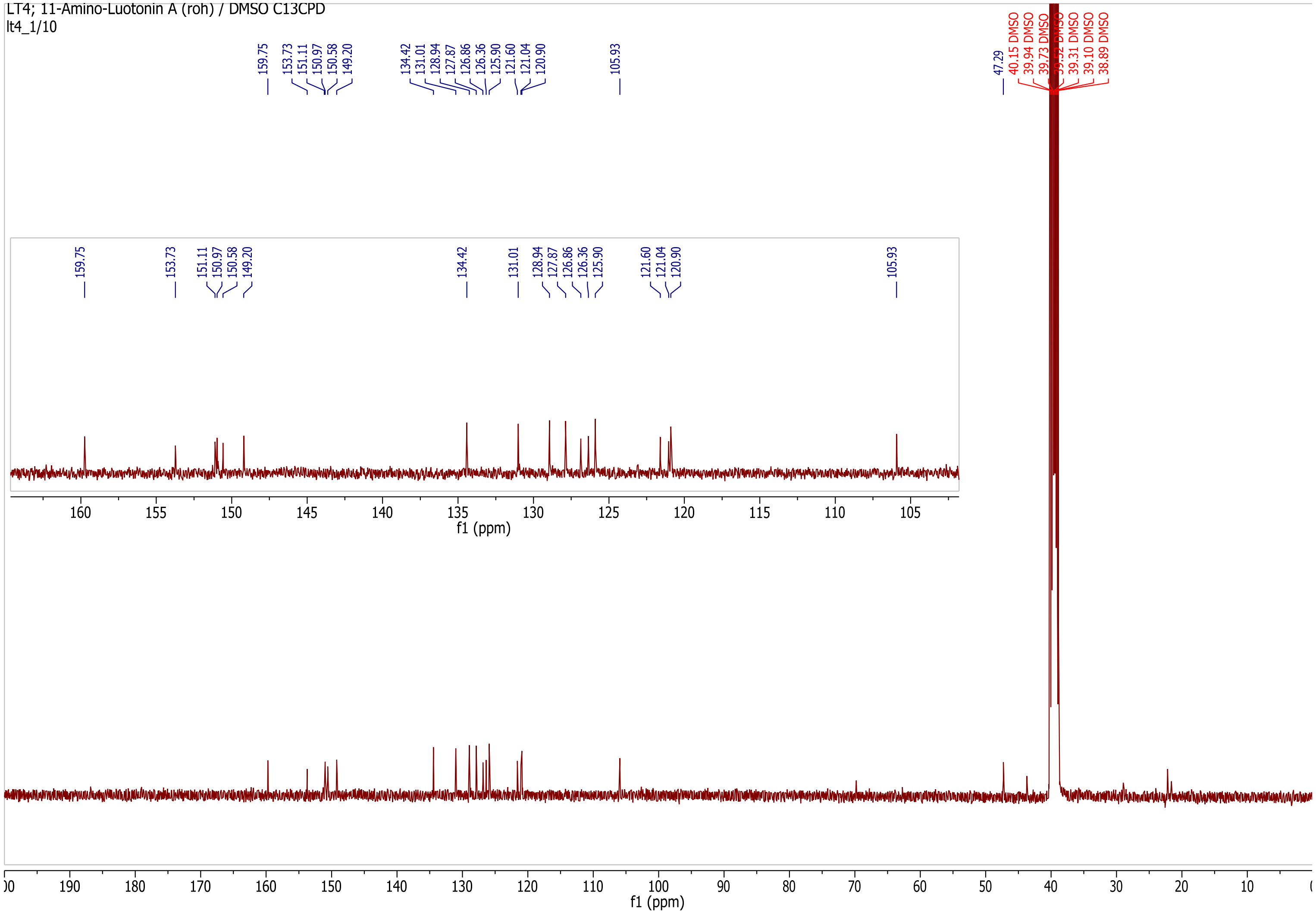

Figure S54. ^{13}C NMR spoectrum of 3-aminoquinolino[2',3':3,4]pyrrolo[2,1-*b*]quinazolin-11(13*H*)-one (**10b**).

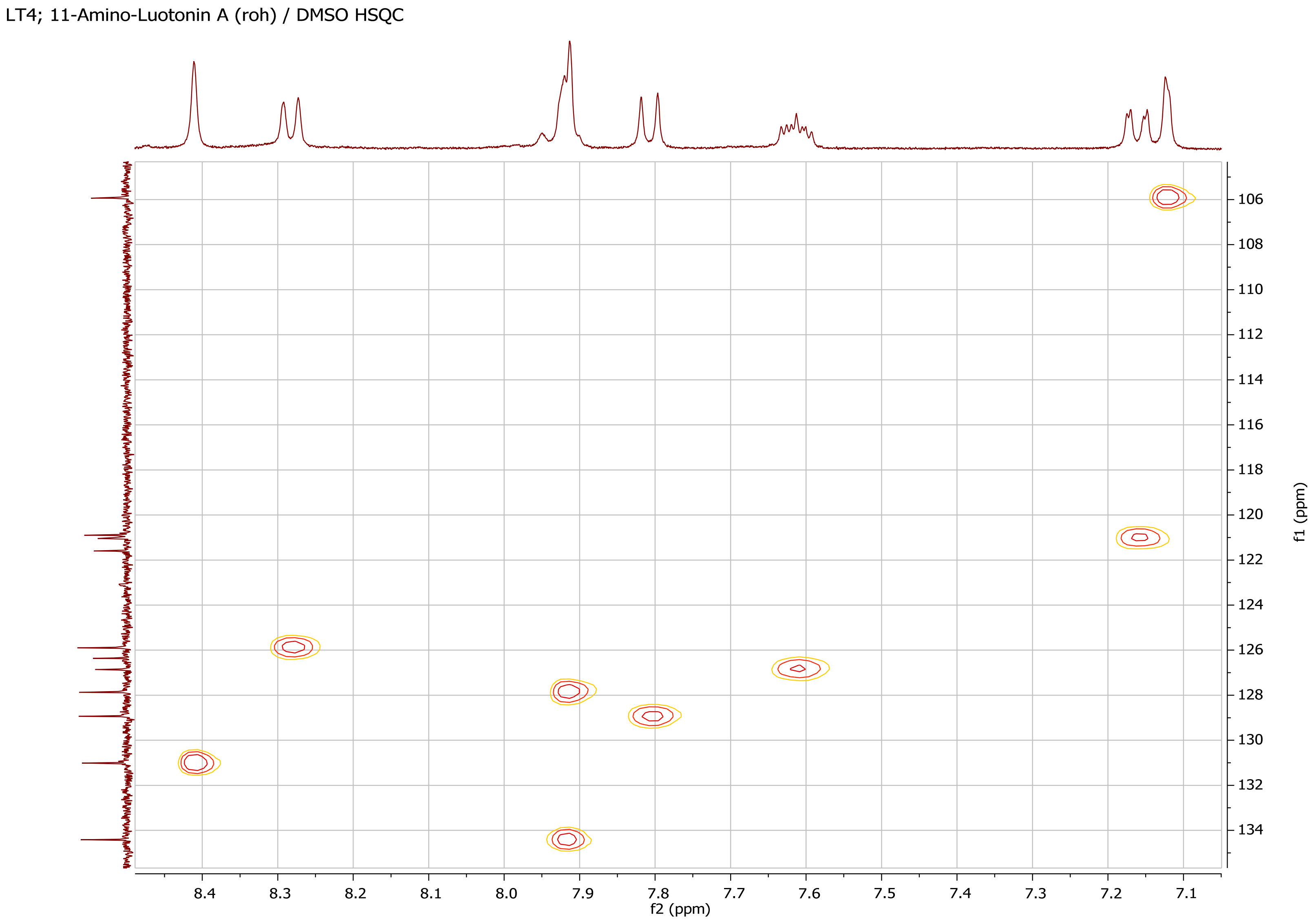

Figure S55. HSQC spoectrum of 3-aminoquinolino[2',3':3,4]pyrrolo[2,1-*b*]quinazolin-11(13*H*)-one (**10b**).

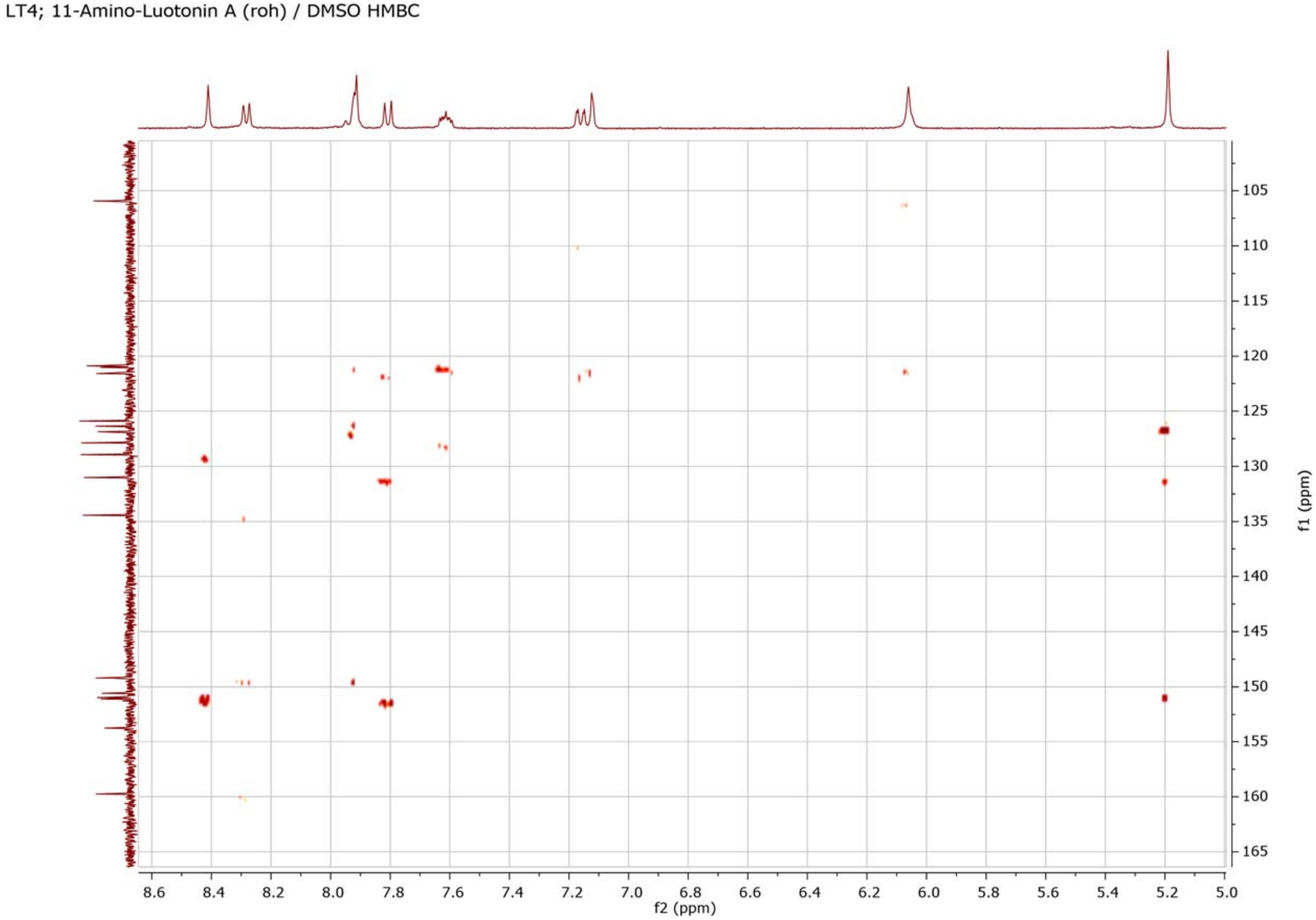

Figure S56. HMBC spoectrum of 3-aminoquinolino[2',3':3,4]pyrrolo[2,1-*b*]quinazolin-11(13*H*)-one (**10b**).

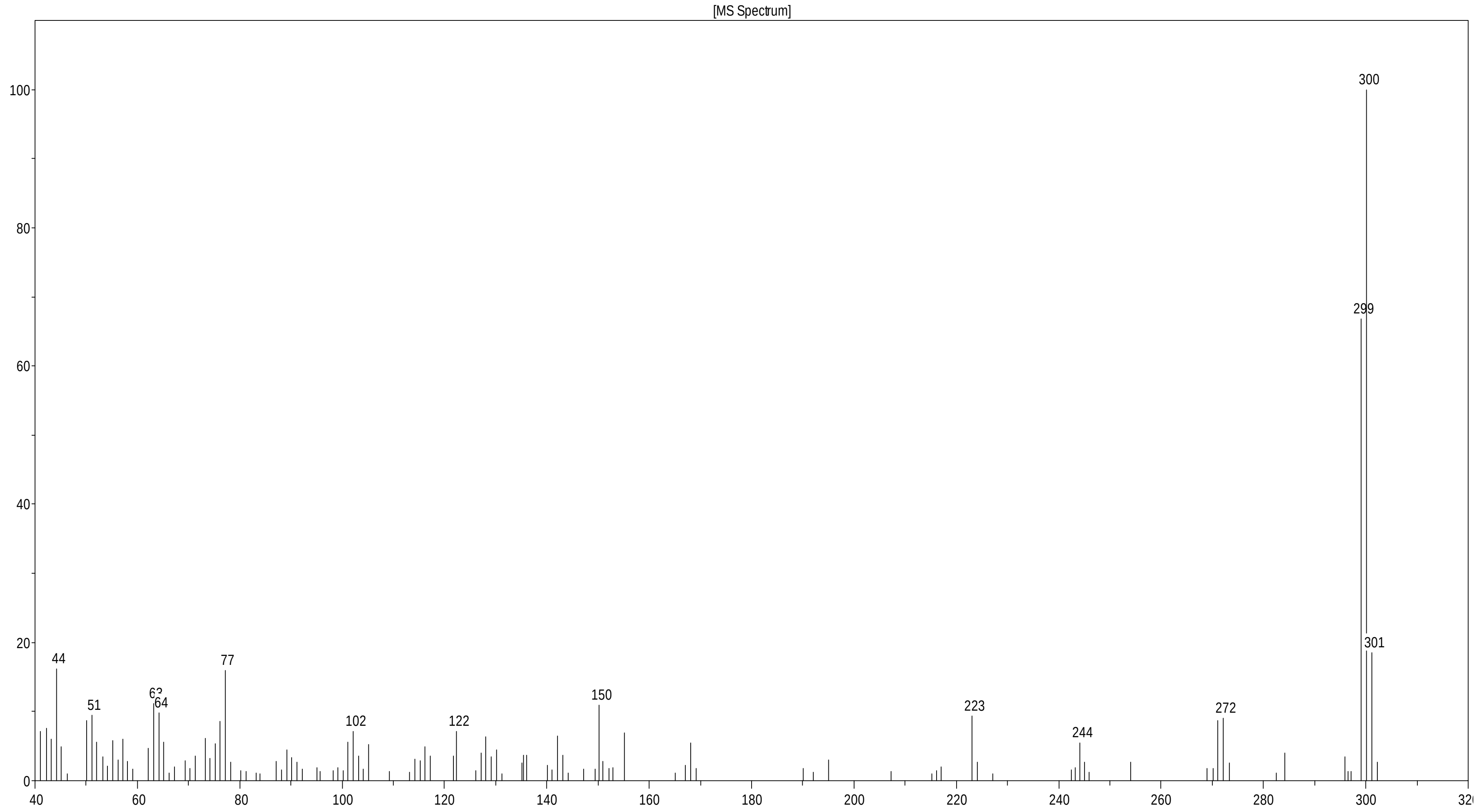

Figure S57. EI-MS of 3-aminoquinolino[2',3':3,4]pyrrolo[2,1-*b*]quinazolin-11(13*H*)-one (**10b**).
